# Transcriptome-module phenotype association study implicates extracellular vesicles biogenesis in *Plasmodium falciparum* artemisinin resistance

**DOI:** 10.3389/fcimb.2022.886728

**Published:** 2022-08-19

**Authors:** Kwesi Z. Tandoh, Oheneba C. Hagan, Michael D. Wilson, Neils B. Quashie, Nancy O. Duah-Quashie

**Affiliations:** ^1^ West African Centre for Cell Biology of Infectious Pathogens, Department of Biochemistry, Cell, and Molecular Biology, College of Basic and Applied Sciences, University of Ghana, Accra, Ghana; ^2^ Department of Parasitology, Noguchi Memorial Institute for Medical Research, College of Health Sciences, University of Ghana, Accra, Ghana; ^3^ Department of Epidemiology, Noguchi Memorial Institute for Medical Research, College of Health Sciences, University of Ghana, Accra, Ghana; ^4^ Centre for Tropical Clinical Pharmacology and Therapeutics, School of Medicine and Dentistry, College of Health Sciences, University of Ghana, Accra, Ghana

**Keywords:** transcriptomics, modules, association, artemisinin resistance, *Plasmodium falciparum*, extracellular vesicles

## Abstract

*Plasmodium falciparum* malaria is still an important disease in sub-Saharan Africa (sSA). Great strides have been made in its control spear-headed by artemisinin (ART)-based combination therapies (ACTs). However, concerns about the imminent spread of ART-resistant (ARTr) malaria parasites to sSA threaten gains already made. Attempts to mitigate this risk have highlighted the need to discover novel *P. falciparum* drug targets. Therefore, studies to deepen our understanding of the biology of *P. falciparum* are needed. The role of extracellular vesicles (EVs) in the biology of malaria parasites is not fully understood. Recently, the ART resistance-associated transcriptional profile has been reported to involve several biological processes connected to vesicular trafficking, proteotoxic stress, erythrocyte remodelling, and mitochondrial metabolism. We explored a role for EVs in developing the *P. falciparum* ARTr phenotype using bulk RNA sequencing of unsynchronized parasite cultures under untreated, 0.1% dimethyl sulfoxide and 700nM dihydroartemisinin treated conditions for six hours. As pathway and gene ontology analysis is limited in its curated knowledge repertoire on EVs biogenesis in *P. falciparum*, we used a modular (gene set) analysis approach to explore whether an EVs biogenesis module is associated with the ARTr phenotype in *P. falciparum*. We first generated well-defined EVs modules of interest and used statistical tools to determine differences in their expression among the parasite and treatment conditions. Then we used gene set enrichment analysis to determine the strength of the association between each EVs module of interest and the ARTr phenotype. This transcriptome-module phenotype association study (TMPAS) represents a well-powered approach to making meaningful discoveries out of bulk gene expression data. We identified four EVs module of interest and report that one module representing gene sets with correlated expression to PF3D7_1441800 – involved with EVs biogenesis in *P. falciparum* - is associated with the ARTr phenotype (R539T_DHA_treated versus R539T_untreated: normalized enrichment score (NES) = 1.1830174, FDR q-value < 0.25; C580R_DHA_treated versus C580R_untreated: NES = 1.2457103, FDR q-value < 0.25). PF3D7_1441800 has been reported to reduce EVs production when knocked out in *P. falciparum*. Altogether, our findings suggest a role for EVs in developing ART resistance and warrant further studies interrogating this association.

## Introduction

One of the pillars driving malaria control in sub-Saharan Africa (sSA) is the use of artemisinin (ART)-based combination therapies (ACTs) for effective management of identified malaria cases (WHO, 2015; WHO, 2021a). Although ACTs are still effective today, grave concerns have risen about their future efficacy because of the emergence of artemisinin-resistant (ARTr) parasites in South-East Asia (SEA) (Noedl et al., 2008; Dondorp et al., 2009) and their recent independent emergence in Rwanda and Uganda (Uwimana et al., 2020; Balikagala et al., 2021; Bergmann et al., 2021; Uwimana et al., 2021). This trajectory of events has made dire the need for efforts to provide alternatives to current ACTs. Preemptive risk mitigation activities include active molecular surveillance and novel antimalarial drug target discovery (WHO, 2021b). Therefore, to spur efforts toward novel malaria drug discovery and active molecular surveillance, our current understanding of the molecular mechanisms underpinning ARTr must be deepened.

Extracellular vesicles (EVs) are a heterogeneous group of membrane-bound vehicles reported to be important to the biology of *P. falciparum*, mainly within the context of parasite-parasite communication (Mantel and Marti, 2014; Marti and Johnson, 2016; Roditi, 2016). As very little was previously known about EVs biogenesis pathways in *P. falciparum*, exploratory transcriptome-phenotype association studies yielded little or no power to explore their role in ART resistance. Recently, Avalos-Padilla et al. reported that *P. falciparum* EVs biogenesis is accomplished by a functional endosomal sorting complex required for transport (ESCRT) activated by an alternative recruitment pathway involving the action of PfBro1 and PfVps32/PfVps60 proteins (Avalos-Padilla et al., 2021). This finding has made it possible for direct and targeted interrogation of the role of EVs in *P. falciparum* ART resistance.

ART resistance is understood to be undergirded by two broad molecular mechanistic motifs: an increased tolerance to ART-induced oxidative stress and reduced haemoglobin endocytosis to the digestive food vacuole. Both motifs are causally associated with variants in the propeller region of the *P. falciparum* kelch 13 gene (*PfK13*) and have been extensively reviewed recently by Siddiqui and others (Siddiqui et al., 2021). Whether EVs can be implicated in the expression of the ARTr phenotype is yet to be empirically explored (Tandoh et al., 2021). The closest sliver of evidence was established within the context of parasite-parasite communication and suggested that EVs may be able to transfer the ARTr phenotype to ART susceptible parasites (Regev-Rudzki et al., 2013). Additionally, recently conducted gene expression studies to gain insight into the molecular mechanisms undergirding ART resistance reported the involvement of many biological processes that included cell-cycle periodicity, the unfolded protein response, protein degradation, mitochondrial and redox metabolism, erythrocyte remodelling and vesicular trafficking (Mok et al., 2021; Zhu et al., 2022).

In this study, we posed the research question: is EVs biogenesis associated with the ARTr phenotype? We conducted an EVs targeted exploratory analysis of the transcriptome of two ARTr and one ART susceptible parasite line under three conditions (untreated, 0.1% dimethyl sulfoxide (DMSO) and 700nM dihydroartemisinin (DHA) treatment conditions for six hours) to interrogate this question. Using the novelty and higher discovery power of a transcriptome-module phenotype association study (TMPAS), we show that an EVs module of interest is associated with the ARTr phenotype R539T_DHA_treated versus R539T_untreated: NES =1.1830174, FDR q-value < 0.25; C580R_DHA_treated versus C580R_untreated: NES = 1.2457103, FDR q-value < 0.25). We also report on the heterogeneous nature, with respect to gene expression profile, of the ARTr population of *P. falciparum* parasites. Our findings add knowledge that will help shape future studies into EVs biogenesis and ART resistance and will contribute to novel antimalarial drug discovery efforts. Most importantly, our data implicate EVs in developing the ARTr phenotype outside the already reported theme of parasite-parasite communication. Further studies interrogating this motif are merited.

## Method

### Parasite lines and culture conditions

Transgenic parasite lines (C580R and R539T) were generated using CRISPR/Cas9 genome editing of the *PfK13* locus in a DD2 parental line. The plasmid utilised for the genome editing targeting the *P. falciparum* kelch 13 gene, pDC2-cam-co. Cas9-U6-hDHFR was originally designed by Marcus Lee of Sanger Institute. The components included a codon optimised Cas9 sequence with a nuclear localisation sequence driven by the *P. falciparum* calmodulin promoter and Pfhsp86 3’ UTR (untranslated region) terminator. It had a *P. falciparum* U6 cassette for gRNA (guide RNA) expression with a *BbsI* restriction site for cloning in the gRNA. A human dihydrofolate reductase (*hdhfr*) gene sequence expressed using the *P. chabaudi* dihydrofolate reductase-thymidylate synthase (*PcDT*) 5’ UTR and *P. falciparum* histidine-rich protein 2 (*Pfhrp2*) 3’ UTR was used for selection during transfection using WR22910 - an ampicillin resistance cassette for selecting successfully transformed *Escherichia coli* after the plasmid transformation. It also possesses an *EcoR1/AatII* restriction site for cloning in the donor DNA sequence. Successfully genome-edited parasites were confirmed using Sanger sequencing of polymerase chain reaction (PCR) products, with the primers for the PCR designed to amplify beyond the donor DNA to preclude amplification of episomally retained plasmids. Additionally, whole-genome sequencing was performed, and the sequence was compared with the parental genome (**Supplementary Figures 1–3**).

We used the known R539T ARTR variant found in SEA and the C580R *PfK13* variant first reported to be present in the forest and coastal zones of Ghana (Ariey et al., 2014; Miotto et al., 2015; Ménard et al., 2016; Matrevi et al., 2017). Our rationale was to validate that the *PfK13* variant (C580R) of Ghanaian provenance did confer the ARTr phenotype.

*P. falciparum* parasites were cultured at 5% hematocrit in human O- red blood cells (RBCs) and culture media comprising RPMI 1640 (Thermo Fisher Scientific) supplemented with 0.5% (w/v) Albumax II (Invitrogen), 50 mg/L hypoxanthine, 0.225% NaHCO3, 25 mM HEPES, and 10 mg/L gentamycin. Parasites were cultured at 37°C in 5% O2, 5% CO2, and 90% N2 (Quashie et al., 2013).

### Parasite sensitivity assays to dihydroartemisinin

A SYBR Green 1 fluorescent-based *in vitro* drug sensitivity assay was used to assess the susceptibility of the *P. falciparum* parasites, used in this study, to ART. This assay was used to determine the 50% inhibitory concentrations (IC50) defined as the concentration of dihydroartemisinin (DHA) (the *in vivo* metabolically active form of ART) inhibiting parasite growth by 50%. (Johnson et al., 2007; Machado et al., 2016). Briefly, we exposed unsynchronized *in vitro* parasite cultures, in triplicate, across a range of dihydroartemisinin concentrations (62.5pM to 16nM) for 72 hours. Parasites were diluted to 1% parasitemia in 2% hematocrit, and a 100μl parasite culture mix was pipetted into a drug-prefilled 96-well plate in triplicates to obtain a final volume of 200μl in each well. The control parasite cultures devoid of the drug were referred to as having 100% growth. The plates were incubated for 72 hours with a gas mixture of 93% nitrogen, 5% CO2, and 2% oxygen. We measured growth by SYBR Green I staining (ThermoFisher, https://www.thermofisher.com) and performed a photometric assessment using a SpectraMax M5 plate reader (Molecular Devices) to measure fluorescence with excitation and emission wavelength bands at 485 and 530nm, respectively. IC50 for DHA was estimated using nonlinear regression with the log drug concentration versus growth rate response in a 4-parameter variable slope fitting dose-response curve (generated from the nine-point dilution series carried out in triplicates) using the R package drc (https://cran.r-project.org/web/packages/drc/drc.pdf), version 3.0-1 (Ritz et al., 2015) and comparison between parasite lines was done by Student t-test. All analysis was performed in R version 4.0 (R Core Team, 2021). A p-value of less than 0.05 was considered indicative of a statistical significance.

The 0–3-hour ring-stage survival assay (RSA) was performed as described previously with few modifications. The RSA is an *in vitro* assay for measuring ART resistance and is of very practical utility in this regard because IC50’s for both ARTr and susceptible parasites tend to have no statistically significant differences (Witkowski et al., 2013; Ariey et al., 2014). In brief, highly synchronized 0–3-hour post-invasion rings were exposed to 700nM DHA for 6 hours, followed by removal of the drug. A parallel non-DHA exposed (0.1% DMSO exposed) set of isolates were used as controls. After 72 hours after initial drug treatment, parasitemia was assessed by Giemsa-stained smear microscopy of thin smears (parasite density per ≥ 10,000 red blood cells). The ring-stage survival percentage was calculated as the fraction of surviving DHA-treated parasites over the DMSO-treated control of the same parasite line (with a parasite growth rate of > 1.5%). An RSA survival percentage >1% is indicative of ART resistance. The RSA for each parasite line was determined with triplicates.

### DHA and DMSO exposures to parasite cultures

We harvested a total of 27 samples from three *P. falciparum* lines *in vitro* cultures (**Supplementary Table 1**). We used a DD2 parent line, and two engineered ART resistant lines on the DD2 background (R539T and C580R). We cultured *P. falciparum* parasites in human O-negative RBCs using standard methods (Trager and Jensen, 1976).

Once parasitemia reached 3-5% parasitemia, unsynchronised parasite cultures were exposed to 700nM DHA or 0.1% (v/v) DMSO solvent control for six hours. Parallel culture plates were also left untreated. After 6 hours of DHA or DMSO treatment, we centrifuged the parasite culture mix at 2500rpm for five minutes to harvest the infected erythrocytes. Subsequently, parasites were liberated from host erythrocytes by treatment with 0.015% (w/v) saponin for five minutes at room temperature (Gibbons et al., 2018). After that, liberated parasites were washed twice with 1x PBS (137mM NaCl, 8mM KCl, 10mM Na2HPO4, 2mM KH2PO4, pH 7.4) to remove any host cell debris and residual drug. The parasites were then pelleted and stored at -80°C in an Eppendorf tube (1.5ml) containing 1mL Trizol reagent (Fisher Scientific, Hampton, NH) before RNA extraction (Gibbons et al., 2018).

### RNA extraction, library preparation and next-generation sequencing

Sequencing was performed at Single Cell Discoveries, a sequencing service provider located in the Netherlands using an adapted version of the CEL-seq protocol. Total ribonucleic acid (RNA) was extracted from TRIzol-preserved parasites after thawing at room temperature using the standard TRIzol (Invitrogen) protocol and used for library preparation and sequencing. The extracted RNA was dissolved in 100µl RNase-free water and DNA removed by DNase I digestion, after which the purified RNA was eluted into 50µl RNase-free water. Qubit High Sensitivity RNA assay (Thermo Fisher Scientific) was used to quantify the purified RNA, and RNA integrity was determined on an Agilent Bioanalyzer (Agilent Genomics, Waldbronn, Germany). Messenger RNA (mRNA) was processed as described previously, following an adapted version of the single-cell mRNA sequencing protocol of CEL-Seq (Hashimshony et al., 2012; Simmini et al., 2014). In brief, samples were barcoded with CEL-seq primers during reverse transcription and pooled after second strand synthesis. The resulting complementary DNA (cDNA) was amplified with an overnight *in vitro* transcription reaction. From this amplified RNA, sequencing libraries were prepared with Illumina Truseq small RNA primers. The DNA library was paired end sequenced on an Illumina NextseqTM 500, high output, with a 1x75 bp Illumina kit (R1: 26 cycles, index read: 6 cycles, R2: 60 cycles). Read 1 was used to identify the Illumina library index and CEL-Seq sample barcode. Read 2 was aligned to the *Plasmodium falciparum* ASM276v2 reference transcriptome using BWA, version 0.7.17-r1188 (Li and Durbin, 2009). We aligned sequence reads to the *P. falciparum* transcriptome because our objective was to explore the well-annotated reference transcriptome and perform differential expression analysis within known genes (Conesa et al., 2016). Reads that mapped equally well to multiple locations were discarded. Mapping and generation of count tables were done, after quality control using *fastqc* (https://www.bioinformatics.babraham.ac.uk/projects/fastqc/) version 0.11.9, and converted to “bam” format using *samtools* (https://github.com/samtools/samtools) version 1.10, using the MapAndGo script (https://github.com/anna-alemany/transcriptomics/tree/master/mapandgo).

### Data analysis

The R package DaMiRseq version 2.0.0 (Chiesa et al., 2018) was used to explore the raw count data for batch effect, identify surrogate variables that may act as confounders and adjust the count data for downstream analysis. Lowly expressed genes (minimum count of 10) and samples with less than 70% of genes expressed were filtered out before normalization using variance stabilizing transformation (vst). The variance stabilizing transformation (vst) function to normalize and transform the count matrix. The vst is computed from the fitted relationship between the variance and the mean. This is used to normalize the count data to generate values of approximately equal variance along with the range of mean values. The vst transformation also normalizes for library size. Its use precludes the need for the generation of TPM (transcripts per million) normalized count data. Following pre-processing and transformation of the count data filtering by the coefficient of variation, set at a correlation coefficient of greater than or equal to 0.7, was done. Sample filtering was done using a cut-off spearman correlation coefficient of ρ > 0.8. The normalized and transformed count data was used for the exploratory data analysis for examining the quality of the data before downstream modular and differential gene expression analysis.

### Modular analysis

Modular analysis involves the generation of a gene set (module) comprised of gene members with correlated expression and the determination of whether the module of interest is associated with the biological process (phenotype) of interest. The generated “repertoire of co-clustering gene sets” or transcriptional module is used to “simplify” large scale analysis and interpretation of transcriptional data (Chaussabel and Baldwin, 2014). It is a strategy largely borrowed from the field of immunology and was leveraged here to explore any association between EVs and ARTr.

We used the R package Weighted correlation network analysis (WGCNA version 1.70-3, http://www.genetics.ucla.edu/labs/horvath/CoexpressionNetwork/Rpackages/WGCNA) for our modular analysis using customized scripts (Langfelder and Horvath, 2008). WGCNA R software package is a compendium of R functions for performing various aspects of weighted correlation network analysis. It highlights a systems biology approach for describing the correlation patterns among genes in a gene expression data set. It can identify clusters/modules of highly correlated genes that can be used as the gene sets for determining associations with a biological phenotype of interest.

Biological meaning was distilled from our EVs modules of interest by using the gene sets as our *a priori* list for functional class scoring in the Gene Set Enrichment Analysis (GSEA) software (version 4.1.0) for enrichment analysis and gene ontology (GO) analysis (Subramanian et al., 2005). GSEA is an analytical method for distilling biological meaning from gene expression datasets. Unlike traditional differentially expressed gene (DEG) and GO analysis pipelines, GSEA’s discovery power is hinged on its use of gene sets instead of single gene units. Its approach to gene expression analysis keeps pace with the systems biology motif that biological phenomenon and/or phenotypes are results of multiple genes working in concert as opposed to the reductionist motif that assumes a single or few genes largely account for the biological phenomenon of interest (Bruggeman and Westerhoff, 2007). It uses an *a priori* defined gene set(s) (we used EVs module of interest defined as sets of genes with correlated expression with known EVs biogenesis genes. These EVs modules of interest do not necessarily belong to the same biological pathway) to interrogate the ordered count data of an RNA sequencing experiment and outputs the gene set with significant association to the biological phenomenon of interest.

GSEA highlights the following deficiencies of the traditional DEG analysis pipeline and bypasses them. Traditional analysis approaches the problem of distilling meaning from the gene expression data set by focussing on a few genes that show the largest differences in expression. The over-representation analytical methods are seminal examples of this conventional approach. However, the sensitivity of this approach is stymied by the following problems: If the relevant gene(s) responsible for the biological phenomenon of interest do so by small changes in gene expression, then it will not be detected at all or will be detected with no statistical significance. On the other hand, this approach can output a tall list of statistically significant genes involved in a plethora of divergent biological processes. This lack of a unifying biological theme may make meaningful interpretation impossible. Finally, precision has been reported to be a problem with the traditional gene expression approach as subsequent analysis by different groups, of the same data set, may yield strikingly discordant results (Subramanian et al., 2005). GSEA, as a functional class scoring method, is designed to overcome these challenges by feeding an *a priori* defined gene set and determining whether members of this gene set tend to occur at the top or bottom of the ordered list (or whether they are randomly distributed) of genes in the count data. If they do, then the gene set is associated with the phenotypic distinction used.

GSEA calculates three key elements: 1) the calculated enrichment score (ES) indexes the strength of the correlation between a gene set and the entire ranked list of count data. ES is calculated by walking down the ranked list of count data and assigning an increasing running-sum statistic when a gene in the gene set is encountered and decreasing the running-sum statistic when genes not in the gene set are encountered. The magnitude of the running-sum statistic depends on the strength of the gene set-phenotype correlation. Thus the ES is the maximum deviation from zero assigned during the random walk down the ranked count list and corresponds to a weighted Kolmogorov–Smirnov-like statistic (Subramanian et al., 2005) 2) the significance level of the calculated ES is estimated using an empirical phenotype-based permutation test and calculates the P-value of the ES. 3) adjustment is made for multiple testing and normalizes for variance in the gene sets used to yield the normalized ES (NES). Finally, the false discovery rate (FDR) (estimate of the probability that an NES represents a false positive finding) is calculated to control the proportion of false discoveries (Subramanian et al., 2005).

### Identification of differentially expressed genes between conditions

The raw count data was fed into the DESeq2 R package (version 1.28.1) (Love et al., 2014) for the differential expression analysis. Following the generation of the DESEq2 object, the result function was used to generate the differentially expressed genes for each paired condition. This function uses a generalized linear model (GLM) to model the contribution of each gene to the conditions. This GLM is modelled using a discrete probability distribution such as negative binomial or Poisson’s distribution. This is because RNA sequencing data is discrete count data and does not follow a normal distribution as negative integers are not expected. Then Wald’s statistic and t-test (with p-value) are used to weight the contribution of each gene in the model as significant or insignificant.

### Gene ontology analysis

This analysis was done by uploading modules and DEG to PlasmoDB (https://plasmodb.org/plasmo/app, release 58) and using the site’s GO pipeline for analysis. REVIGO was used to generate the GO summaries. REVIGO is a web server that summarizes the enriched gene functional categories lists of GO terms by finding a representative subset of the terms using a simple clustering algorithm that relies on semantic similarity measures (Supek et al., 2011).

## Results

### *P. falciparum* parasites susceptibility to DHA

We first used the RSA and IC50 assays, described earlier, to confirm the drug susceptibility of the parasite strains to DHA. The *in vitro* RSA was used to measure the susceptibility of the parasite lines to DHA. A read out of greater than 1% is indicative of resistance to ART. The RSA assay showed that C580R and R539T were ARTr, with median RSA survival rates of 3.5% (IQR, C580R = 3.25-3.75; IQR, R539T=3.5-4.05). DD2 was artemisinin susceptible with a median survival rate of 0.5% (IQR, = 0.5 – 0.65) (**Supplementary Figure 4**). The IC50 assay measures the drug concentration at which 50% of parasites are killed by ART. Drug susceptibility by IC50 assay showed no statistical significant differences among the three parasite lines (p-value, Kruskal-Wallis test = 0.37). (**Supplementary Figure 5**).

### Quality check for integrity of extracted RNA and mapped reads of samples analyzed

For each of the three parasite lines (DD2, C580R and R539T), the following replicates were used for the three treatment conditions studied: four replicates per parasite line for the 700nM DHA condition, three replicates per parasite line for the 0.1% DMSO control condition and two replicates per parasite line for the untreated condition (**Supplementary Table 1**). Following ribonucleic acid (RNA) extraction, the quality of RNA was assessed using the RNA integration number (RIN) metric. RIN is determined, following bioanalyzer electrophoretic assay, by the ratio of the 18s and 28s ribosomal peaks to the total area under the curve. Most of the RNA extracted from samples was of good quality (**Supplementary Figure 6**). The overall mapping quality across all samples was good. The mapping depth (mean read counts per sample) was 3035536.93, and reads were mapped to at least 5000 genes for each of the samples. External RNA Controls Consortium (ERCC) spike-in controls levels were similar across all samples. ERCC helps control for variation in RNA expression data arising from factors such as RNA quality and yield and sequencing errors (Tong et al., 2016) (**Supplementary Figure 7**).

Lowly expressed genes (minimum count = 10) and samples with less than 70% of genes expressed were filtered out (997 features removed) after data pre-processing and normalization using DaMiRseq (Chiesa et al., 2018). Finally, two surrogate variables (Sv) that explained 95% of the variance in the data set were identified and adjusted for in the data. Sv’s are covariates generated from high-dimensional data and used to adjust for hidden sources of variation. The DaMiR.corrplot function produced a correlation plot where significant correlations (significance level = 0.01) are shown within colored circles (blue or red gradient). As expected, the correlation between class, and the other covariates, was not significant (**Supplementary Figure 8**). Overall variation in relative log expression across samples was identical and the effect of surrogate variables on the count data set was adjusted (**Supplementary Figure 9**).

### A module of EVs interest is associated with ART resistance in *P. falciparum*

The transcript count data was fed into the R package, WGCNA (Langfelder and Horvath, 2008) to generate modules made up of gene transcripts with correlated expression profiles. Seventeen modules were then selected based on the statistical significance (adjusted p-value < 0.05) of their expression profile correlation. Gene transcripts were converted to gene identities for each of these seventeen modules (**Supplementary Table 2**). As relatively little is known about EVs biogenesis in *P. falciparum* biology, we approached the problem of determining whether the transcript profile of ART resistant *P. falciparum* is associated with EVs biogenesis by defining EVs biogenesis modules. Four modules (modules 0, 1, 3 and 17) contained our genes of interest implicated in the biogenesis and export of EVs (**Table 1**; **Supplementary Table 2**) (Regev-Rudzki et al., 2013; Avalos-Padilla et al., 2021). We refer to these modules as our modules of EVs interest and they comprise genes with correlated expression to known genes in the *P. falciparum* EVs biogenesis pathway (**Table 1**).

**Table 1:** Genes of interest (GOI) involved in the biogenesis and export of extracellular vesicles, (EVs) in *P. falciparum*.

Gene of interest	Protein ID	Alternative name	Literature source	Module of EVs interest
PF3D7_1224200	CZT99401	*PfBRO1*	(Avalos-Padilla et al., 2021)	Not found
PF3D7_1243500	CZT99591	*PfVps32*		ME 1*
PF3D7_1441800	CZU00114	*PfVps60*		ME0
PF3D7_0816200	CAD51211	*PfVps2*		ME0
PF3D7_0906100	CAD51746	*PfVps46*		ME3*
PF3D7_1121600	CZT98878	*PfPTP*	(Regev-Rudzki et al., 2013)	ME17**

*0.05 < p-value < 0.1.

**p-value < 0.05.

P-values were determined by comparing the module of EVs interest expression among the various treatment conditions of the parasite lines using the Kruskal-Wallis test. GOI were identified by review of the literature on EVs in *P. falciparum*. Module of EVs interest was determined using the weighted correlation network analysis (WGCNA) R package by first generating modules of genes with statistically significant correlated expression across all samples (p-value < 0.05) and then identifying the modules containing the GOI.

**Figure 1:**
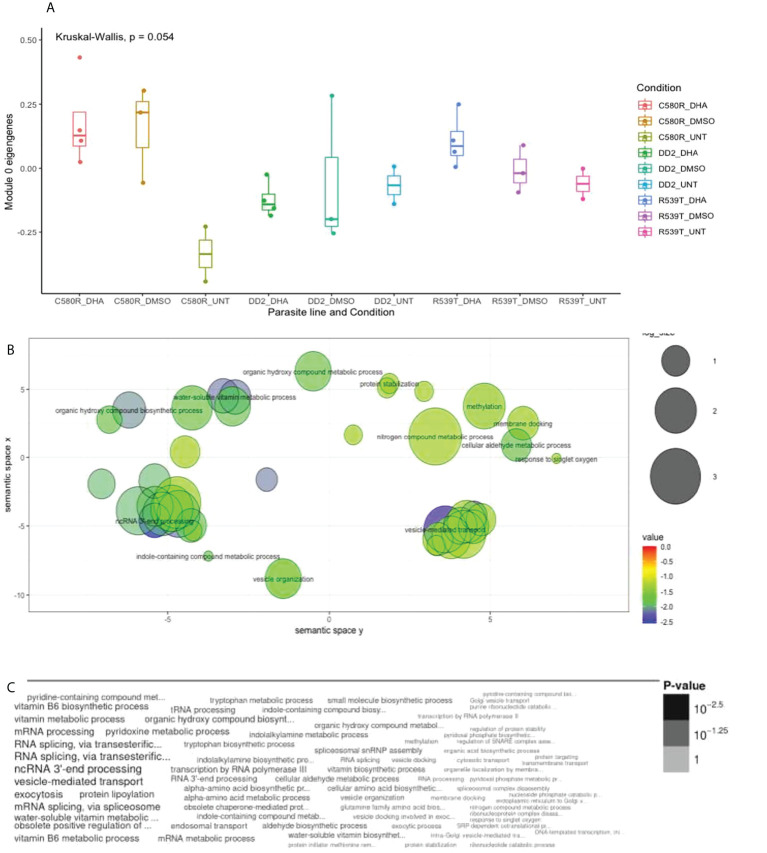
Properties of EVs module of interest module 0 (ME0). ME0 was uploaded to PlasmoDB (https://plasmodb.org/plasmo/app) and analyzed with the platform’s gene ontology (GO) pipeline. **(A)** Comparison of ME0 between the three treatment conditions pooled for all parasite lines (DD2, C580R and R539T). The Kruskal-Wallis test was used to determine the statistical significance of the difference in expression of ME0 among the conditions (0.05 < p-value < 0.1). **(B)** GO (biological processes) output for ME0 summarized using REVIGO (http://revigo.irb.hr) scatterplot visualization option. Each circle denotes the representative cluster of the GO biological process associated with the ARTr phenotype and is derived by multidimensional scaling and redundancy reduction of the GO sematic similarities. The size of the circles denote the frequency of the GO term in the *P. falciparum* GO database (updated in November 2021) with larger circles denoting more general terms. The colors (red to blue range) indicate the statistical significance of the association’s strength. “Vesicle mediated transport” was one of the biological processes enriched **(C)** Word cloud summary of the GO biological processes associated with the ARTr phenotype. EVs, extracellular vesicles; ARTr, artemisinin resistant.

Module 0 comprised 693 genes (**Supplementary Table 2**) and showed differences in expression between treatment conditions (0.05 < p-value < 0.1) (**Figure 1**). GO analysis using the online PlasmoDB(https://plasmodb.org/plasmo/app) and revigo (http://revigo.irb.hr) platform (Supek et al., 2011) showed enrichment for biological processes related to vesicle-mediated transport (p-value = 0.00327114166063; adjusted p-value = 0.474821139088) (**Figure 1**; **Supplementary Table 3**). Modules 1 (ME1), 3 (ME3) and 17 (ME17) were made of 1119, 363 and 67 genes respectively (**Supplementary Table 2**) and showed differences in expression between treatment conditions (0.05 < p-value < 0.1). GO analysis showed enrichment for translation and proteasomal ubiquitin-independent protein catabolic process in ME3 and cell cycle, nuclear chromosome segregation processes and phosphatidylinositol-3-phosphate biosynthetic process in ME17 (**Supplementary Figures 10–12**; **Supplementary Tables 4–6**).

Next, we used the functional class scoring GSEA version 4.1.0 software (Subramanian et al., 2005) in a TMPAS analysis to determine the association of each of the17 modules (including the four EVs modules of interest) with the ARTr phenotype. We leveraged on the ability of this method to determine even a small association of our modules (in particular our EVs modules of interest) with the ART resistance phenotype of interest. GSEA requires 3 files as input: a phenotype data file, a gene set file, and an expression data file (https://www.gsea-msigdb.org/gsea/doc/GSEAUserGuideFrame.html). Phenotype data was generated from **Supplementary Table 1** and formatted in the categorical class format (*.cls). **Supplementary Table 2** was formatted in the gene matrix file format (*.gmx) to generate the gene set file. Finally, we entered a normalized and *vst* transformed (using DESeq2 version 1.28.1) count matrix with gene expression ranked according to their correlation with the ART resistance phenotype of interest as our expression data set or gene list (**Supplementary Table 7**). The expression data set was formatted in gene cluster text file format (*.gct).

GSEA determines whether members of our modules are randomly distributed throughout the expression data or are differentially found at the top or bottom (indicative of association to ARTr). An enrichment score (ES) is calculated for each module that captures the strength of its association with the ARTr phenotype. The ES is calculated by the GSEA program and reflects the maximum deviation from zero in its random walk through the ranked genome-wide expression list. The ES corresponds to a weighted Kolmogorov–Smirnov-like statistic. GSEA also estimates the statistical significance of the association using an empirical phenotype-based permutation test procedure and corrects for the varied sizes of the modules of interest and multiple hypothesis testing by calculating the normalized enrichment score (NES) and false discovery rate (FDR), respectively (Subramanian et al., 2005). GSEA returns the strength of the association between each module of interest and the ART resistance phenotype. We identified module 0 (containing the EVs biogenesis genes PF3D7_1441800 and PF3D7_0816200) (**Table 1**) and module 7 as associated with the ARTr phenotype in *P. falciparum* (**Table 2**).

**Table 2:** Association of modules with the ARTr phenotype. Only 2 out of the 17 modules tested - modules 0 and 7- were associated with the ARTr phenotype.

Treatment conditions compared	Module	Upregulated in condition	Normalized enrichment score (NES)	False discovery rate q-value
R539T_DHA_treated versus R539T_untreated	Module 0	R539T_DHA_treated	1.1830174	0.23794872*
	Module 7	R539T_untreated	-1.2690192	0.14516129*
R539T_DHA versus R539T_DMSO_treated	Module 0	R539T_DMSO_treated	-0.78877187	0.859322
	Module 7	R539T_DMSO_treated	1.141659	0.43728814
R539T _DHA_treated versus DD2_DHA_treated	Module 0	DD2_DHA_treated	-1.1220989	0.28233033
	Module 7	R539T _DHA_treated	0.6829786	0.9447514
C580R_DHA_treated versus C580R_untreated	Module 0	C580R_DHA_treated	1.2457103	0.14403293 *
	Module 7	C580R_untreated	-2.4335837	0.0*
C580R_DHA_treated versus C580R_DMSO_treated	Module 0	C580R_DHA_treated	0.7299921	0.891
	Module 7	C580R_DHA_treated	1.2448145	0.253
C580R_DHA_treated versus DD2_DHA_treated	Module 0	C580R_DHA_treated	1.0806667	0.35456595
	Module 7	C580R_DHA_treated	1.088161	0.686584
DD2_DHA_treated versus DD2_untreated	Module 0	DD2_DHA_treated	1.1802711	0.22940503*
	Module 7	DD2_untreated	-2.2366247	0.0*
DD2_DHA_treated versus DD2_DMSO_untreated	Module 0	DD2_DMSO_treated	-1.1928045	0.33333334
	Module 7	DD2_DMSO_treated	-0.9836254	0.51960784

Module 0 is an EVs module of interest comprising 693 genes with correlated expression with the PF3D7_1441800 gene (*PfVps60)* involved in EVs biogenesis in *P. falciparum*. Module 7 is made up of 182 genes. The normalized enrichment score (NES) is calculated by normalizing the enrichment score for gene set size and represents the association of the modules with the treatment conditions compared. A positive NES indicates module enrichment and association with the first treatment condition and a negative NES indicates module enrichment and association with the second treatment condition. The higher the NES the stronger the association with the module. False discovery rate (FDR) was used to correct for multiple testing. Statistical significance (*) was designated at an FDR q-value < 0.25. Module 0 was significantly upregulated in the DHA-treated condition for both ARTr (C580R and R539T) and ART sensitive (DD2) parasite lines for the DHA-treated versus untreated condition. For the condition DHA treated versus DMSO-treated, module 0 was upregulated in the DMSO-treated condition for R539T and DD2 parasite lines, and in the DHA-treated condition for C580R line. Comparison of the DD2_DHA_treated condition with C580R_DHA_treated and R539T_DHA_treated treatment conditions showed that module 0 was upregulated in the C580R_DHA_treated condition and the DD2_DHA_treatment conditions respectively. Module 7 was significantly upregulated in the untreated condition for both ARTr (C580R and R539T) and ART sensitive (DD2) parasite lines for the DHA-treated versus untreated condition. For the condition DHA treated versus DMSO-treated, module 7 was upregulated in the DMSO-treated condition for R539T and DD2 parasite lines, and in the DHA-treated condition for C580R line. Comparison of the DD2_DHA_treated condition with C580R_DHA_treated and R539T_DHA_treated treatment conditions showed that module 7 was upregulated in the R539T _DHA_treated and the C580R_DHA_treated conditions.

**Figure 2:**
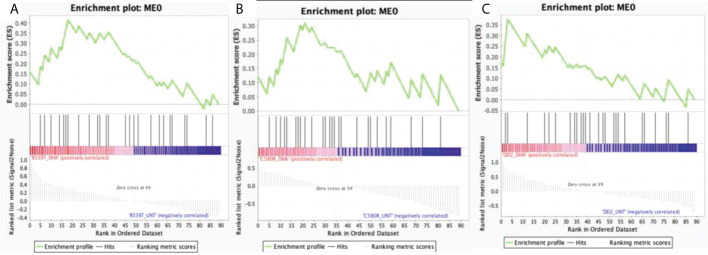
Enrichment score behavior showing the distribution of module 0 (ME0) in the input list of ranked genes for the three compared treatment conditions with FDR q-value < 0.25. Module 0 is the EVs module of interest comprising 693 genes with correlated expression with the PF3D7_1441800 gene (PfVps60) and PF3D7_0816200 (PfVps2) involved in EVs biogenesis in *P. falciparum*. The enrichment plots were generated by GSEA. Enrichment scores (ES) were calculated by strolling down the input list of ranked of genes, increasing a running-sum statistic when a gene is in ME0 and decreasing it when it is not. The magnitude of the increment depends on the correlation of the gene with the treatment condition. The ES is the maximum deviation from zero encountered in walking the list. The normalized enrichment score (NES) is calculated by normalizing the enrichment score for gene set size and represents the association of the modules with the treatment conditions compared. A positive NES indicates module enrichment and association with the first treatment condition. The higher the NES the stronger the association with the module. False discovery rate (FDR) was used to correct for multiple testing. Statistical significance was designated at an FDR q-value < 0.25. **(A)** R539T_DHA_treated versus R539T_untreated condition; ME0 was upregulated in the R539T_DHA_treated condition (NES = 1.18, FDR q-value = 0.24). **(B)** C580R_DHA_treated versus C580R_untreated; ME0 was upregulated in the C580R_DHA_treated condition (NES = 1.25, FDR q-value = 0.14). **(C)** DD2_DHA_treated versus DD2_untreated; ME0 was upregulated in the DD2_DHA_treated condition (NES = 1.18, FDR = 0.23). ME0, module 0; ES, enrichment score; NES, normalized enrichment score; FDR, False discovery rate; GSEA, gene set enrichment analysis.

Module 0 was significantly upregulated in the DHA-treated condition in both ARTr (C580R and R539T) and ART sensitive (DD2) parasite lines for the DHA-treated versus untreated condition. For the condition DHA treated versus DMSO-treated, module 0 was upregulated in the DMSO-treated condition for R539T and DD2 parasite lines, and in the DHA-treated condition for C580R line. Comparison of the DD2_DHA_treated condition with C580R_DHA_treated and R539T_DHA_treated treatment conditions showed that module 0 was upregulated in the C580R_DHA_treated condition and the DD2_DHA_treatment conditions respectively (**Table 2**, **Figure 2**; **Supplementary Data 1–8**).

**Figure 3:**
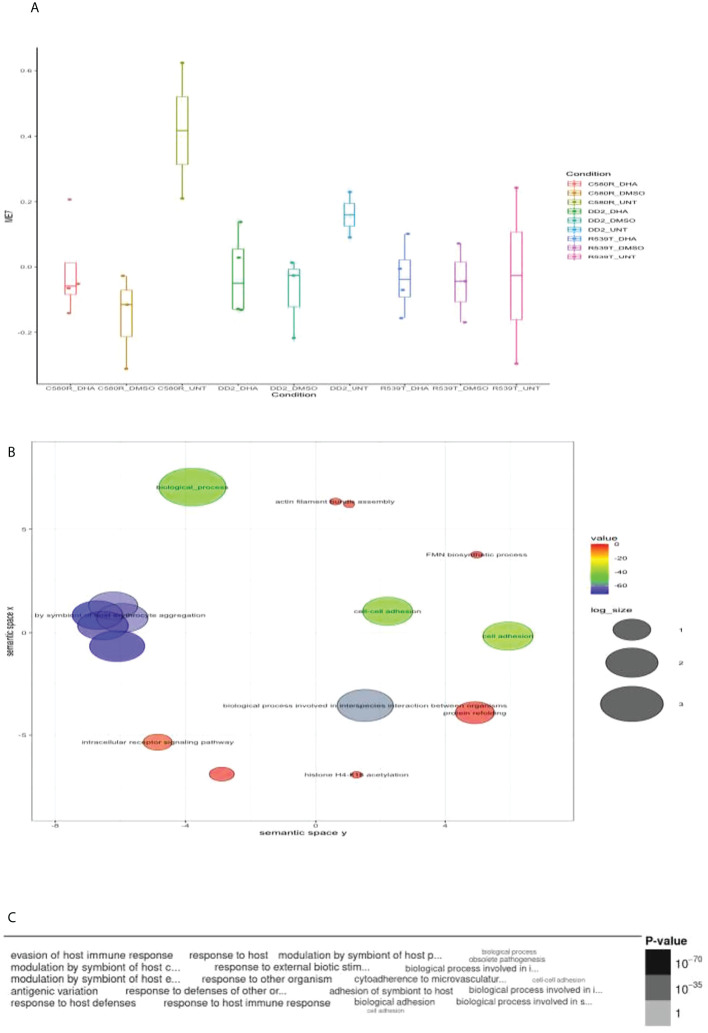
Properties of EVs module 7 (ME7). ME7 was uploaded to PlasmoDB(https://plasmodb.org/plasmo/app) and analyzed with the platform’s gene ontology (GO) pipeline. **(A)** Comparison of ME1 between the three treatment conditions pooled for all parasite lines (DD2, C580R and R539T). The Kruskal-Wallis test was used to determine the statistical significance of the difference in expression of ME7 among the conditions (p-value = 0.3606). **(B)** GO (biological processes) output for ME7 summarized using REVIGO (http://revigo.irb.hr) scatterplot visualization option. Each circle denotes the representative cluster of the GO biological process associated with the ARTr phenotype and is derived by multidimensional scaling and redundancy reduction of the GO sematic similarities. The size of the circles denote the frequency of the GO term in the *P. falciparum* GO database (updated in November 2021) with larger circles denoting more general terms. The colors (red to blue range) indicate the statistical significance of the association’s strength. “Modulation by symbiont of host erythrocyte aggregation” was one of the biological processes enriched **(C)** Word cloud summary of the GO biological processes associated with the ARTr phenotype. EVs, extracellular vesicles; ARTr, artemisinin resistant.

**Figure 4:**
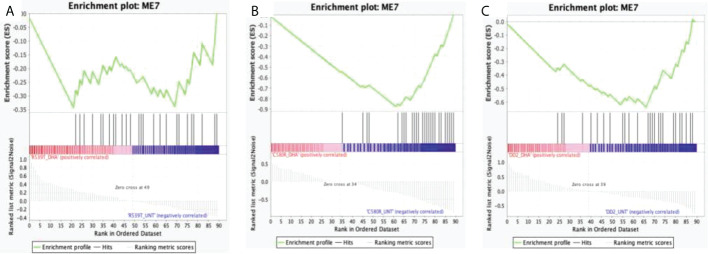
Enrichment score behavior showing the distribution of module 7 (ME0) in the input list of ranked genes for the three compared treatment conditions with FDR q-value < 0.25. Module 7 comprises 182 genes with correlated expression in *P. falciparum*. The enrichment plots were generated by GSEA. Enrichment scores (ES) were calculated by strolling down the input list of ranked of genes, increasing a running-sum statistic when a gene is in ME7 and decreasing it when it is not. The magnitude of the increment depends on the correlation of the gene with the treatment condition. The ES is the maximum deviation from zero encountered in walking the list. The normalized enrichment score (NES) is calculated by normalizing the enrichment score for gene set size and represents the association of the modules with the treatment conditions compared. A positive NES indicates module enrichment and association with the first treatment condition and a negative NES indicates module enrichment and association with the second treatment condition. The higher the NES the stronger the association with the module. False discovery rate (FDR) was used to correct for multiple testing. Statistical significance was designated at an FDR q-value < 0.25. **(A)** R539T_DHA_treated versus R539T_untreated condition; ME7 was upregulated in the R539T_untreated condition (NES = -1.27, FDR q-value = 0.15). **(B)** C580R_DHA_treated versus C580R_untreated; ME7 was upregulated in the C580R_DHA_treated condition (NES = -2.43, FDR q-value = 0.0). **(C)** DD2_DHA_treated versus DD2_untreated; ME7 was upregulated in the DD2_untreated condition (NES = -2.24, FDR = 0.0). ME7, module 7; ES, enrichment score; NES, normalized enrichment score; FDR, False discovery rate; GSEA, gene set enrichment analysis.

Module 7 comprised 182 genes (**Supplementary Table 2**) and showed differences in expression between treatment conditions (p-value = 0.3606) (**Figure 3**). GO analysis using the online PlasmoDB(https://plasmodb.org/plasmo/app, release 58) and REVIGO (http://revigo.irb.hr) platform (Supek et al., 2011) showed enrichment for biological processes related to modulation by symbiont of host erythrocyte aggregation (adjusted p-value = 1.17x10^-70^) (**Figure 3**; **Supplementary Table 8**). Module 7 was significantly upregulated in the untreated condition for both ARTr (C580R and R539T) and ART sensitive (DD2) parasite lines for the DHA-treated versus untreated condition. For the condition DHA treated versus DMSO-treated, module 7 was upregulated in the DMSO-treated condition for R539T and DD2 parasite lines, and in the DHA-treated condition for C580R line. Comparison of the DD2_DHA_treated condition with C580R_DHA_treated and R539T_DHA_treated treatment conditions showed that module 7 was upregulated in the R539T _DHA_treated and the C580R_DHA_treated conditions (**Table 2**, **Figure 4**; **Supplementary Data 9–16**).

### Analysis of differentially expressed genes reveals little insight into the role of EVs in *P. falciparum*

Next, we employed the traditional differentially expressed genes (DEGs) analysis pipeline to explore our research question of whether EVs play a role in the ARTr phenotype. DEGs were identified as genes with a log2 fold expression change > 2 (translating into more than a fourfold difference) and with a p-value < 0.05. For each DEGs list, gene ontology analysis for biological pathway enrichment was done using the online platforms PlasmoDB (https://plasmodb.org/plasmo/app, release 58) and REVIGO (Supek et al., 2011).

**Figure 5:**
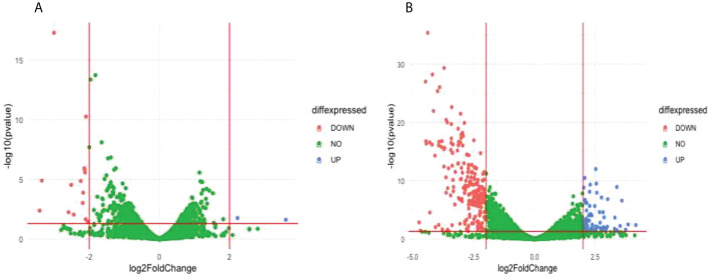
Differentially expressed genes (DEG) between compared conditions for the R539T parasite line. Scatter plots indicate the genes differentially expressed (log2 fold expression change > 2 and with a p-value < 0.05). Red vertical lines indicate absolute log2 fold change >2 and red horizontal line indicates p-value < 0.05. **(A)** Volcano plot for R539T_DMSO treated versus R539T_DHA treated; 15 genes were downregulated and 2 genes upregulated **(B)** Volcano plot for R539T_untreated compared with R539T_DHA; 70 genes upregulated, and 217 genes were downregulated.

Comparison of the treatment condition R539T_DMSO_treated versus R539T _DHA treated identified 2 upregulated genes and 15 downregulated genes (**Figure 5A**; **Supplementary Table 9**). Gene ontology enrichment analysis showed upregulation of molecular function, under DHA_treated condition, related to acyl-CoA binding, fatty-acyl-CoA binding, and fatty acid derivative binding; and downregulation of molecular function related to cholesterol binding (**Table 3**; **Supplementary Table 10**). Comparison of the treatment condition R539T_untreated versus R539T_DHA_treated identified 70 upregulated genes and 217 downregulated genes (**Figure 5B** and **Supplementary Table 11**). Gene ontology enrichment analysis showed downregulation of 18 biological processes, under DHA_treated condition, related to movement and entry into host environment, and downregulation of cellular components related to apical complex, extracellular vesicle, and extracellular membrane-bound organelle (**Table 3**; **Supplementary Table 12**).

**Table 3:** Summary of gene ontology enrichment analysis, of differentially expressed genes, identified a number of significantly enriched (bonferroni adjusted p-value < 0.05) biological processes, molecular function, and cellular components among the up/down-regulated genes of the compared treatment conditions.

Parasite Line	Treatment conditions compared	Number of genes (up/down-regulated)	Number upregulated in DHA_treated condition			Number down-regulated in DHA_treated condition		
			Biological process	Molecular function	Cellular component	Biological process	Molecular function	Cellular component
R539T	R539T_DHA_treated versus R539T_DMSO_treated	2/15	0	4	0	0	1	0
	R539T_DHA_treated versus R539T_untreated	70/217	0	0	1	18	7	29
C580R	C580R_DHA_treated versus C580R_DMSO_treated	1/21	0	0	0	3	0	2
	C580R_DHA_treated versus C580R_untreated	150/269	20	3	12	21	11	41
DD2	DD2_DHA_treated versus DD2_DMSO_treated	13/55	0	0	0	6	0	10
	DD2_DHA_treated versus DD2_untreated	247/403	0	0	2	14	11	30

**Figure 6:**
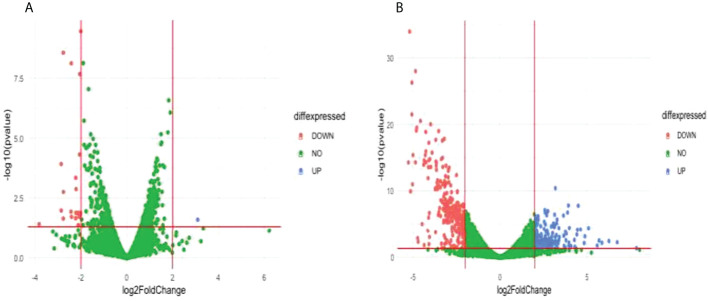
Differentially expressed genes (DEG) between compared conditions for the C580R parasite line. Scatter plots indicate the genes differentially expressed (log2 fold expression change > 2 and with a p-value < 0.05). Red vertical lines indicate absolute log2 fold change >2 and red horizontal line indicates p-value < 0.05. **(A)** Volcano plot for C580R_DMSO treated versus C580R_DHA treated. One gene was upregulated, and 21 genes were downregulated. **(B)** Volcano plot for C580R_untreated compared with C580R_DHA. 150 genes were upregulated, and 269 genes were downregulated genes.

Comparison of the treatment condition C580R_DMSO_treated versus C580R_DHA treated identified one upregulated gene and 21 downregulated genes (**Figure 6A**; **Supplementary Table 13**). Gene ontology enrichment analysis showed downregulation of molecular function, under DHA_treated condition, related to response to heat, temperature, and abiotic stimulus; and of cellular components related to the host (**Table 3**; **Supplementary Table 14**). Comparison of the treatment condition C580R_untreated versus C580R_DHA treated identified 150 upregulated genes and 269 downregulated genes (**Figure 6B**; **Supplementary Table 15**). Gene ontology enrichment analysis showed upregulation of biological processes, under DHA_treated condition, related to cytoadherence to the host microvasculature and antigenic variation; and downregulation of biological processes related to movement and entry into host environment. It also showed downregulation of cellular components related to apical complex, extracellular vesicle, and extracellular membrane-bound organelle (**Table 3**; **Supplementary Table 16**).

**Figure 7:**
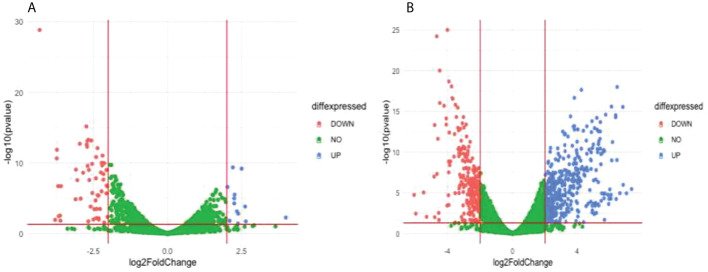
Differentially expressed genes (DEG) between compared conditions for the DD2 parasite line. Scatter plots indicate the genes differentially expressed (log2 fold expression change > 2 and with a p-value < 0.05). Red vertical lines indicate absolute log2 fold change >2 and red horizontal line indicates p-value < 0.05. **(A)** Volcano plot for DD2_DMSO treated versus DD2_DHA treated; 13 genes were upregulated, and 55 genes were downregulated **(B)** Volcano plot for DD2_untreated compared with DD2_DHA; 403 genes were upregulated and 247 downregulated genes.

Comparison of the treatment condition DD2_DMSO treated versus DD2_DHA treated identified 13 upregulated genes and 55 downregulated genes (**Figure 7A** and **Supplementary Table 17**). Gene ontology enrichment analysis showed downregulation, under DHA_treated condition, of biological processes related to chaperone cofactor-dependent protein refolding, ‘*de novo*’ posttranslational protein folding and chaperone-mediated protein folding (**Table 3**; **Supplementary Table 18**). Comparison of the treatment condition DD2_untreated versus DD2_DHA treated identified 403 upregulated genes and 247 downregulated genes (**Figure 7B** and **Supplementary Table 19**). Gene ontology enrichment analysis showed under downregulation, under DHA_treated condition, of biological processes related to entry and movement into host; and downregulation of cellular components related to apical complex, extracellular vesicle, and extracellular membrane-bound organelle (**Table 3**; **Supplementary Table 20**).

In summary, the largest number of DEGs were found in the DHA treatment condition, when compared to the untreated condition baseline, and was associated with an upregulation of 70 and 150 genes and down regulation of 217 and 269 genes in the ARTr parasite lines R539T and C580R respectively. DHA treatment, in the ART sensitive DD2 parasite line, was associated with 247 unregulated genes and 403 down regulated genes (**Table 3**; **Supplementary Tables 9–20**, **Supplementary Data 17**)

### A complex heterogeneous transcript profile characterizes *P. falciparum* ARTr parasites

**Figure 8:**
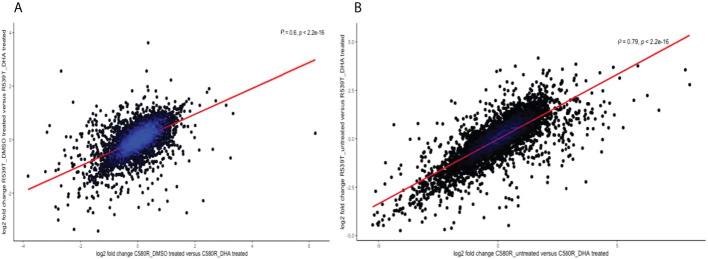
Scatter plots showing the strength of the correlation between the two ARTr biological replicates (C580R and R539T) for the compared conditions: **(A)** DMSO_treated versus DHA_treated and **(B)** untreated versus DHA_treated. The analysis explored the underlying heterogeneity in the gene expression profile between C580R and R539T and shows poor to moderate correlation in gene expression profile. The spearman rank correlation plot was constructed using the log2 fold change values between the compared conditions. ρ is the Spearman coefficient of correlation.

**Figure 9:**
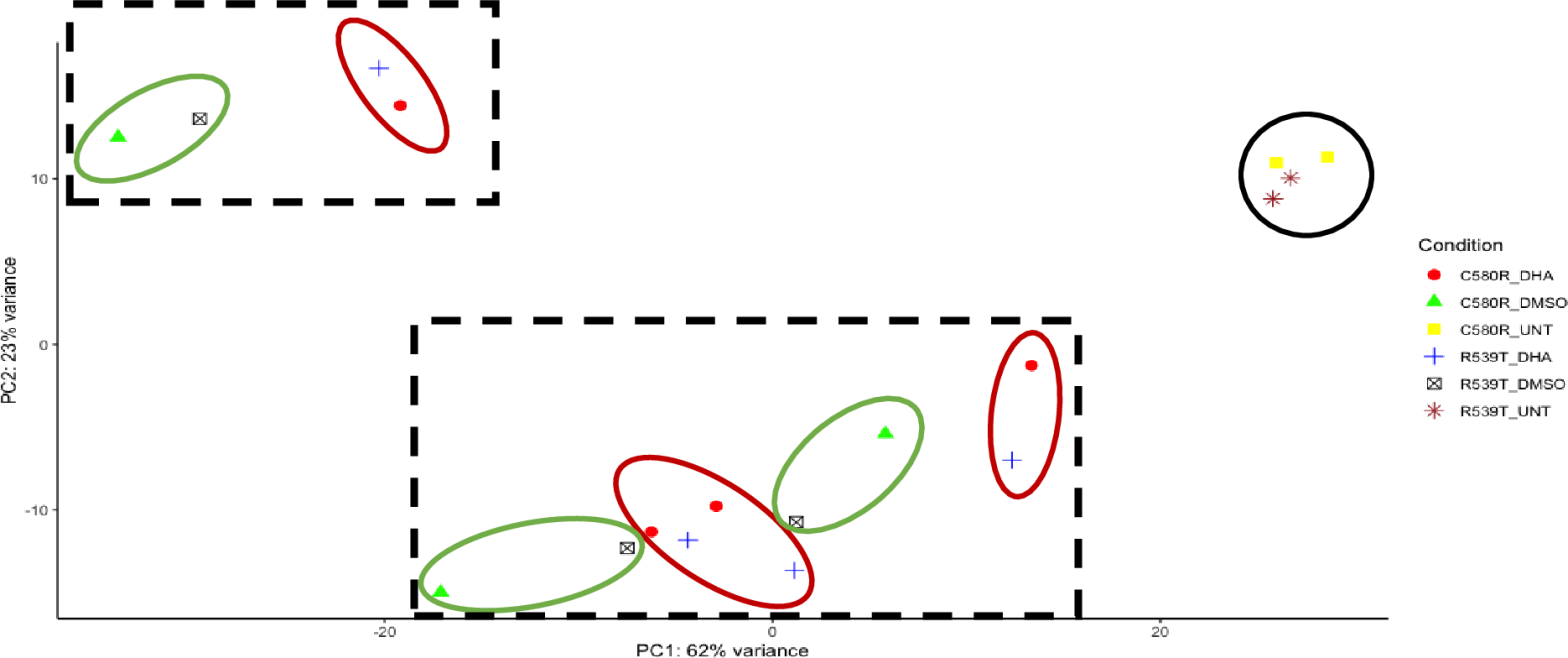
Principal component analysis (PCA) intimating at the transcriptional heterogeneity undergirding the ARTr phenotype. The dimensionality reducing technique PCA was used to explore the heterogeneity in gene expression profile under DMSO and DHA treatment conditions for the two ARTr parasite lines (C580R and R539T) used in this study. The black circle shows the untreated ARTr samples. The black dashed squares outlines the two clusters of DMSO and DHA-treated samples. In each square, the red circles show the different clusters for the C580R, and R539T DHA treated samples, and the green circles show the different clusters for the C580R and R539T DMSO treated samples.

Against the backdrop of the known heterogeneity in the ARTr phenotype (Siddiqui et al., 2021), we compared the gene expression profile between the two ARTr parasite lines (C580R and R539T) for the paired conditions of untreated versus DHA treated and DMSO treated versus DHA treated to determine whether the gene expression profiles were heterogeneous too. We expected that, given the ARTr phenotype heterogeneity, the gene expression profiles between the C580R and R539T parasite lines will not be well correlated. For the condition – DMSO versus DHA treated, between the ARTr parasite lines C580R and R539T the Spearman correlation coefficient was ρ = 0.6, p-value < 2.2 x10^-16^ (**Figure 8A**). For the condition – untreated versus DHA treated, between the ARTr parasite lines C580R and R539T the Spearman correlation coefficient was ρ = 0.79, p-value < 2.2 x10^-16^ (**Figure 8B**). This showed that the expression profile of ARTr parasite lines was poorly correlated and hinted at a transcriptionally heterogeneous ARTr parasite population. Additionally, this discordance in gene expression profile was further highlighted by principal component analysis (PCA) using- *vst* transformed data as input- for both ARTr parasite strains under DHA and DMSO treatment conditions, as only untreated ARTr samples clustered together (**Figure 9**). The presence of multiple clusters of ARTr parasite strains under DHA and DMSO treatment conditions adds to evidence for a complex heterogeneous transcript profile undergirding the known heterogeneity of the ARTr phenotype.

## Discussion

Against the backdrop of the need to deepen our understanding of the molecular mechanisms undergirding ARTr in *P. falciparum*, we sought to determine whether the gene expression profile of artemisinin-resistant parasites suggested the involvement of EVs. The mechanism of action of ART is known to involve the production of carbon-centred radicals, following reductive scission of the endoperoxide bridge, that promiscuously target proteome and nucleic acid and mediate cell-wide oxidative injury (Meshnick, 2002; O'neill et al., 2010; Gopalakrishnan and Kumar, 2015). This is unlike many antimalarial drugs that have well-defined drug targets and delineated mechanisms of action. This background accounts for the plethora of multiple molecular mechanisms reported to account for ARTr and is currently grouped under the organizing principles of increased tolerance to oxidative stress, reduced haemoglobin endocytosis and delay in the ring stage of the intra-erythrocytic developmental cycle (Siddiqui et al., 2021). Therefore, leveraging the power of RNA sequencing emerges as apt tool for interrogating the likely heterogeneous molecular mechanistic milieu of ARTr. This informed our choice to pivot the power of RNA sequencing to interrogate the role of EVs in *P. falciparum* ARTr. The novelty of our RNA sequencing experimental design, conduct and analysis lies in the nature of the research question posed and the association testing method employed using modular analysis and gene set enrichment analysis.

We posited a molecular mechanistic model of ART resistance involving EVs as vehicles responsible for the active removal of ART induced proteotoxic waste from the *P. falciparum*-infected erythrocyte (Tandoh et al., 2022). To test this model, we designed a targeted transcriptome exploratory analysis of two ARTr and one ART sensitive parasites (with three replicates each) under the three conditions: untreated, 0.1% DMSO treated and 700nM DHA treated for six hours. Therefore, the research question we explored was: does the gene expression profile of an ARTr parasite under ART pressure implicate a role for EVs? To explore this question, we designed a transcriptome-module phenotype association study (TMPAS). Our approach involved generating EVs modules of interest made of genes with correlated expression to known genes involved in the *P. falciparum* EVs biogenesis pathway. Next, we used these modules as *a priori* gene sets in a gene set enrichment analysis of the normalized and ranked gene expression data. We implicated EVs in ARTr phenotype because we found a module (module 0) associated with the ARTr phenotype (**Tables 1**, **2**; **Figures 1**, **2**).

An ever-present limitation for RNA sequencing analysis is the reliance on the known body of evidence (curated or computed) for GO analysis and distillation of meaning from the transcriptome data. If the research question borders on a biological phenomenon that little is known about, current GO analysis pipelines are poorly powered to detect any associations between the transcript data and the biological phenotype of interest. In this regard, very little insight was made by traditional GO analysis. As GO discovery is limited to what is already known, querying ontologies that lack data on EVs biogenesis expectedly revealed little insight (**Figures 4**-**6**, **Supplementary Tables 8-19**). We had anticipated this during our experimental design, hence our use of a TMPAS approach to distil meaning from the gene expression data.

First, we generated gene lists (EVs modules of interest) of known EVs biogenesis genes and genes with correlated expression (known interacting partners data was not found). By expanding the analytical power, a modular analysis approach increases the chance of distilling meaningful information from gene expression data. As it does not focus on one gene alone, a unit which by itself is unlikely to cause the biological phenomenon of interest to emerge, the modular analysis looks at the set of correlated genes that are likely to carry all the significant players associated or causal to the biological interest (Chaussabel and Baldwin, 2014; Bediako et al., 2019). The novelty in our analysis pipeline was to use our EVs modules of interest as our *a priori* gene list in our search for enrichment within the count data for the various paired conditions. We also analyzed the gene ontology signatures of our modules of EVs interest.

We report that out of the four EVs modules of interest generated, module 0 (ME0) was significantly associated with ARTr in *P. falciparum* (R539T_DHA_treated versus R539T_untreated: NES = 1.18, FDR q-value = 0.24; C580R_DHA_treated versus C580R_untreated: NES = 1.25, FDR q-value = 0.14) (**Table 2**; **Figure 2**). GO analysis of ME0 showed an enrichment for biological processes that included RNA processing, vesicle mediated transport and exocytosis (**Figure 1**; **Supplementary Table 3**). GO analysis of the other three EVs modules of interest (modules 1, 3 and 17) showed enrichment for biological processes related to movement in host environment, entry and exit into host (for module 1); translation and the proteasomal ubiquitinin system (module 3); and cell cycle, nuclear chromosome segregation processes and phosphatidylinositol-3-phosphate biosynthetic processes (module 17) (**Supplementary Tables 4–6**; **Supplementary Figures 10–12**).

The EVs gene of interest in module 0 was *PfVps60* implicated in EVs biogenesis by (Avalos-Padilla et al., 2021). It was the only gene that was successfully knocked out to yield a parasite line with significantly reduced EVs production. That the module containing such a gene was found to be upregulated under DHA pressure in ARTr suggests a role for EVs in ARTr. We opine that such a role for EVs in ARTr is likely hinged on the increased tolerance to oxidative stress arm of evidence explaining the molecular mechanisms of ARTr (Tandoh et al., 2022). Within this framework, we suggest that EVs may act as vehicles mediating the active removal of built-up and accumulating proteotoxic waste of ART action. Our data finds concordance with a recent multi-omics study that identified that ARTr involves a plethora of molecular mechanisms that included proteasome-mediated turnover, protein folding pathways and vesicular trafficking (Mok et al., 2021); and a transcriptome-phenotype association study that highlighted an association of the ARTr phenotype with proteotoxic stress, host cytoplasm remodelling and redox metabolism (Zhu et al., 2022). Further studies interrogating the members of this gene list are merited to further insights into the role of EVs in *P. falciparum* ARTr.

Our DEG analysis revealed that in the presence of DHA, approximately 400-600 genes were differentially expressed. These observations were similar to that reported earlier by (Mok et al., 2021). We observed that relatively smaller DEGs were found in the DHA treated condition when normalized to the DMSO treated condition (**Table 3**). This was not expected. We found non-statistically significant GO enrichment of biological processes related to the unfolded protein response (UPR) in C580R_DMSO treated versus C580R_DHA treated and DD2_DMSO treated versus DD2_DHA treated (**Supplementary Tables 14, 18**). We found this process downregulated under DHA treatment unlike earlier studies that reported upregulation of this biological process (Zhang et al., 2017; Rocamora et al., 2018; Mok et al., 2021; Zhu et al., 2022). Similarly downregulated and statistically not significant, were GO biological processes related to mitochondrial processes in R539T_DMSO treated versus R539T_DHA treated (**Supplementary Table 10**), unlike the findings of (Mok et al., 2021). Thus, our traditional DEG analysis did not recapitulate a statistically significant portrait in concordance with recent earlier findings (Mok et al., 2021; Zhu et al., 2022).

Finally, we report that the gene expression profile of ARTr *P. falciparum* parasites under ART exposure is complex and heterogeneous. This evidence for this heterogeneity in gene expression profile is seen in the low correlation plots between the two ARTr parasite strains (**Figure 8**). We also observed that PCA analysis of parasite treatment condition showed multiple clusters suggestive of a heterogeneous gene expression profile (**Figure 9**). Therefore we concluded that the transcript profile of ARTr parasites under DHA pressure is complex and likely harbours a heterogeneous cell population. This finding is expected as it is known that the expression of the ARTr phenotype is undergirded by a plethora of molecular mechanisms currently broadly fitted into two motifs viz reduced haemoglobin endocytosis into the digestive food vacuole and increased tolerance to oxidative stress (Siddiqui et al., 2021). Within this framework, it is plausible that genetically identical ARTr parasites might have significantly different expression profiles. Further transcriptomic and epigenetic studies investigating this observation will be needed. In this vein, future studies may employ a transcriptomics study at single-cell resolution to deconvolute this suspected transcriptional heterogeneity of ARTr parasites.

We recognize the following limitations in our study. Our experimental design used two to four replicates for each parasite line under each different condition. Increasing the replicate number could have increased the power of the study to determine the role of EVs in ARTr (Tarr et al., 2018). Secondly, we had to conduct the experiments in four different batches that may have introduced hard to control for technical variations. Although we tried to detect and control for this using the surrogate variables adjustment option in DaMiRseq, its effect on our data analysis pipeline cannot be fully discounted. Thirdly, our experimental design did not control for parasite age and asexual stage. These factors may have acted as confounders on some of our conclusions such as our intimation of heterogeneous gene expression profiles in the ARTr parasite lines. This limitation may explain the intermingling of the DMSO_treated and DHA_treated samples and why we saw a better correlation when we normalized to the untreated condition instead of the DMSO_treated condition (**Figures 8** and **9**). Finally, we noticed that most of the EVs modules of interest (three out of four) did not show association with the ARTr phenotype in the gene set enrichment analysis (**Table 1** and **2**, **Supplementary Data 1–16**). We opine that this does not conclusively rule out the alternative hypothesis that these EVs module of interest are correlated with ARTr. This is because, a critique of the frequentist as opposed to the Bayesian approach to hypothesis testing is the assumption of only one alternative to the null hypothesis. Thus, by failing to accept the null, it is implied that the alternative hypothesis is acceptable or correct. We recognize this is an overly simplistic heuristic approach to hypothesis testing by falsification. A rather conceivable alternative is that the alternative hypothesis are more than one and failing to accept the null hypothesis raises the question of which alternative hypothesis is acceptable. As our entire analytical framework was done within the enclave of this frequentist approach limitation, it will be interesting for future studies to explore a Bayesian approach to the data analysis hypothesis testing. Overall, further high-powered studies and replicates, correcting for these limitations, will be needed to interrogate our findings further.

## Conclusion

In conclusion, our data has shown that an EV module (module 0, containing the *PfVps60* gene) is associated with ART resistance and highlighted the complex and heterogeneous gene expression profile of ARTr parasites. This suggests that EVs biogenesis may play a critical role in mitigating proteotoxic injury in *P. falciparum*-infected erythrocytes. Therefore, further studies interrogating the implications of this study (EVs export hypothesis for ARTr in malaria parasites) are needed. Additionally, exploring further deconvolution of the heterogenous ARTr population of *P. falciparum* with single-cell transcriptomics is merited. We envisage that the findings reported here will aid future novel antimalarial drug discovery efforts.

## Data availability statement

The data presented in the study are deposited in the NCBI’s Gene Expression Omnibus (GEO) database repository with accession number GSE210644.

## Author contributions

KZT, MDW, NBQ and NOD conceptualized the research question. OCH generated the transgenic parasite lines used in this study. KZT did the *in vitro* wet-lab and computational data analysis and wrote the original manuscript. MDW, NBQ and NOD supervised the work and critically reviewed the manuscript. All authors read and approved the final manuscript.

## Funding

KZT and this work were supported by a PhD fellowship from Building a New Generation of Academics (BANGA Africa, University of Ghana, and Carnegie Corporation of New York); and WACCBIP-ACE PhD fellowship (WACCBIP + NCDs: Awandare). The funder was not involved in the study design, collection, analysis, interpretation of data, the writing of this article or the decision to submit it for publication. All authors declare no other competing interests.

## Acknowledgments

We acknowledge the University of Ghana for providing the high-performance computing resources (the ZUPUTO) used for some aspects of this work. We thank Single Cell Discoveries, especially Mauro Muraro, for their help with the free bulk RNA sequencing services, generation of the mapping quality metrics and count table.

## Conflict of interest

The authors declare that the research was conducted in the absence of any commercial or financial relationships that could be construed as a potential conflict of interest.

## Publisher’s note

All claims expressed in this article are solely those of the authors and do not necessarily represent those of their affiliated organizations, or those of the publisher, the editors and the reviewers. Any product that may be evaluated in this article, or claim that may be made by its manufacturer, is not guaranteed or endorsed by the publisher.
