# Supplementary material for: Transcriptome-module phenotype association study implicates extracellular vesicles biogenesis in Plasmodium falciparum artemisinin resistance
Source: Front Cell Infect Microbiol. 2022 Aug 19;12:886728. doi: 10.3389/fcimb.2022.886728 (PMC9437462; doi:10.3389/fcimb.2022.886728)
Supplement: Supplementary file 1 [file DataSheet_1.zip › Supplementary_files/Supplementary Figure_9.pdf]

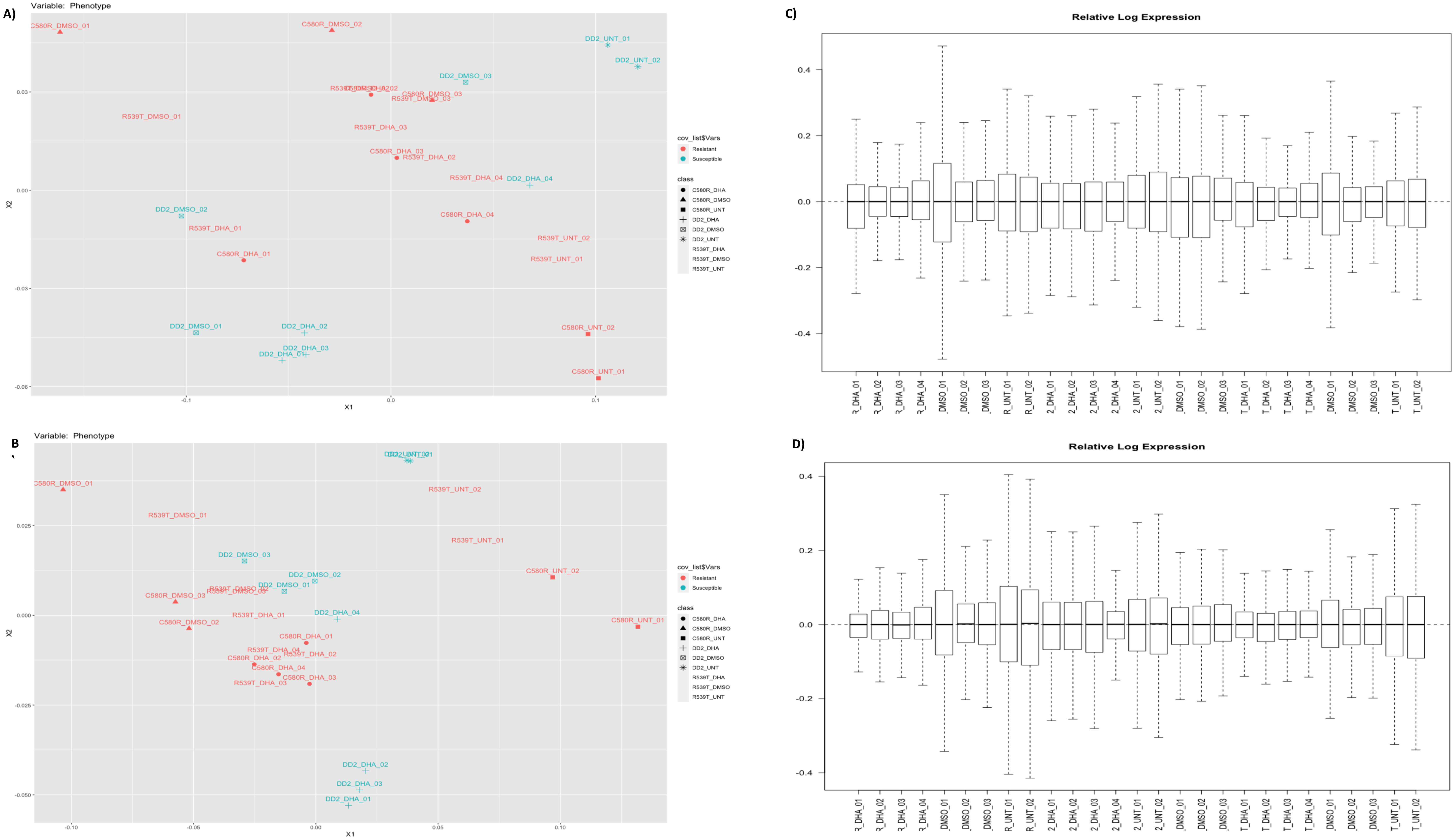

Supplementary Figure 9 | Interrogation of validity of RNA sequenced data. The R package DaMiRseq was used to filter, normalize and adjust the raw count data. Final adjustment for hidden sources of variation in the count data was done using surrogate variables (Svs). Top row indicate graphical summaries of count data structure before Svs adjustment and bottom row shows summaries following Svs adjustment. A) and B) Multidimensional scaling plot of count data showing parasite lines, technical replicates and treatment conditions. Following adjustment for Svs (B), parasite treatment conditions largely clustered together. C) and D) Following adjustment for Svs, relative log expression across samples was minimally affected.
