# Supplementary material for: Transcriptome-module phenotype association study implicates extracellular vesicles biogenesis in Plasmodium falciparum artemisinin resistance
Source: Front Cell Infect Microbiol. 2022 Aug 19;12:886728. doi: 10.3389/fcimb.2022.886728 (PMC9437462; doi:10.3389/fcimb.2022.886728)
Supplement: Supplementary file 1 [file DataSheet_1.zip › Supplementary_files/Supplementary_Table_1.pdf]

Supplementary Table 1 | Sample annotation file containing phenotype data for the twenty-seven samples used. For each of the 3 parasite lines, the following replicates were used: 4 replicates per parasite line for the 700nM DHA condition, 3 replicates per parasite line for the 0.1% DMSO control condition and 2 replicates per parasite line for the untreated condition. This table was also used to generate the phenotype data for the gene set enrichment analysis.

**sample**	**parasite**	**class/condition**	**sensitivity**	**batch**
C580R_DHA_01	C580R	DHA	Resistant	1
C580R_DHA_02	C580R	DHA	Resistant	2
C580R_DHA_03	C580R	DHA	Resistant	3
C580R_DHA_04	C580R	DHA	Resistant	4
C580R_DMSO_01	C580R	DMSO	Resistant	1
C580R_DMSO_02	C580R	DMSO	Resistant	2
C580R_DMSO_03	C580R	DMSO	Resistant	3
C580R_UNT_01	C580R	Untreated	Resistant	1
C580R_UNT_02	C580R	Untreated	Resistant	2
DD2_DHA_01	DD2	DHA	Susceptible	1
DD2_DHA_02	DD2	DHA	Susceptible	2
DD2_DHA_03	DD2	DHA	Susceptible	3
DD2_DHA_04	DD2	DHA	Susceptible	4
DD2_DMSO_01	DD2	DMSO	Susceptible	1
DD2_DMSO_02	DD2	DMSO	Susceptible	2
DD2_DMSO_03	DD2	DMSO	Susceptible	1
DD2_UNT_01	DD2	Untreated	Susceptible	2
DD2_UNT_02	DD2	Untreated	Susceptible	3
R539T_DHA_01	R539T	DHA	Resistant	1
R539T_DHA_02	R539T	DHA	Resistant	2
R539T_DHA_03	R539T	DHA	Resistant	3
R539T_DHA_04	R539T	DHA	Resistant	4
R539T_DMSO_01	R539T	DMSO	Resistant	1
R539T_DMSO_02	R539T	DMSO	Resistant	2
R539T_DMSO_03	R539T	DMSO	Resistant	3
R539T_UNT_01	R539T	Untreated	Resistant	1
R539T_UNT_02	R539T	Untreated	Resistant	1
