# Supplementary material for: Transcriptome-module phenotype association study implicates extracellular vesicles biogenesis in Plasmodium falciparum artemisinin resistance
Source: Front Cell Infect Microbiol. 2022 Aug 19;12:886728. doi: 10.3389/fcimb.2022.886728 (PMC9437462; doi:10.3389/fcimb.2022.886728)
Supplement: Supplementary file 1 [file DataSheet_1.zip › Supplementary_files/Supplementary_Data_1.pdf]

*Table: GSEA Results Summary*

| | |
|---|---|
| Dataset | Expression_dataset_dataset_collapsed_to_symbols.PhenotypeData.cls<br>#R539T_DHA_versus_R539T_UNT.PhenotypeData.cls<br>#R539T_DHA_versus_R539T_UNT_repos |
| Phenotype | PhenotypeData.cls#R539T_DHA_versus_R539T_UNT_repos |
| Upregulated in class | R539T_DHA |
| GeneSet | ME0 |
| Enrichment Score (ES) | 0.41410053 |
| Normalized Enrichment Score (NES) | 1.1830174 |
| Nominal p-value | 0.23866348 |
| FDR q-value | 0.23794872 |
| FWER p-Value | 0.373 |

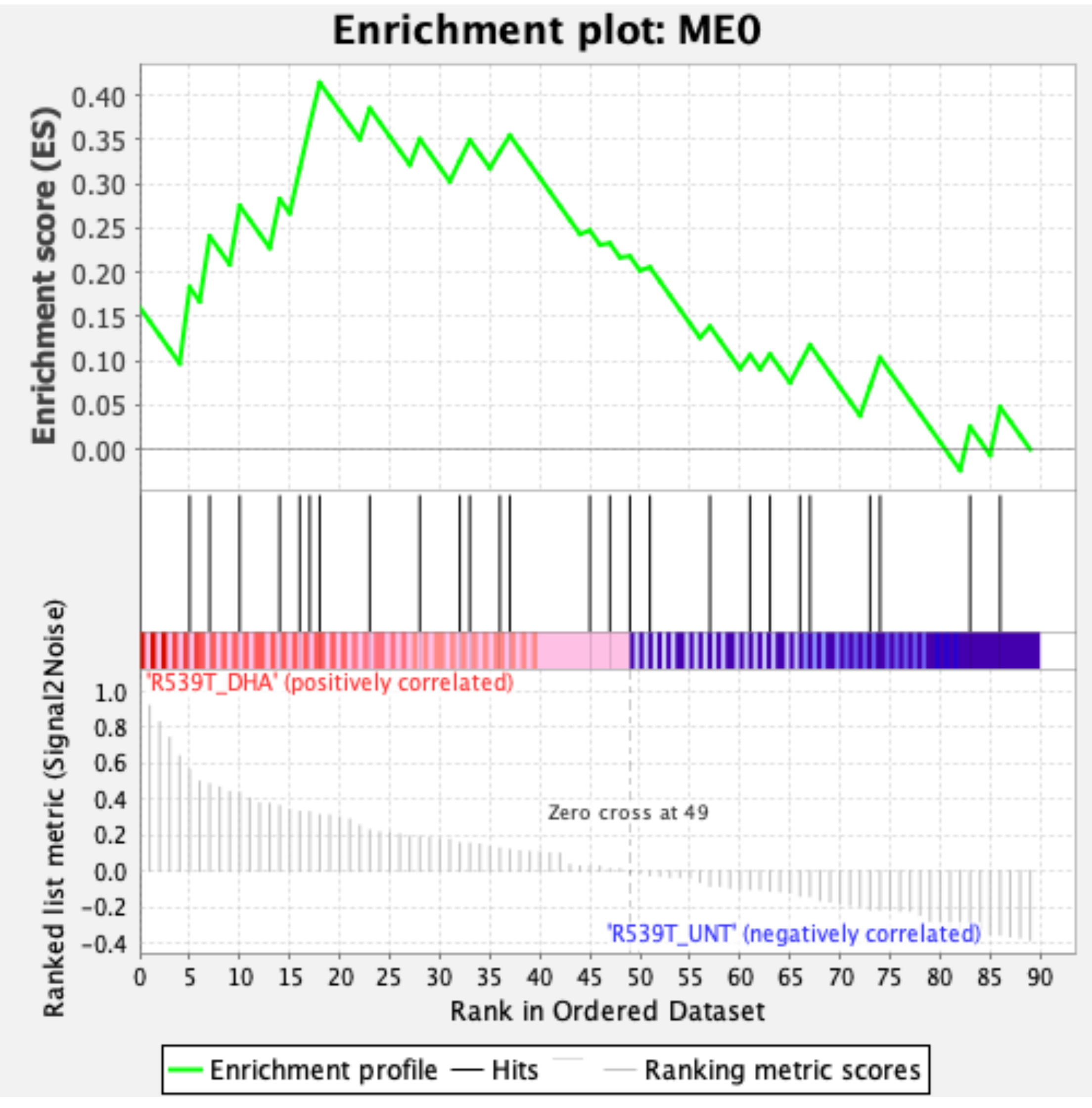


*Fig 1: Enrichment plot: ME0*
*Profile of the Running ES Score & Positions of GeneSet Members on the Rank Ordered List*

*Table: GSEA details* [plain text format]

| | SYMBOL | TITLE | RANK IN GENE LIST | RANK METRIC SCORE | RUNNING ES | CORE ENRICHMENT |
|---|---|---|---|---|---|---|
| 1 | PF3D7_0221500 | NA | 0 | 1.051 | 0.1606 | Yes |
| 2 | PF3D7_0400200 | NA | 5 | 0.562 | 0.1831 | Yes |
| 3 | PF3D7_0500600 | NA | 7 | 0.481 | 0.2408 | Yes |
| 4 | PF3D7_1477000 | NA | 10 | 0.433 | 0.2753 | Yes |
| 5 | PF3D7_1478700 | NA | 14 | 0.360 | 0.2827 | Yes |
| 6 | PF3D7_1000700 | NA | 16 | 0.328 | 0.3170 | Yes |
| 7 | PF3D7_0102100 | NA | 17 | 0.326 | 0.3668 | Yes |
| 8 | PF3D7_1372500 | NA | 18 | 0.309 | 0.4141 | Yes |
| 9 | PF3D7_1000800 | NA | 23 | 0.225 | 0.3851 | No |
| 10 | PF3D7_1219200 | NA | 28 | 0.189 | 0.3504 | No |
| 11 | PF3D7_0533000 | NA | 32 | 0.153 | 0.3262 | No |
| 12 | PF3D7_0601700 | NA | 33 | 0.151 | 0.3493 | No |
| 13 | PF3D7_0221100 | NA | 36 | 0.125 | 0.3366 | No |
| 14 | PF3D7_1334900 | NA | 37 | 0.117 | 0.3545 | No |
| 15 | PF3D7_0500700 | NA | 45 | 0.025 | 0.2471 | No |
| 16 | PF3D7_1129850 | NA | 47 | 0.010 | 0.2328 | No |
| 17 | PF3D7_1463100 | NA | 49 | -0.008 | 0.2181 | No |
| 18 | PF3D7_1220200 | NA | 51 | -0.022 | 0.2056 | No |
| 19 | PF3D7_0425250 | NA | 57 | -0.082 | 0.1388 | No |
| 20 | PF3D7_0425300 | NA | 61 | -0.100 | 0.1065 | No |
| 21 | PF3D7_1253900 | NA | 63 | -0.109 | 0.1073 | No |
| 22 | PF3D7_0424300 | NA | 66 | -0.137 | 0.0965 | No |
| 23 | PF3D7_1478200 | NA | 67 | -0.139 | 0.1177 | No |
| 24 | PF3D7_1478300 | NA | 73 | -0.213 | 0.0708 | No |
| 25 | PF3D7_0402700 | NA | 74 | -0.214 | 0.1035 | No |
| 26 | PF3D7_0532800 | NA | 83 | -0.319 | 0.0253 | No |
| 27 | PF3D7_1478500 | NA | 86 | -0.353 | 0.0476 | No |

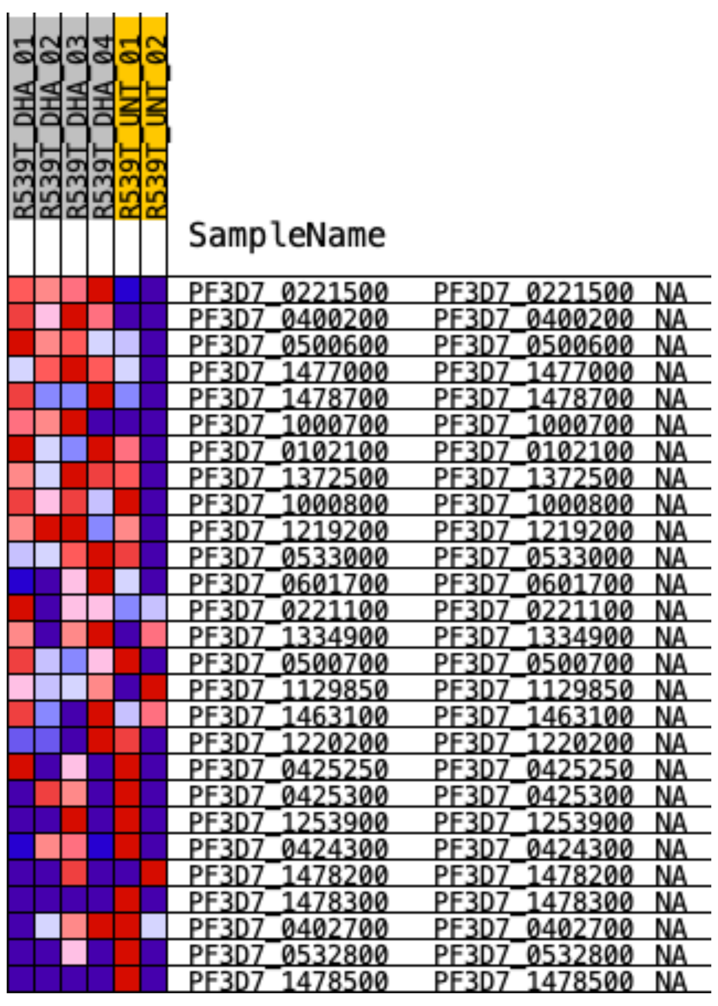


*Fig 2: ME0*
*Blue-Pink O' Gram in the Space of the Analyzed GeneSet*

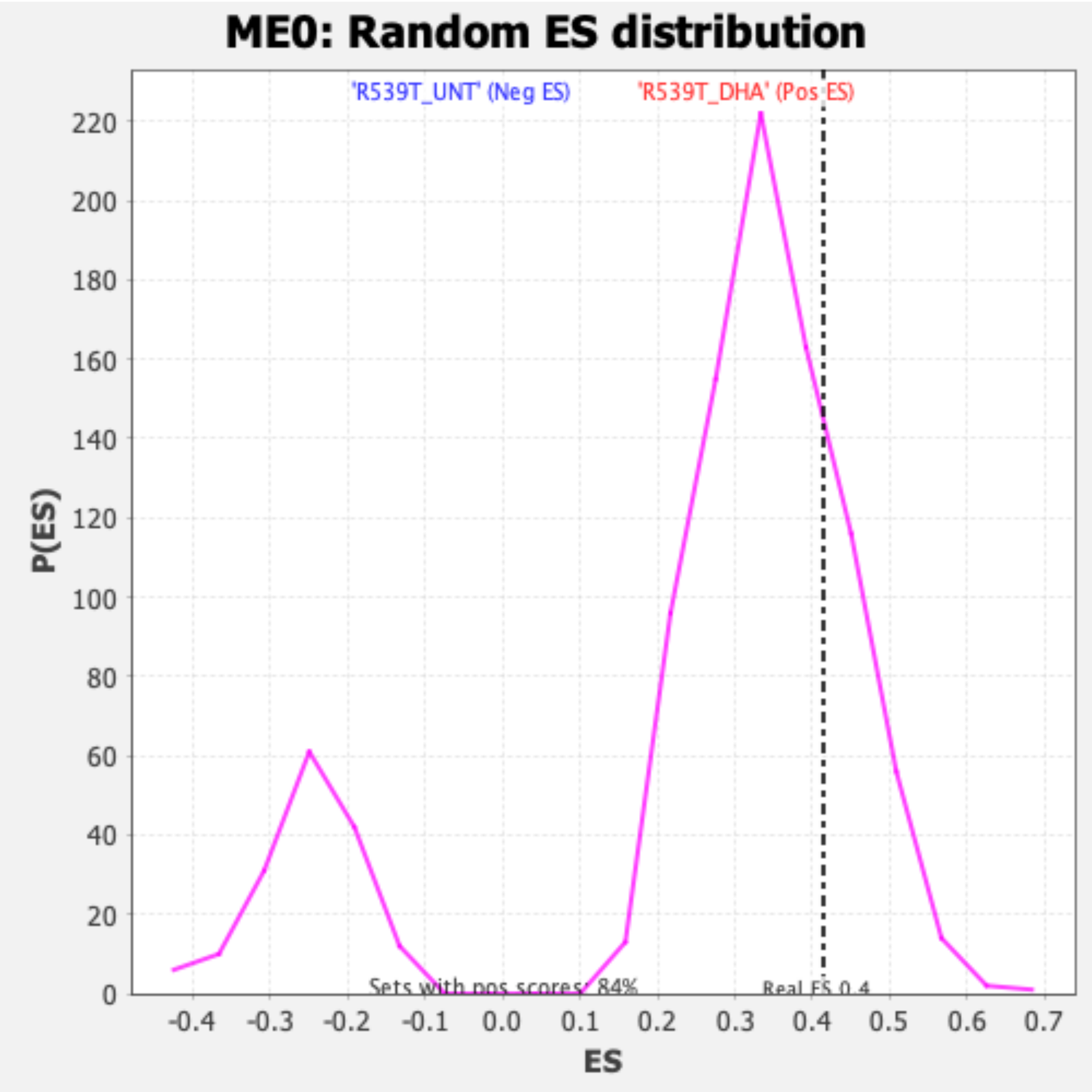


*Fig 3: ME0: Random ES distribution*
*Gene set null distribution of ES for ME0*
