# Supplementary material for: Transcriptome-module phenotype association study implicates extracellular vesicles biogenesis in Plasmodium falciparum artemisinin resistance
Source: Front Cell Infect Microbiol. 2022 Aug 19;12:886728. doi: 10.3389/fcimb.2022.886728 (PMC9437462; doi:10.3389/fcimb.2022.886728)
Supplement: Supplementary file 1 [file DataSheet_1.zip › Supplementary_files/Supplementary_Data_3.pdf]

Table: GSEA Results Summary

Dataset	Expression_dataset_dataset_collapsed_to_symbols.PhenotypeData.cls #R539T_DHA_versus_DD2_DHA.PhenotypeData.cls #R539T_DHA_versus_DD2_DHA_repos
Phenotype	PhenotypeData.cls#R539T_DHA_versus_DD2_DHA_repos
Upregulated in class	DD2_DHA
GeneSet	ME0
Enrichment Score (ES)	-0.41499826
Normalized Enrichment Score (NES)	-1.1220989
Nominal p-value	0.28860295
FDR q-value	0.28233033
FWER p-Value	0.275

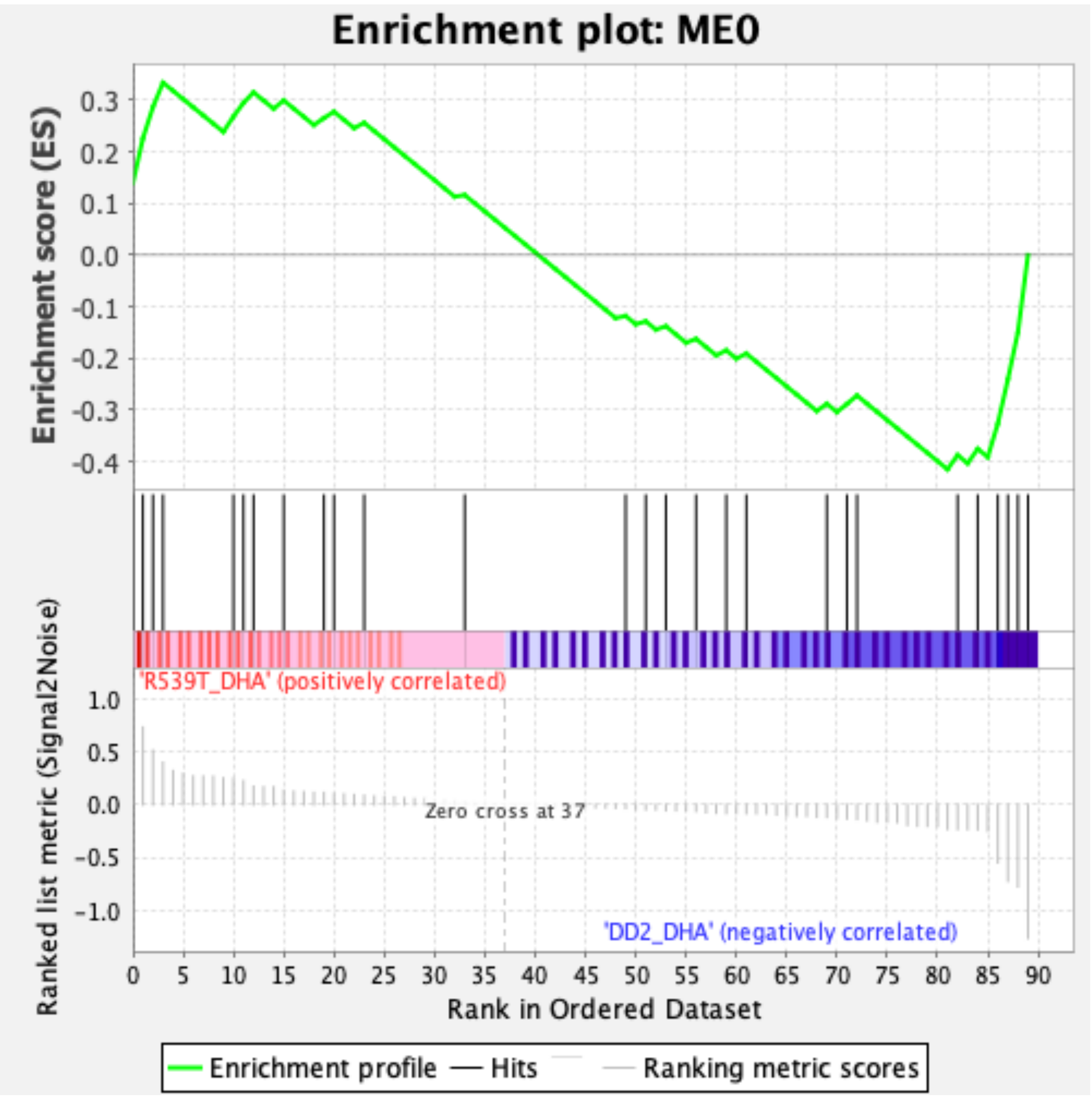

Fig 1: Enrichment plot: ME0
Profile of the Running ES Score & Positions of GeneSet Members on the Rank Ordered List

Table: GSEA details [plain text format]

	SYMBOL	TITLE	RANK IN GENE LIST	RANK METRIC SCORE	RUNNING ES	CORE ENRICHMENT
1	PF3D7_0221500	NA	0	1.179	0.1391	No
2	PF3D7_0102100	NA	1	0.734	0.2257	No
3	PF3D7_0221100	NA	2	0.514	0.2865	No
4	PF3D7_0500600	NA	3	0.401	0.3338	No
5	PF3D7_0400200	NA	10	0.249	0.2680	No
6	PF3D7_1463100	NA	11	0.226	0.2946	No
7	PF3D7_0425300	NA	12	0.173	0.3150	No
8	PF3D7_1220200	NA	15	0.136	0.2994	No
9	PF3D7_1334900	NA	19	0.114	0.2652	No
10	PF3D7_1478200	NA	20	0.104	0.2774	No
11	PF3D7_1129850	NA	23	0.087	0.2559	No
12	PF3D7_0425250	NA	33	0.025	0.1161	No
13	PF3D7_1219200	NA	49	-0.033	-0.1181	No
14	PF3D7_1478700	NA	51	-0.046	-0.1285	No
15	PF3D7_1372500	NA	53	-0.054	-0.1380	No
16	PF3D7_0500700	NA	56	-0.061	-0.1625	No
17	PF3D7_1478500	NA	59	-0.081	-0.1847	No
18	PF3D7_1253900	NA	61	-0.082	-0.1910	No
19	PF3D7_0533000	NA	69	-0.117	-0.2882	No
20	PF3D7_0601700	NA	71	-0.133	-0.2884	No
21	PF3D7_1478300	NA	72	-0.138	-0.2721	No
22	PF3D7_0532800	NA	82	-0.233	-0.3875	Yes
23	PF3D7_0402700	NA	84	-0.237	-0.3755	Yes
24	PF3D7_0424300	NA	86	-0.549	-0.3265	Yes
25	PF3D7_1000700	NA	87	-0.724	-0.2411	Yes
26	PF3D7_1477000	NA	88	-0.778	-0.1493	Yes
27	PF3D7_1000800	NA	89	-1.265	0.0000	Yes

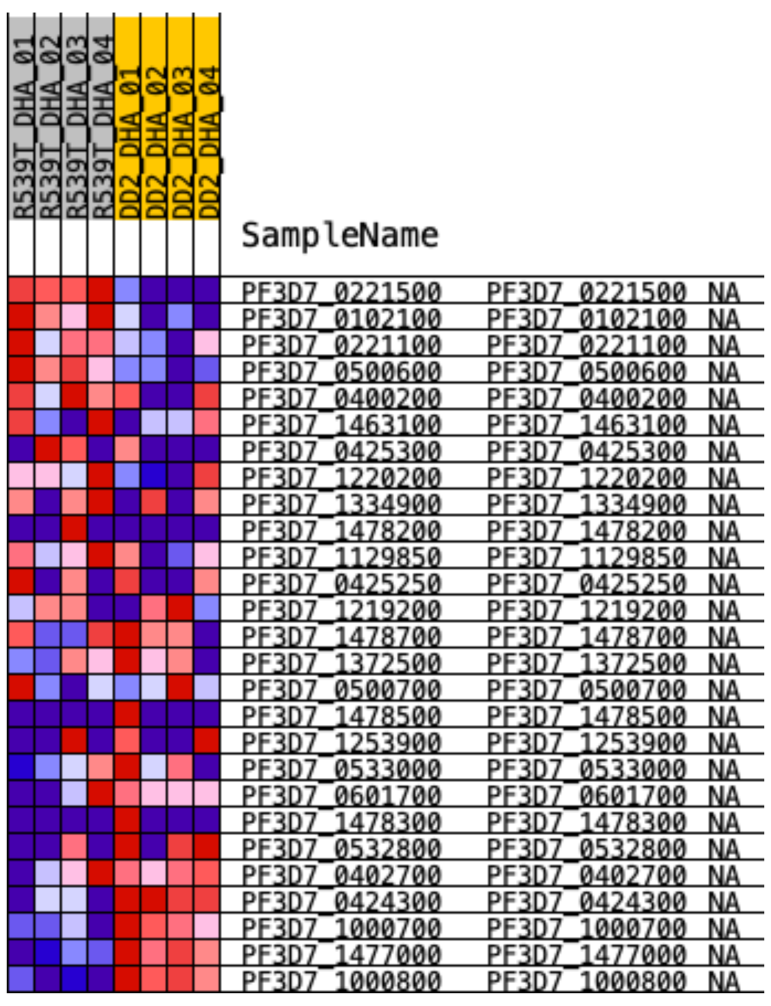

Fig 2: ME0
Blue-Pink O' Gram in the Space of the Analyzed GeneSet

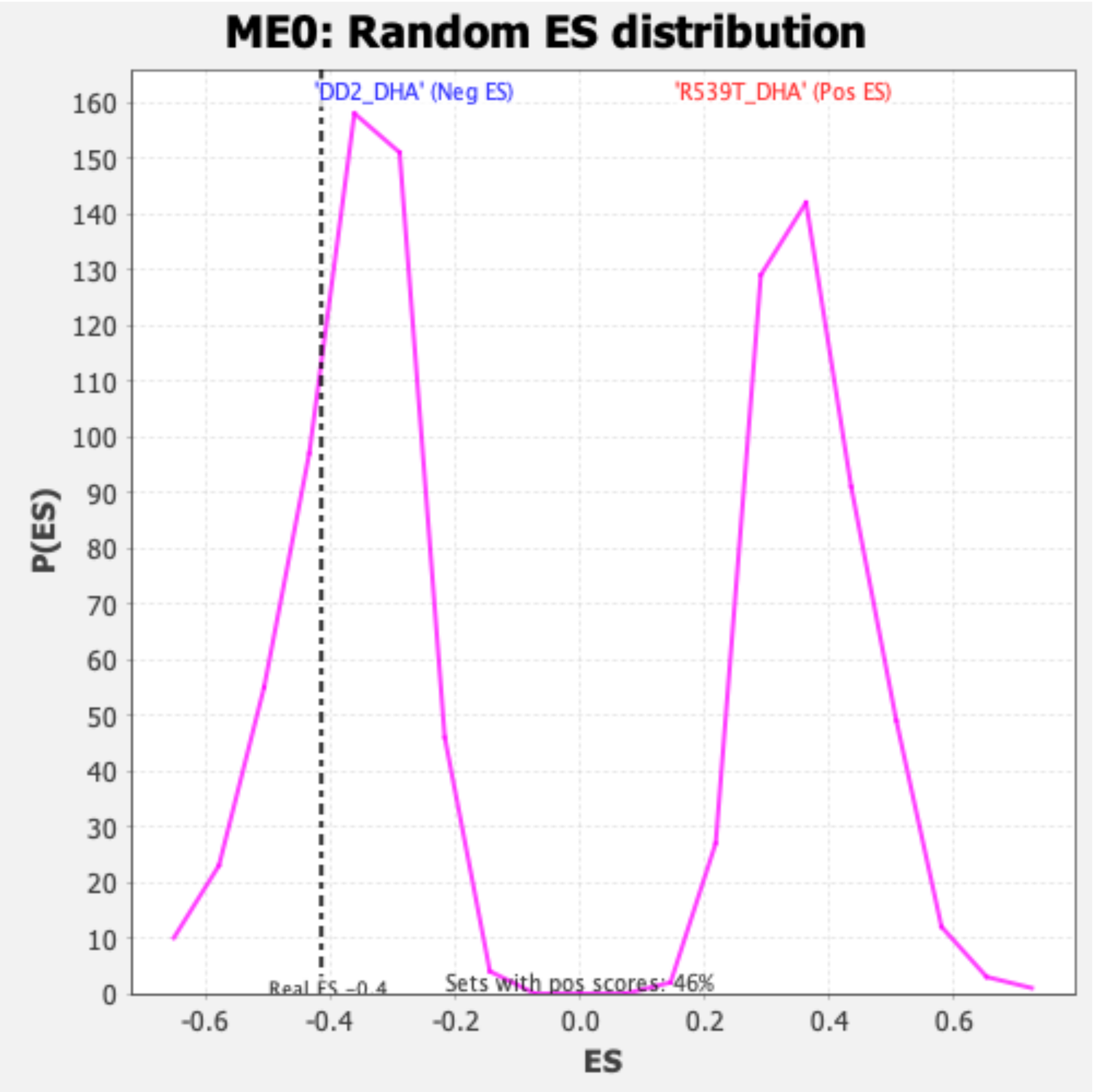

Fig 3: ME0: Random ES distribution
Gene set null distribution of ES for ME0
