# Supplementary material for: Transcriptome-module phenotype association study implicates extracellular vesicles biogenesis in Plasmodium falciparum artemisinin resistance
Source: Front Cell Infect Microbiol. 2022 Aug 19;12:886728. doi: 10.3389/fcimb.2022.886728 (PMC9437462; doi:10.3389/fcimb.2022.886728)
Supplement: Supplementary file 1 [file DataSheet_1.zip › Supplementary_files/Supplementary_Data_2.pdf]

Table: GSEA Results Summary

Dataset	Expression_dataset_dataset_collapsed_to_symbols.PhenotypeData.cls<br>#R539T_DHA_versus_R539T_DMSO.PhenotypeData.cls<br>#R539T_DHA_versus_R539T_DMSO_repos
Phenotype	PhenotypeData.cls#R539T_DHA_versus_R539T_DMSO_repos
Upregulated in class	R539T_DMSO
GeneSet	ME0
Enrichment Score (ES)	-0.20600075
Normalized Enrichment Score (NES)	-0.78877187
Nominal p-value	0.85714287
FDR q-value	0.859322
FWER p-Value	0.255

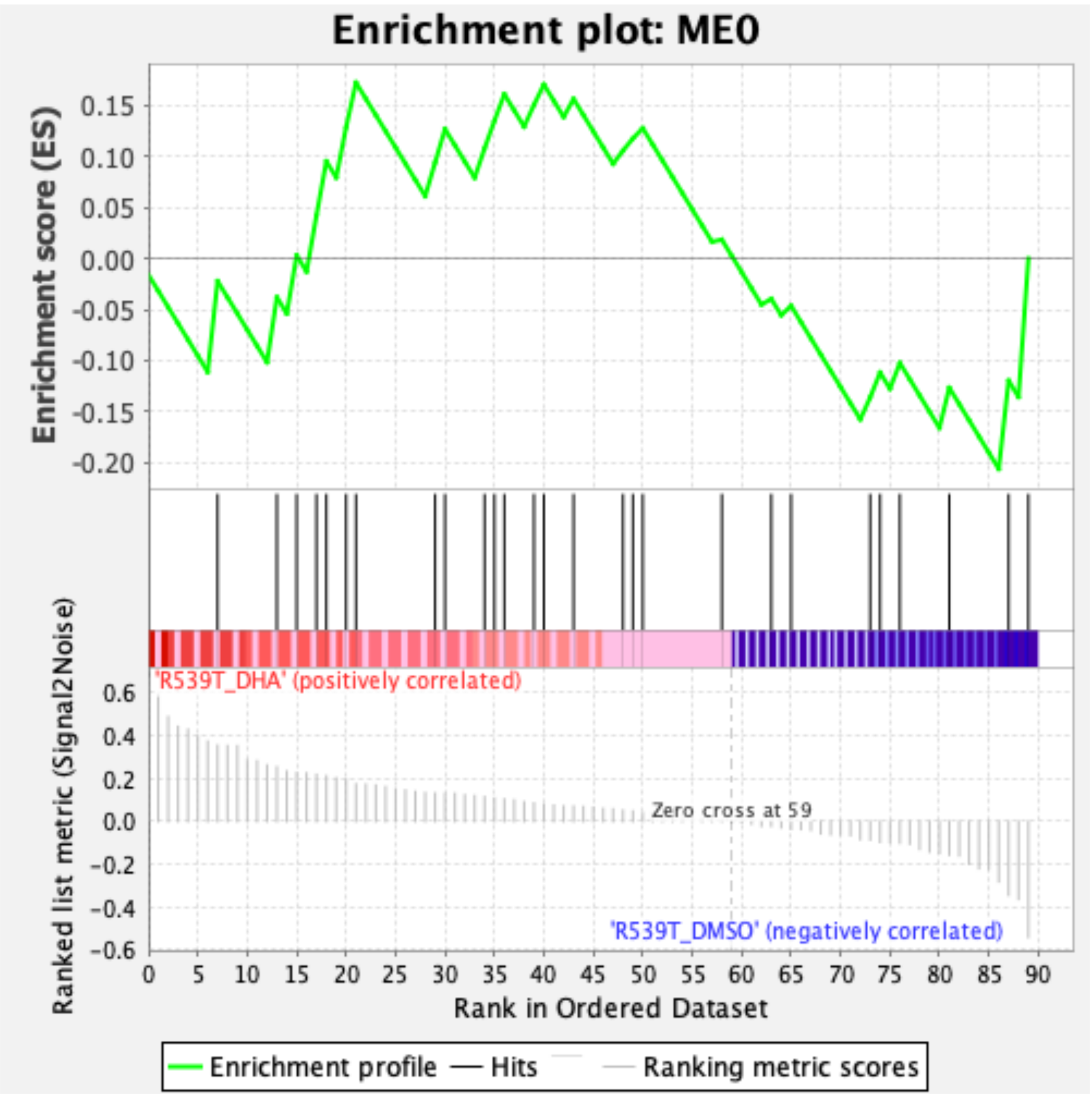

Fig 1: Enrichment plot: ME0
Profile of the Running ES Score & Positions of GeneSet Members on the Rank Ordered List

Table: GSEA details [plain text format]

	SYMBOL	TITLE	RANK IN GENE LIST	RANK METRIC SCORE	RUNNING ES	CORE ENRICHMENT
1	PF3D7_0221500	NA	7	0.355	-0.0222	Yes
2	PF3D7_0425300	NA	13	0.254	-0.0380	Yes
3	PF3D7_0500600	NA	15	0.227	0.0030	Yes
4	PF3D7_0500700	NA	17	0.219	0.0420	Yes
5	PF3D7_0425250	NA	18	0.212	0.0951	Yes
6	PF3D7_0533000	NA	20	0.197	0.1285	Yes
7	PF3D7_1478700	NA	21	0.174	0.1721	Yes
8	PF3D7_1129850	NA	29	0.131	0.0939	No
9	PF3D7_1477000	NA	30	0.131	0.1266	No
10	PF3D7_1000800	NA	34	0.114	0.1077	No
11	PF3D7_0400200	NA	35	0.108	0.1348	No
12	PF3D7_1253900	NA	36	0.104	0.1608	No
13	PF3D7_1372500	NA	39	0.085	0.1504	No
14	PF3D7_1463100	NA	40	0.079	0.1703	No
15	PF3D7_0601700	NA	43	0.070	0.1562	No
16	PF3D7_1220200	NA	48	0.051	0.1055	No
17	PF3D7_1334900	NA	49	0.047	0.1174	No
18	PF3D7_0221100	NA	50	0.040	0.1275	No
19	PF3D7_1478200	NA	58	0.008	0.0184	No
20	PF3D7_0532800	NA	63	-0.022	-0.0397	No
21	PF3D7_0102100	NA	65	-0.036	-0.0464	No
22	PF3D7_0424300	NA	73	-0.086	-0.1360	No
23	PF3D7_1219200	NA	74	-0.097	-0.1117	No
24	PF3D7_1000700	NA	76	-0.101	-0.1024	No
25	PF3D7_0402700	NA	81	-0.157	-0.1266	No
26	PF3D7_1478300	NA	87	-0.345	-0.1197	No
27	PF3D7_1478500	NA	89	-0.541	0.0000	No

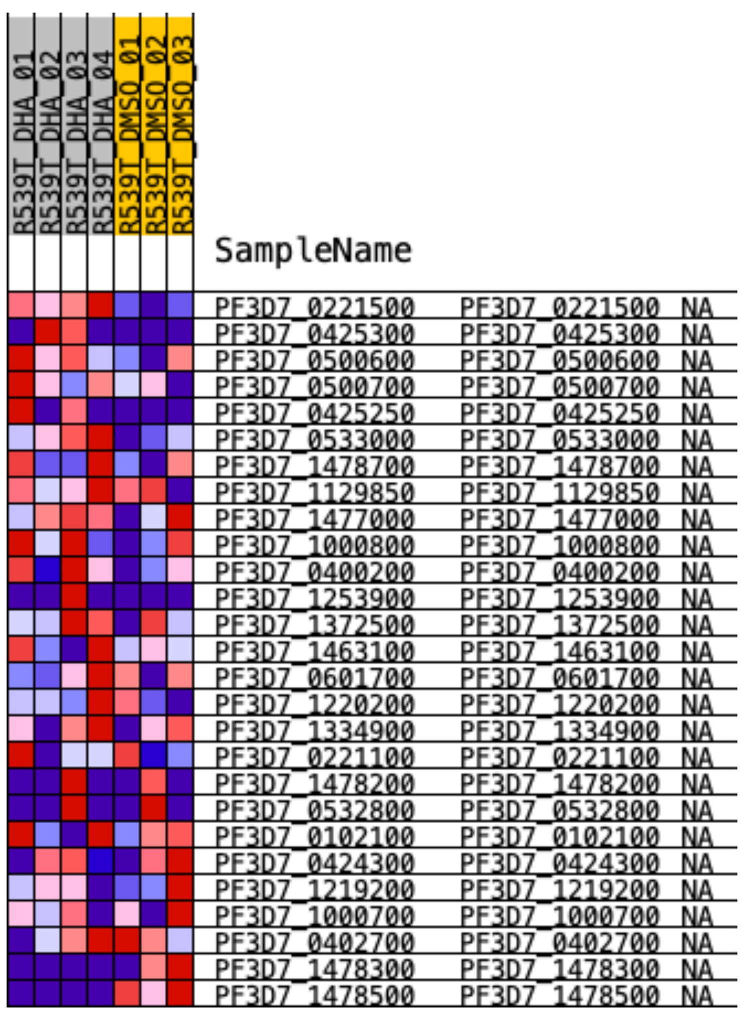

Fig 2: ME0
Blue-Pink O' Gram in the Space of the Analyzed GeneSet

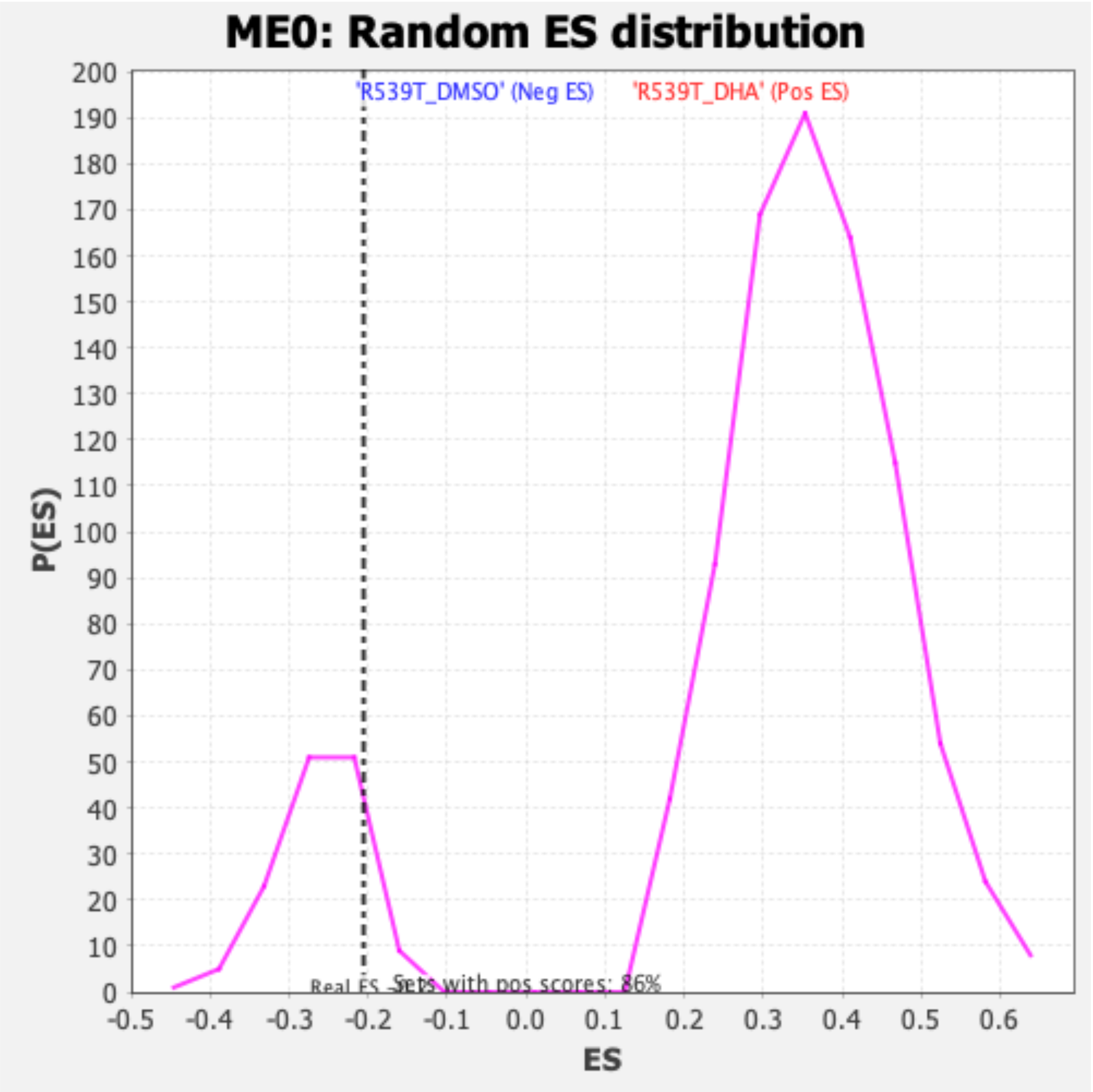

Fig 3: ME0: Random ES distribution
Gene set null distribution of ES for ME0
