# Supplementary material for: Transcriptome-module phenotype association study implicates extracellular vesicles biogenesis in Plasmodium falciparum artemisinin resistance
Source: Front Cell Infect Microbiol. 2022 Aug 19;12:886728. doi: 10.3389/fcimb.2022.886728 (PMC9437462; doi:10.3389/fcimb.2022.886728)
Supplement: Supplementary file 1 [file DataSheet_1.zip › Supplementary_files/Supplementary_Data_6.pdf]

Table: GSEA Results Summary

Dataset	Expression_dataset_dataset_collapsed_to_symbols.PhenotypeData.cls<br>#C580R_DHA_versus_DD2_DHA.PhenotypeData.cls<br>#C580R_DHA_versus_DD2_DHA_repos
Phenotype	PhenotypeData.cls#C580R_DHA_versus_DD2_DHA_repos
Upregulated in class	C580R_DHA
GeneSet	ME0
Enrichment Score (ES)	0.40779817
Normalized Enrichment Score (NES)	1.0806667
Nominal p-value	0.34814814
FDR q-value	0.35456595
FWER p-Value	0.416

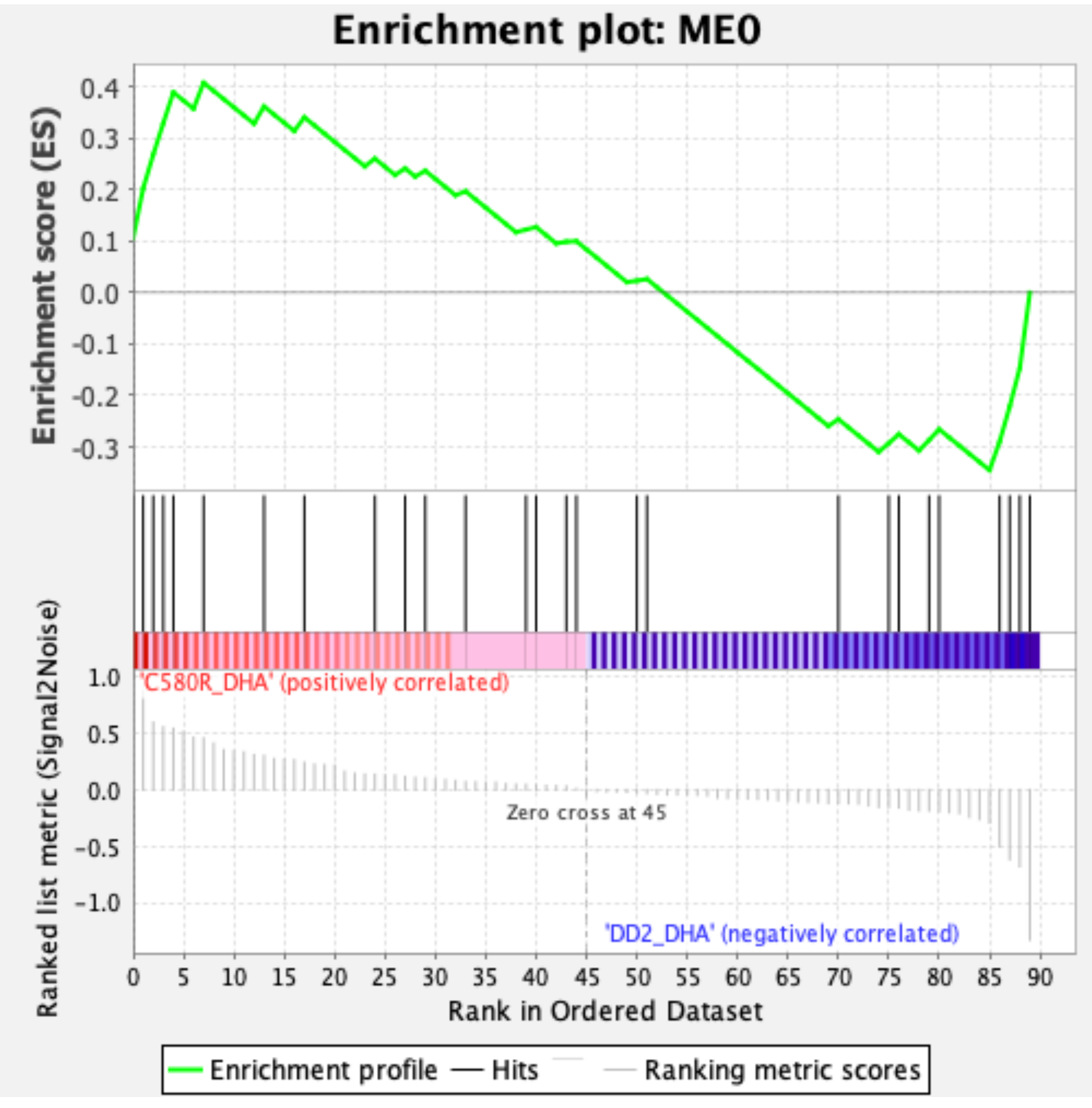

Fig 1: Enrichment plot: ME0
Profile of the Running ES Score & Positions of GeneSet Members on the Rank Ordered List

Table: GSEA details [plain text format]

	SYMBOL	TITLE	RANK IN GENE LIST	RANK METRIC SCORE	RUNNING ES	CORE ENRICHMENT
1	PF3D7_1372500	NA	0	0.958	0.1057	Yes
2	PF3D7_0533000	NA	1	0.875	0.2023	Yes
3	PF3D7_0425250	NA	2	0.596	0.2681	Yes
4	PF3D7_0500600	NA	3	0.556	0.3294	Yes
5	PF3D7_0221100	NA	4	0.542	0.3892	Yes
6	PF3D7_0221500	NA	7	0.456	0.4078	Yes
7	PF3D7_0425300	NA	13	0.302	0.3617	No
8	PF3D7_1478500	NA	17	0.241	0.3407	No
9	PF3D7_1463100	NA	24	0.135	0.2604	No
10	PF3D7_1220200	NA	27	0.115	0.2414	No
11	PF3D7_1478200	NA	29	0.103	0.2368	No
12	PF3D7_1219200	NA	33	0.069	0.1968	No
13	PF3D7_0500700	NA	39	0.045	0.1224	No
14	PF3D7_0102100	NA	40	0.044	0.1273	No
15	PF3D7_0400200	NA	43	0.030	0.0989	No
16	PF3D7_1334900	NA	44	0.008	0.0998	No
17	PF3D7_1478700	NA	50	-0.024	0.0230	No
18	PF3D7_1478300	NA	51	-0.030	0.0263	No
19	PF3D7_0532800	NA	70	-0.119	-0.2462	No
20	PF3D7_0402700	NA	75	-0.154	-0.2927	No
21	PF3D7_0601700	NA	76	-0.158	-0.2753	No
22	PF3D7_1253900	NA	79	-0.185	-0.2866	No
23	PF3D7_1129850	NA	80	-0.189	-0.2658	No
24	PF3D7_0424300	NA	86	-0.503	-0.2897	No
25	PF3D7_1000700	NA	87	-0.617	-0.2216	No
26	PF3D7_1477000	NA	88	-0.678	-0.1467	No
27	PF3D7_1000800	NA	89	-1.329	0.0000	No

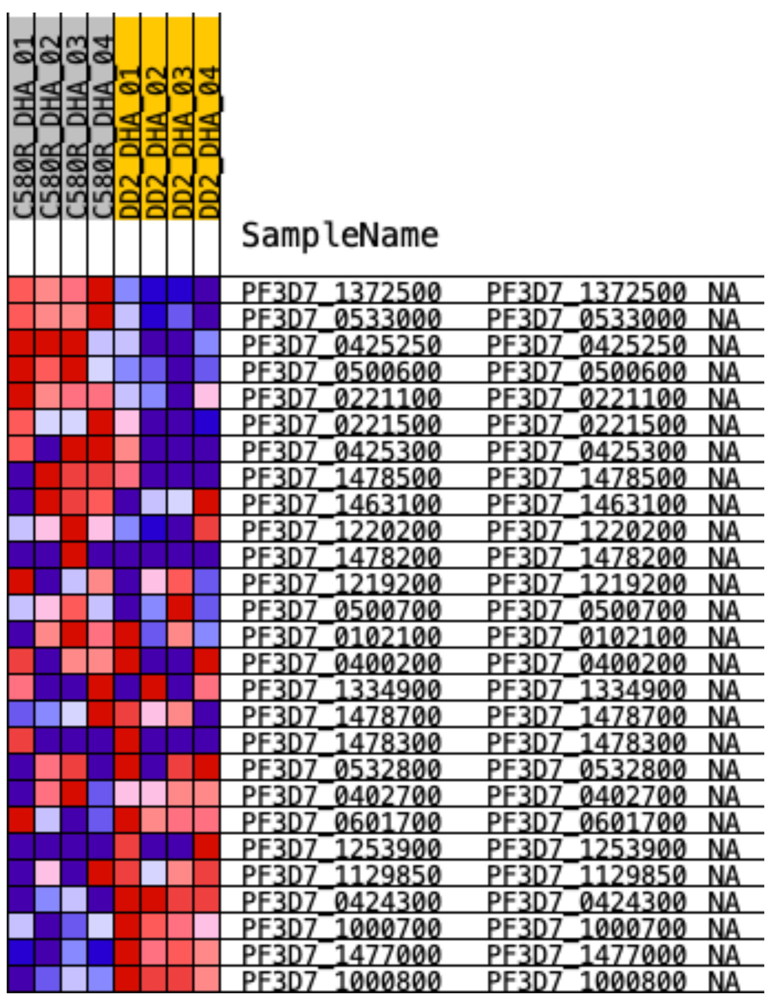

Fig 2: ME0
Blue-Pink O' Gram in the Space of the Analyzed GeneSet

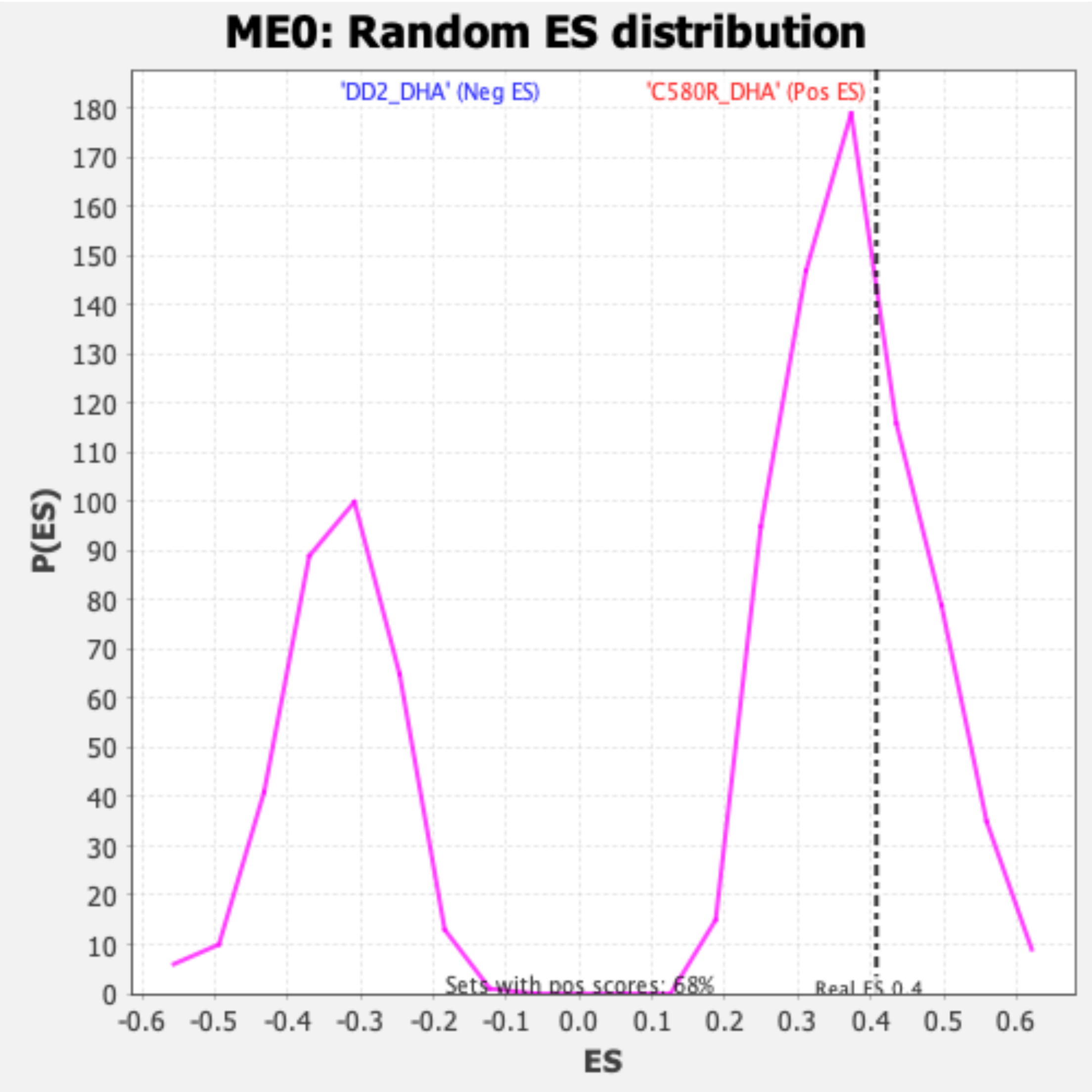

Fig 3: ME0: Random ES distribution
Gene set null distribution of ES for ME0
