# Supplementary material for: Transcriptome-module phenotype association study implicates extracellular vesicles biogenesis in Plasmodium falciparum artemisinin resistance
Source: Front Cell Infect Microbiol. 2022 Aug 19;12:886728. doi: 10.3389/fcimb.2022.886728 (PMC9437462; doi:10.3389/fcimb.2022.886728)
Supplement: Supplementary file 1 [file DataSheet_1.zip › Supplementary_files/Supplementary_Data_7.pdf]

Table: GSEA Results Summary

Dataset	Expression_dataset_dataset_collapsed_to_symbols.PhenotypeData.cls #DD2_DHA_versus_DD2_UNT.PhenotypeData.cls #DD2_DHA_versus_DD2_UNT_repos
Phenotype	PhenotypeData.cls#DD2_DHA_versus_DD2_UNT_repos
Upregulated in class	DD2_DHA
GeneSet	ME0
Enrichment Score (ES)	0.37629843
Normalized Enrichment Score (NES)	1.1802711
Nominal p-value	0.22782874
FDR q-value	0.22940503
FWER p-Value	0.282

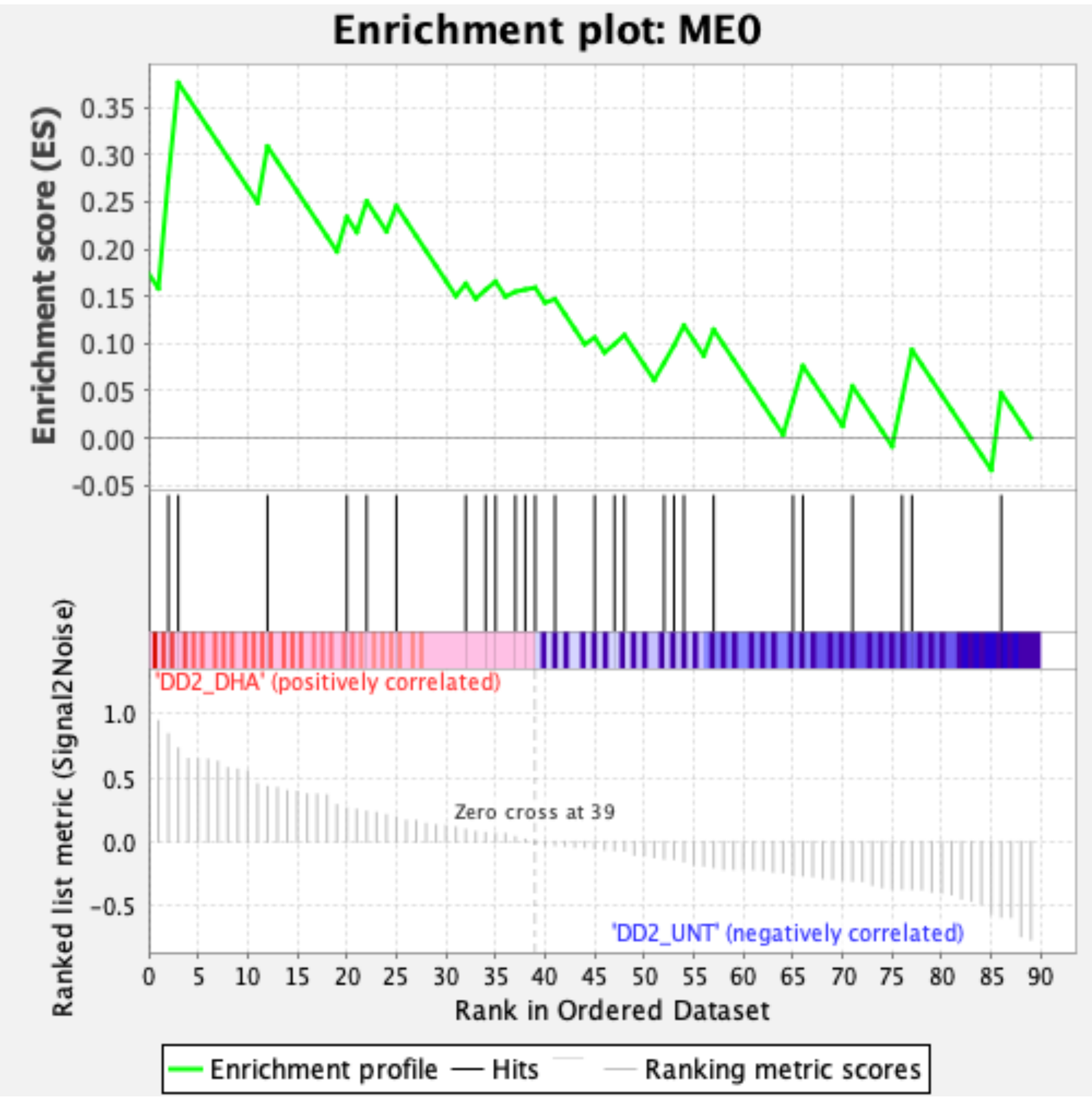

Fig 1: Enrichment plot: ME0
Profile of the Running ES Score & Positions of GeneSet Members on the Rank Ordered List

Table: GSEA details [plain text format]

	SYMBOL	TITLE	RANK IN GENE LIST	RANK METRIC SCORE	RUNNING ES	CORE ENRICHMENT
1	PF3D7_1000800	NA	0	1.254	0.1743	Yes
2	PF3D7_1477000	NA	2	0.840	0.2752	Yes
3	PF3D7_1000700	NA	3	0.728	0.3763	Yes
4	PF3D7_0424300	NA	12	0.428	0.3088	No
5	PF3D7_0402700	NA	20	0.262	0.2340	No
6	PF3D7_1372500	NA	22	0.234	0.2507	No
7	PF3D7_1478700	NA	25	0.192	0.2456	No
8	PF3D7_1334900	NA	32	0.093	0.1634	No
9	PF3D7_0601700	NA	34	0.069	0.1570	No
10	PF3D7_0500600	NA	35	0.062	0.1657	No
11	PF3D7_1220200	NA	37	0.037	0.1549	No
12	PF3D7_0500700	NA	38	0.017	0.1572	No
13	PF3D7_1478300	NA	39	-0.014	0.1592	No
14	PF3D7_1253900	NA	41	-0.026	0.1470	No
15	PF3D7_1463100	NA	45	-0.050	0.1064	No
16	PF3D7_0221500	NA	47	-0.064	0.0994	No
17	PF3D7_0533000	NA	48	-0.069	0.1090	No
18	PF3D7_1478500	NA	52	-0.131	0.0796	No
19	PF3D7_1219200	NA	53	-0.133	0.0980	No
20	PF3D7_1478200	NA	54	-0.152	0.1191	No
21	PF3D7_0102100	NA	57	-0.197	0.1147	No
22	PF3D7_1129850	NA	65	-0.261	0.0398	No
23	PF3D7_0400200	NA	66	-0.262	0.0762	No
24	PF3D7_0425250	NA	71	-0.302	0.0548	No
25	PF3D7_0532800	NA	76	-0.366	0.0421	No
26	PF3D7_0425300	NA	77	-0.368	0.0932	No
27	PF3D7_0221100	NA	86	-0.586	0.0476	No

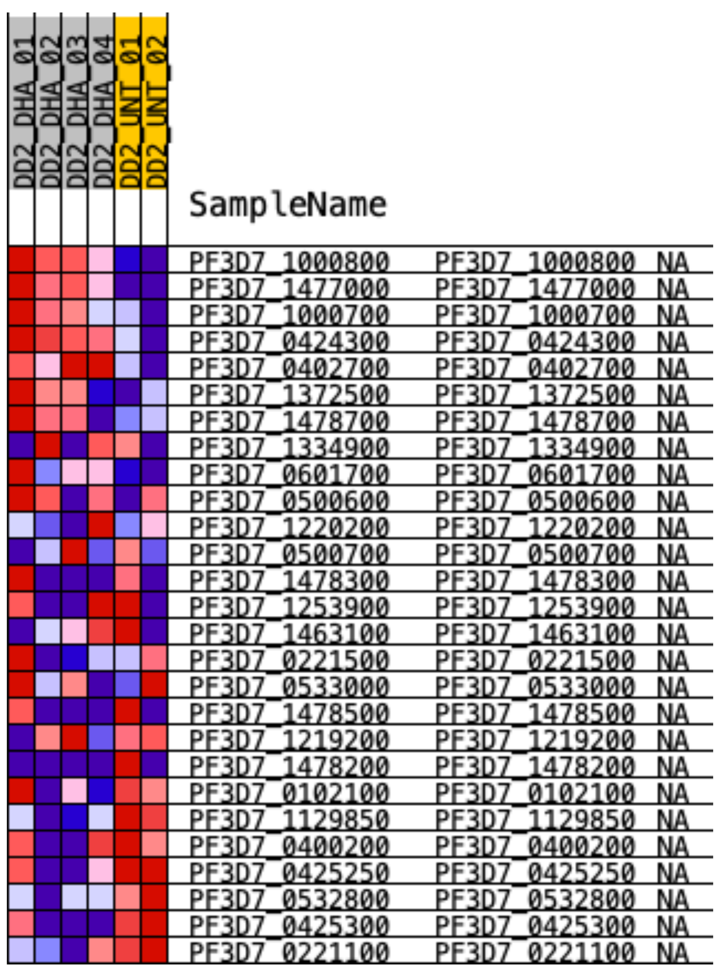

Fig 2: ME0
Blue-Pink O' Gram in the Space of the Analyzed GeneSet

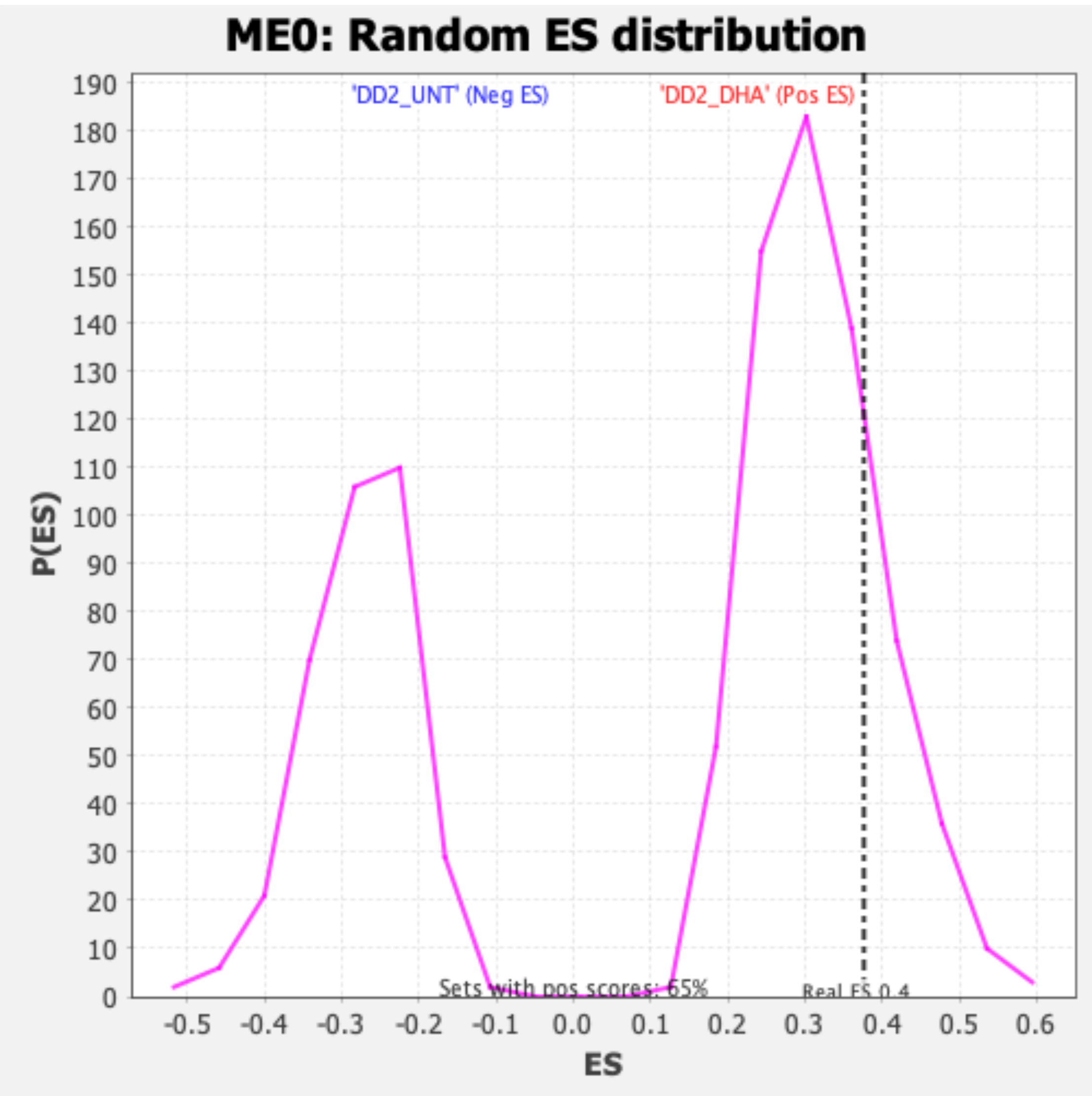

Fig 3: ME0: Random ES distribution
Gene set null distribution of ES for ME0
