# Supplementary material for: Transcriptome-module phenotype association study implicates extracellular vesicles biogenesis in Plasmodium falciparum artemisinin resistance
Source: Front Cell Infect Microbiol. 2022 Aug 19;12:886728. doi: 10.3389/fcimb.2022.886728 (PMC9437462; doi:10.3389/fcimb.2022.886728)
Supplement: Supplementary file 1 [file DataSheet_1.zip › Supplementary_files/Supplementary_Data_5.pdf]

Table: GSEA Results Summary

Dataset	Expression_dataset_dataset_collapsed_to_symbols.PhenotypeData.cls #C580R_DHA_versus_C580R_DMSO.PhenotypeData.cls #C580R_DHA_versus_C580R_DMSO_repos
Phenotype	PhenotypeData.cls#C580R_DHA_versus_C580R_DMSO_repos
Upregulated in class	C580R_DHA
GeneSet	ME0
Enrichment Score (ES)	0.312271
Normalized Enrichment Score (NES)	0.7299921
Nominal p-value	0.8987854
FDR q-value	0.891
FWER p-Value	0.984

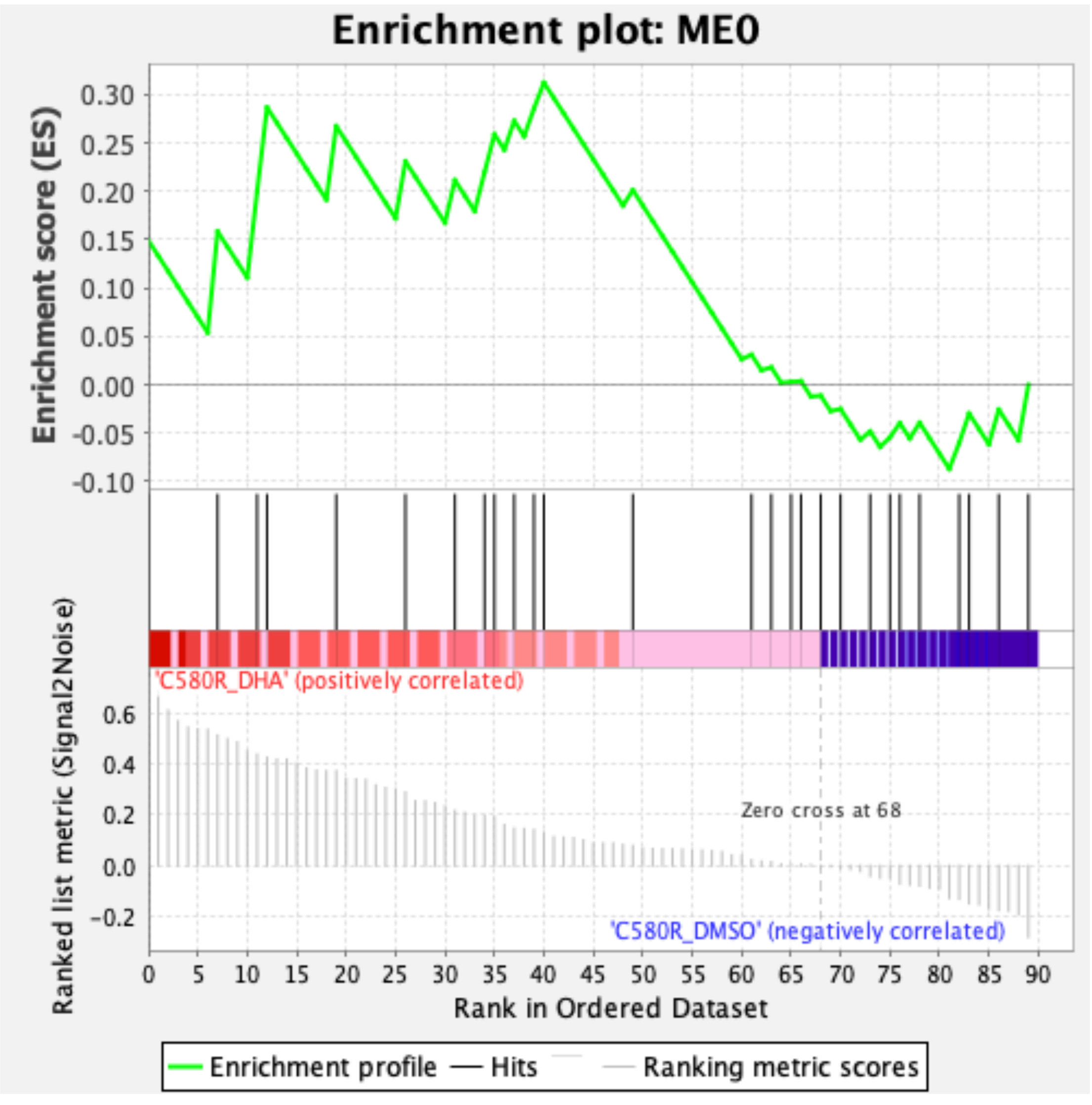

Fig 1: Enrichment plot: ME0
Profile of the Running ES Score & Positions of GeneSet Members on the Rank Ordered List

Table: GSEA details [plain text format]

	SYMBOL	TITLE	RANK IN GENE LIST	RANK METRIC SCORE	RUNNING ES	CORE ENRICHMENT
1	PF3D7_0500600	NA	0	0.730	0.1491	Yes
2	PF3D7_0221500	NA	7	0.512	0.1584	Yes
3	PF3D7_1000700	NA	11	0.437	0.1999	Yes
4	PF3D7_0533000	NA	12	0.424	0.2866	Yes
5	PF3D7_1000800	NA	19	0.372	0.2673	Yes
6	PF3D7_0500700	NA	26	0.288	0.2309	Yes
7	PF3D7_1477000	NA	31	0.215	0.2113	Yes
8	PF3D7_0425300	NA	34	0.196	0.2196	Yes
9	PF3D7_0425250	NA	35	0.192	0.2588	Yes
10	PF3D7_1478700	NA	37	0.146	0.2727	Yes
11	PF3D7_1478500	NA	39	0.141	0.2856	Yes
12	PF3D7_1463100	NA	40	0.131	0.3123	Yes
13	PF3D7_1372500	NA	49	0.078	0.2011	No
14	PF3D7_1220200	NA	61	0.022	0.0310	No
15	PF3D7_0601700	NA	63	0.014	0.0179	No
16	PF3D7_1219200	NA	65	0.004	0.0029	No
17	PF3D7_0221100	NA	66	0.004	0.0037	No
18	PF3D7_1478200	NA	68	-0.004	-0.0113	No
19	PF3D7_0402700	NA	70	-0.010	-0.0251	No
20	PF3D7_0400200	NA	73	-0.041	-0.0484	No
21	PF3D7_0102100	NA	75	-0.048	-0.0543	No
22	PF3D7_0532800	NA	76	-0.072	-0.0397	No
23	PF3D7_1478300	NA	78	-0.079	-0.0394	No
24	PF3D7_0424300	NA	82	-0.131	-0.0602	No
25	PF3D7_1253900	NA	83	-0.149	-0.0298	No
26	PF3D7_1334900	NA	86	-0.175	-0.0257	No
27	PF3D7_1129850	NA	89	-0.281	-0.0000	No

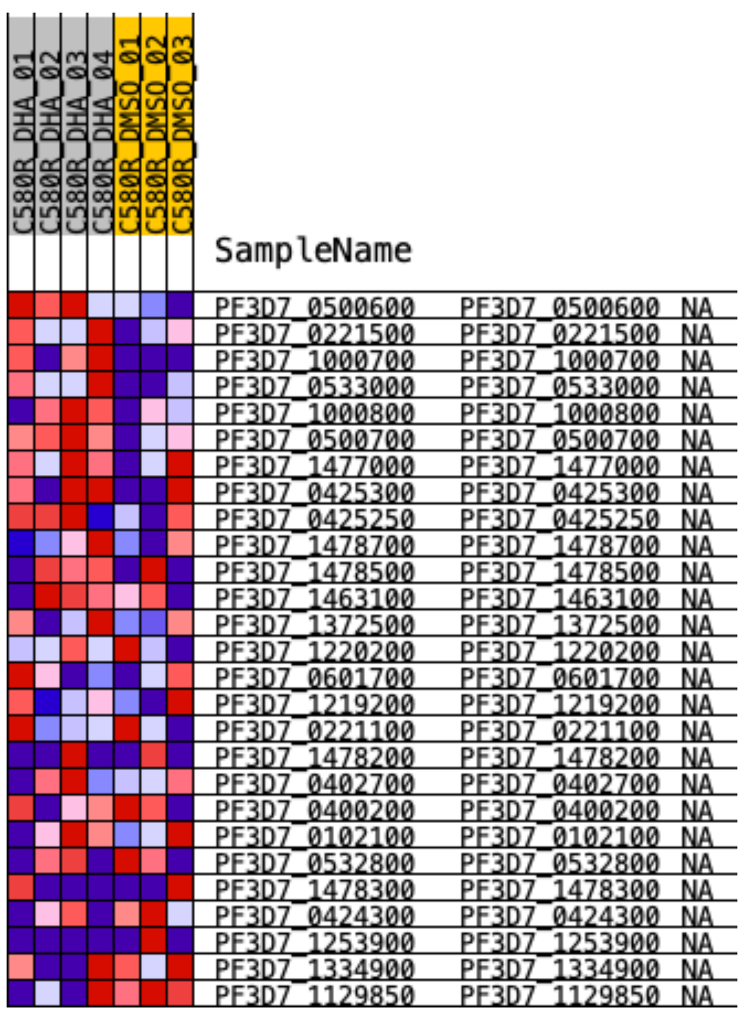

Fig 2: ME0
Blue-Pink O' Gram in the Space of the Analyzed GeneSet

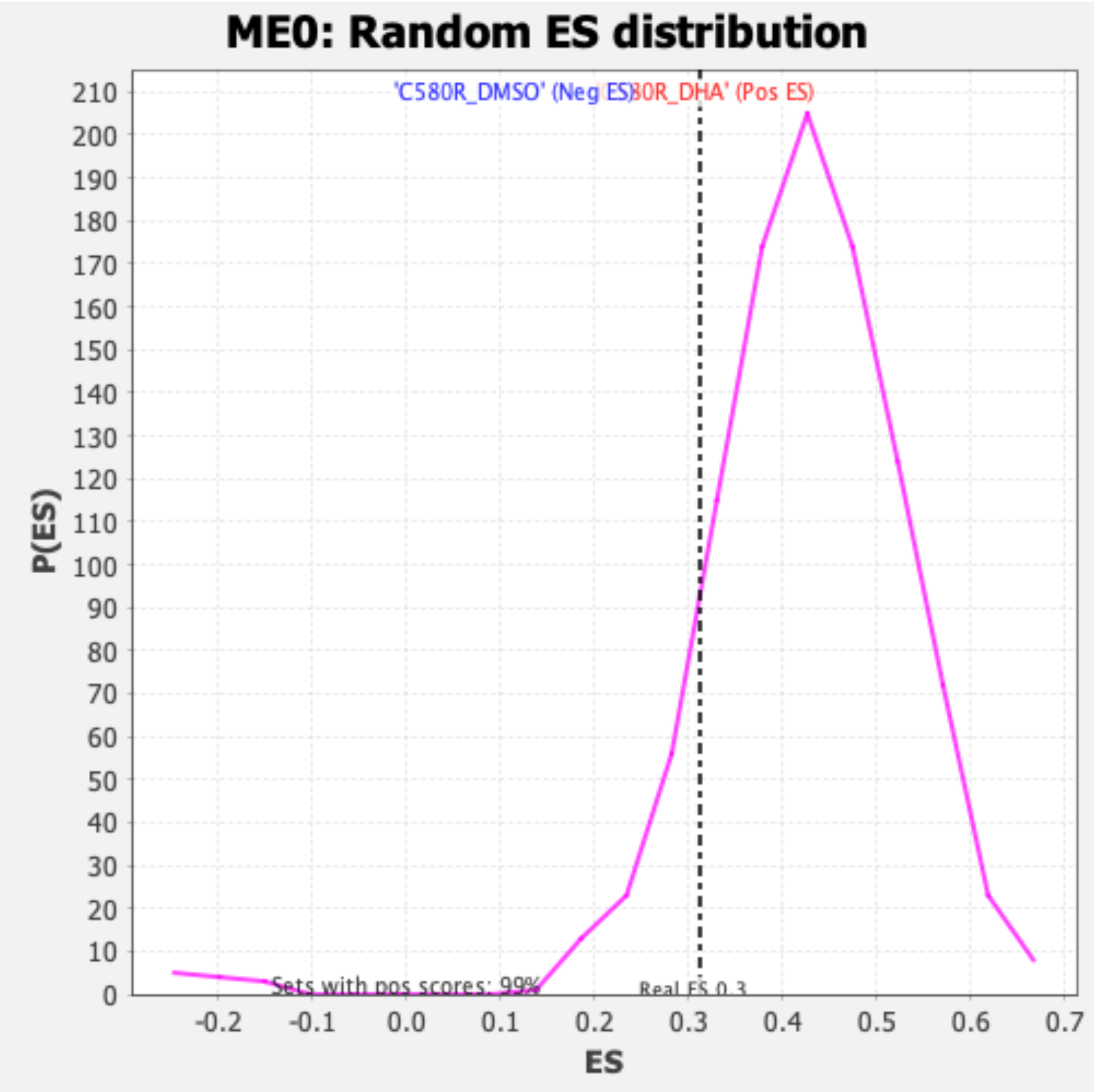

Fig 3: ME0: Random ES distribution
Gene set null distribution of ES for ME0
