# Supplementary material for: Transcriptome-module phenotype association study implicates extracellular vesicles biogenesis in Plasmodium falciparum artemisinin resistance
Source: Front Cell Infect Microbiol. 2022 Aug 19;12:886728. doi: 10.3389/fcimb.2022.886728 (PMC9437462; doi:10.3389/fcimb.2022.886728)
Supplement: Supplementary file 1 [file DataSheet_1.zip › Supplementary_files/Supplementary_Data_4.pdf]

Table: GSEA Results Summary

Dataset	Expression_dataset_dataset_collapsed_to_symbols.PhenotypeData.cls<br>#C580R_DHA_versus_C580R_UNT.PhenotypeData.cls<br>#C580R_DHA_versus_C580R_UNT_repos
Phenotype	PhenotypeData.cls#C580R_DHA_versus_C580R_UNT_repos
Upregulated in class	C580R_DHA
GeneSet	ME0
Enrichment Score (ES)	0.309629
Normalized Enrichment Score (NES)	1.2457103
Nominal p-value	0.11764706
FDR q-value	0.14403293
FWER p-Value	0.037

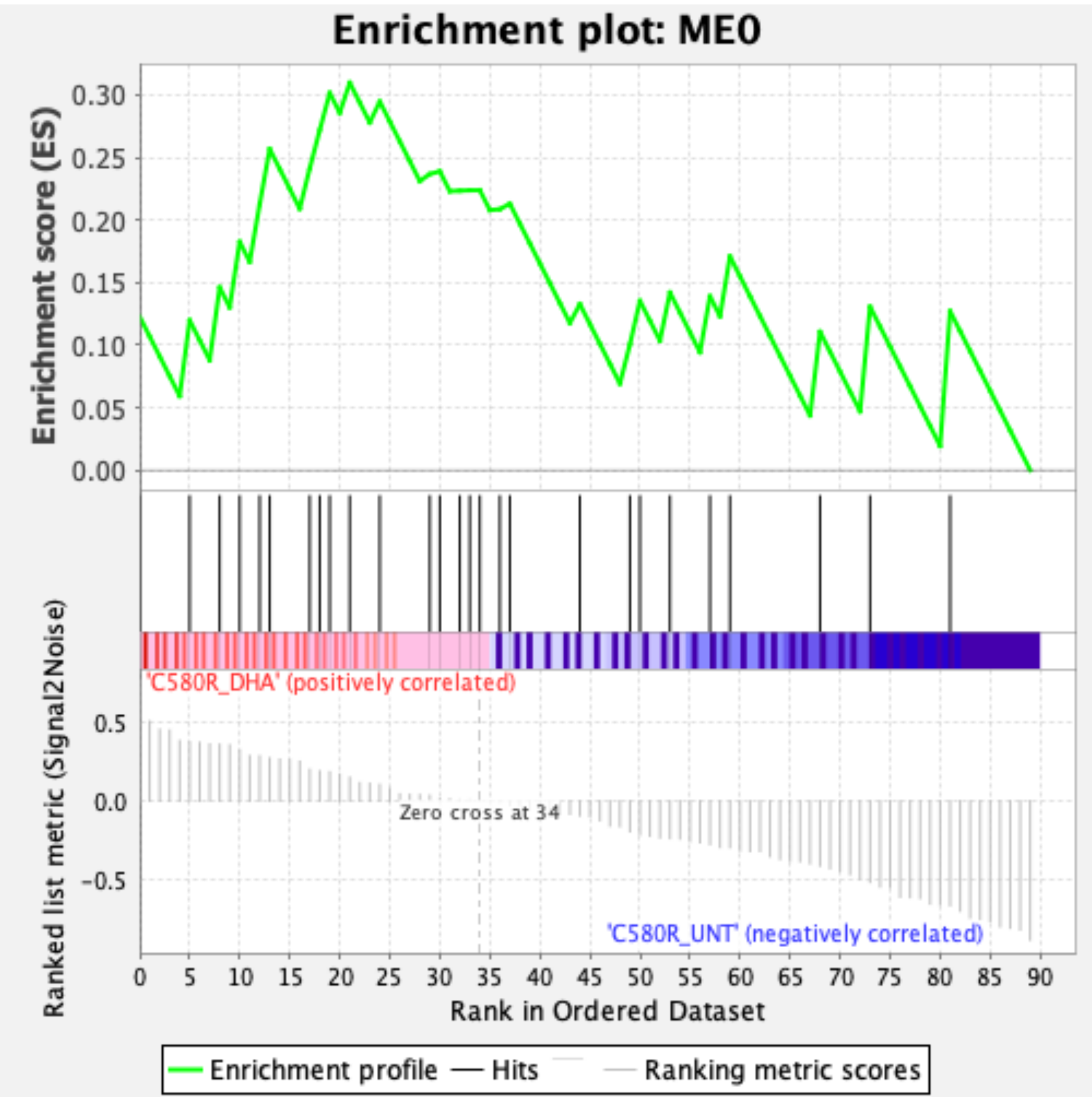

Fig 1: Enrichment plot: ME0
Profile of the Running ES Score & Positions of GeneSet Members on the Rank Ordered List

Table: GSEA details [plain text format]

	SYMBOL	TITLE	RANK IN GENE LIST	RANK METRIC SCORE	RUNNING ES	CORE ENRICHMENT
1	PF3D7_1372500	NA	0	0.754	0.1228	Yes
2	PF3D7_1478700	NA	5	0.371	0.1197	Yes
3	PF3D7_0533000	NA	8	0.357	0.1461	Yes
4	PF3D7_1478500	NA	10	0.321	0.1825	Yes
5	PF3D7_0425250	NA	12	0.283	0.2126	Yes
6	PF3D7_0500700	NA	13	0.270	0.2566	Yes
7	PF3D7_0601700	NA	17	0.198	0.2412	Yes
8	PF3D7_0532800	NA	18	0.188	0.2718	Yes
9	PF3D7_1220200	NA	19	0.182	0.3015	Yes
10	PF3D7_0500600	NA	21	0.148	0.3096	Yes
11	PF3D7_1478200	NA	24	0.103	0.2946	No
12	PF3D7_0425300	NA	29	0.034	0.2367	No
13	PF3D7_0221500	NA	30	0.013	0.2389	No
14	PF3D7_1000800	NA	32	0.002	0.2234	No
15	PF3D7_0400200	NA	33	0.002	0.2237	No
16	PF3D7_1253900	NA	34	0.000	0.2237	No
17	PF3D7_0402700	NA	36	-0.005	0.2086	No
18	PF3D7_1000700	NA	37	-0.026	0.2129	No
19	PF3D7_1477000	NA	44	-0.092	0.1327	No
20	PF3D7_1334900	NA	49	-0.193	0.1006	No
21	PF3D7_1129850	NA	50	-0.213	0.1353	No
22	PF3D7_1478300	NA	53	-0.236	0.1419	No
23	PF3D7_1463100	NA	57	-0.274	0.1389	No
24	PF3D7_1219200	NA	59	-0.293	0.1708	No
25	PF3D7_0102100	NA	68	-0.410	0.1105	No
26	PF3D7_0221100	NA	73	-0.513	0.1305	No
27	PF3D7_0424300	NA	81	-0.661	0.1270	No

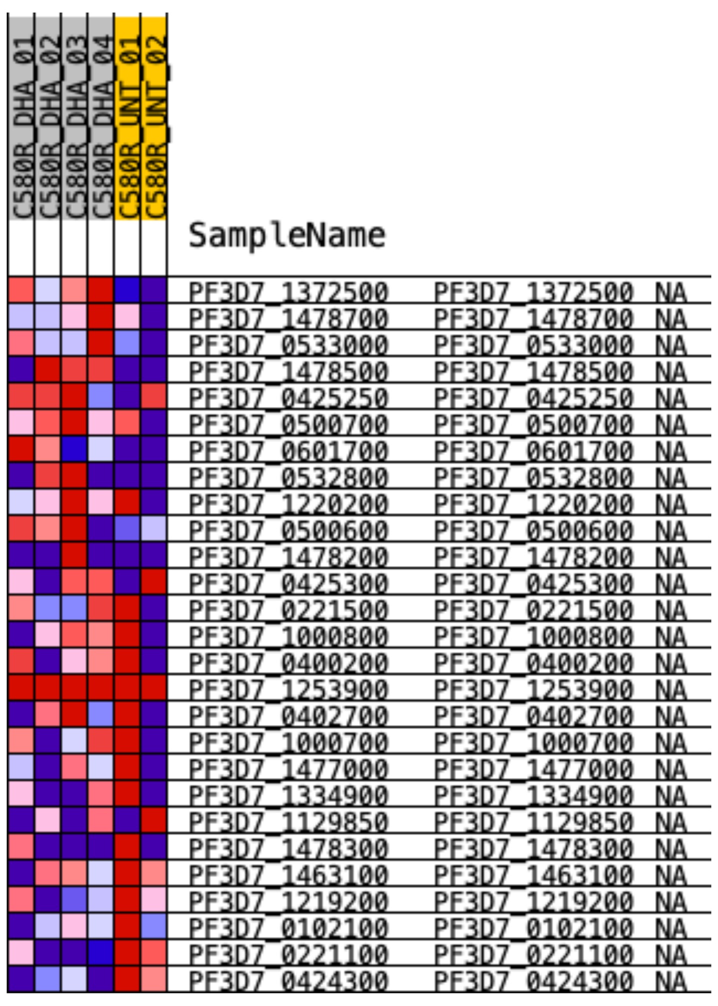

Fig 2: ME0
Blue-Pink O' Gram in the Space of the Analyzed GeneSet

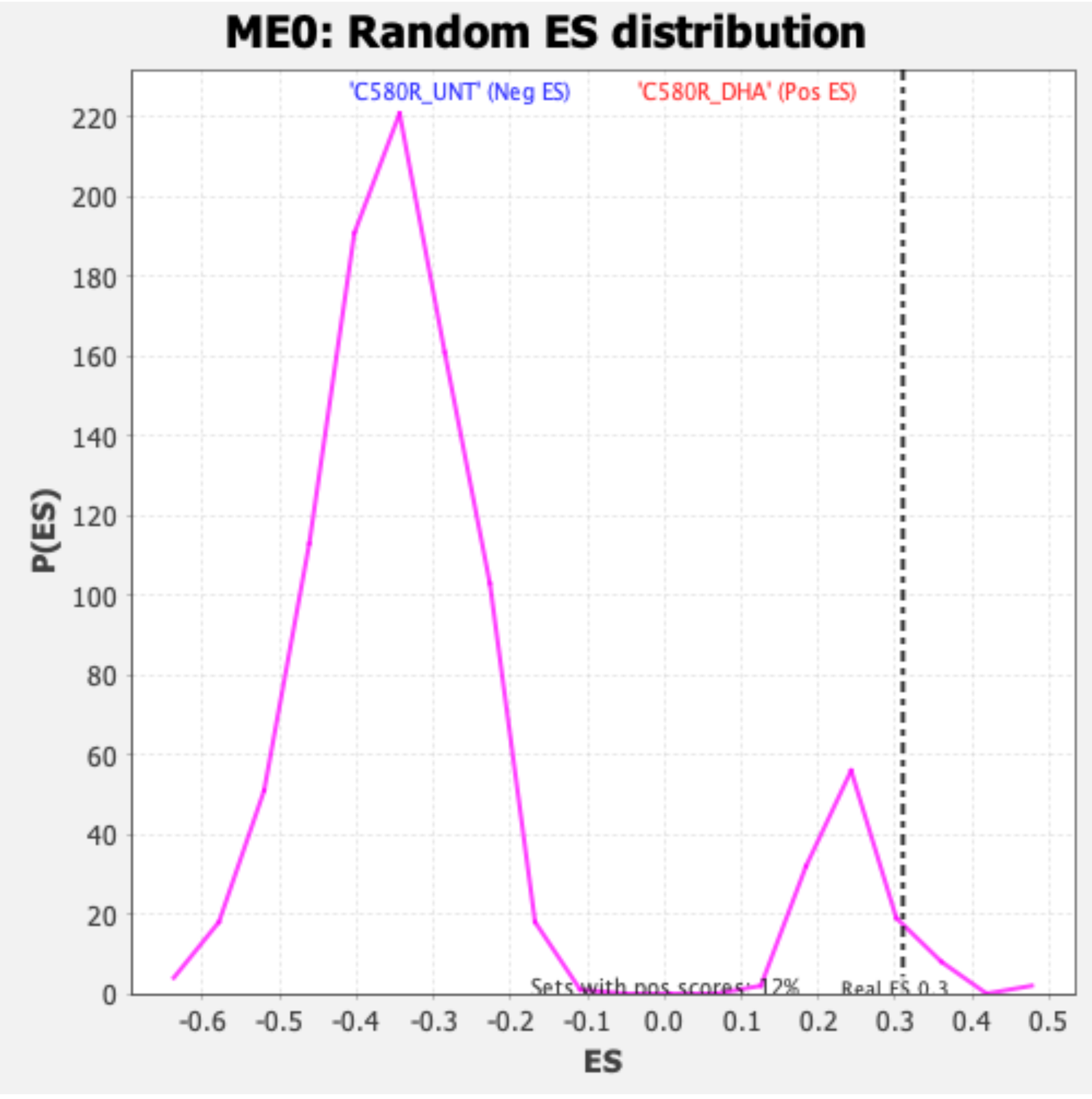

Fig 3: ME0: Random ES distribution
Gene set null distribution of ES for ME0
