# Supplementary material for: Transcriptome-module phenotype association study implicates extracellular vesicles biogenesis in Plasmodium falciparum artemisinin resistance
Source: Front Cell Infect Microbiol. 2022 Aug 19;12:886728. doi: 10.3389/fcimb.2022.886728 (PMC9437462; doi:10.3389/fcimb.2022.886728)
Supplement: Supplementary file 1 [file DataSheet_1.zip › Supplementary_files/Supplementary_Data_9.pdf]

*Table: GSEA Results Summary*

| | |
|---|---|
| Dataset | Expression_dataset_dataset_collapsed_to_symbols.PhenotypeData.cls #R539T_DHA_versus_R539T_UNT.PhenotypeData.cls #R539T_DHA_versus_R539T_UNT_repos |
| Phenotype | PhenotypeData.cls#R539T_DHA_versus_R539T_UNT_repos |
| Upregulated in class | R539T_UNT |
| GeneSet | ME7 |
| Enrichment Score (ES) | -0.34375 |
| Normalized Enrichment Score (NES) | -1.2690192 |
| Nominal p-value | 0.14084508 |
| FDR q-value | 0.14516129 |
| FWER p-Value | 0.044 |

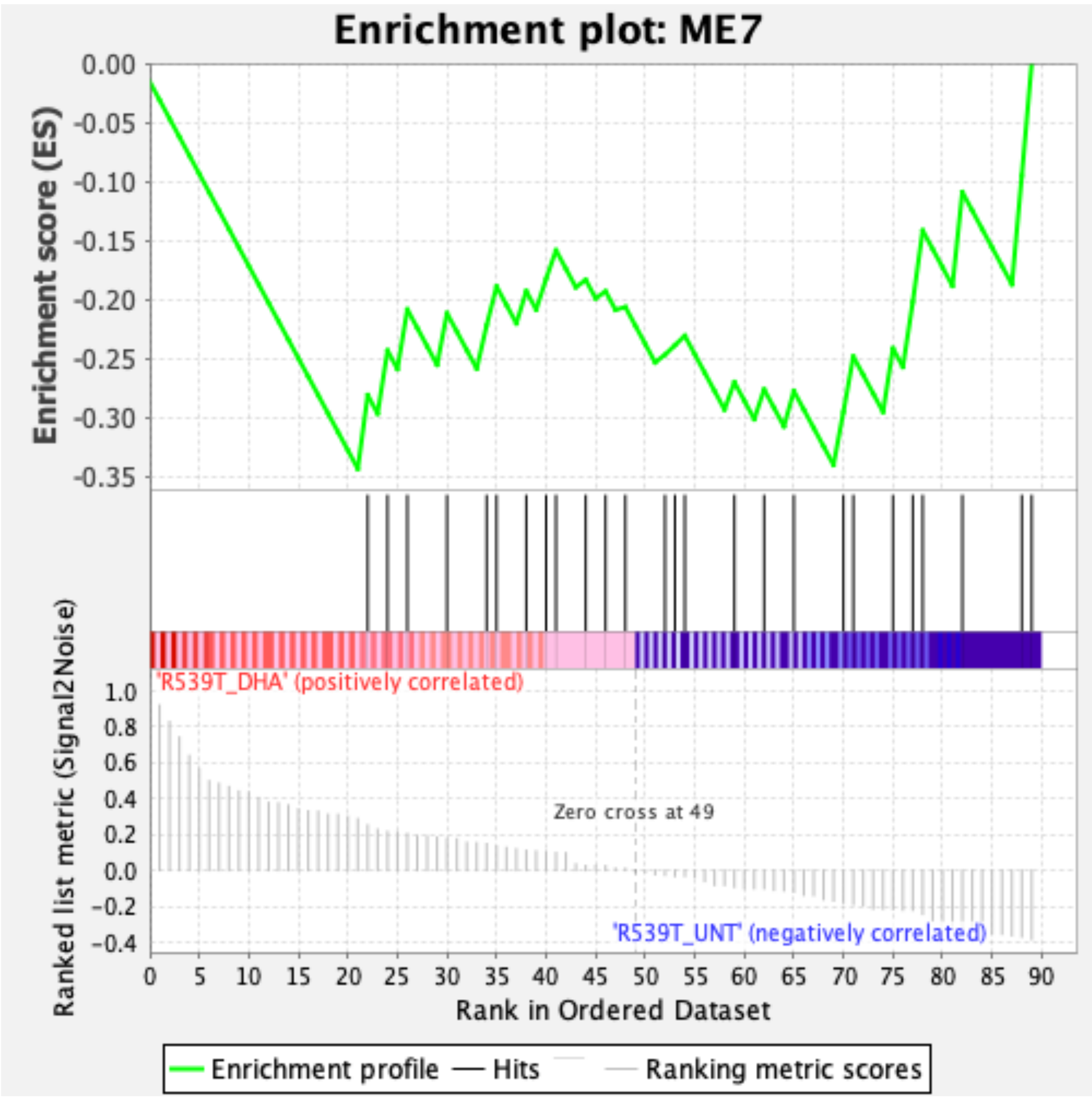


**Fig 1: Enrichment plot: ME7**
**Profile of the Running ES Score & Positions of GeneSet Members on the Rank Ordered List**

*Table: GSEA details* [plain text format]

| | SYMBOL | TITLE | RANK IN GENE LIST | RANK METRIC SCORE | RUNNING ES | CORE ENRICHMENT |
|---|---|---|---|---|---|---|
| 1 | PF3D7_0115150 | NA | 22 | 0.250 | -0.2813 | No |
| 2 | PF3D7_0401500 | NA | 24 | 0.214 | -0.2436 | No |
| 3 | PF3D7_1240700 | NA | 26 | 0.202 | -0.2087 | No |
| 4 | PF3D7_1480100 | NA | 30 | 0.175 | -0.2120 | No |
| 5 | PF3D7_1400100 | NA | 34 | 0.146 | -0.2223 | No |
| 6 | PF3D7_0221650 | NA | 35 | 0.132 | -0.1892 | No |
| 7 | PF3D7_0114400 | NA | 38 | 0.109 | -0.1932 | No |
| 8 | PF3D7_0712500 | NA | 40 | 0.104 | -0.1828 | No |
| 9 | PF3D7_0413400 | NA | 41 | 0.097 | -0.1586 | No |
| 10 | PF3D7_0425000 | NA | 44 | 0.025 | -0.1837 | No |
| 11 | PF3D7_1240200 | NA | 46 | 0.024 | -0.1934 | No |
| 12 | PF3D7_0302300 | NA | 48 | 0.009 | -0.2067 | No |
| 13 | PF3D7_0402800 | NA | 52 | -0.026 | -0.2471 | No |
| 14 | PF3D7_0632600 | NA | 53 | -0.032 | -0.2391 | No |
| 15 | PF3D7_0713300 | NA | 54 | -0.032 | -0.2310 | No |
| 16 | PF3D7_1219400 | NA | 59 | -0.093 | -0.2704 | No |
| 17 | PF3D7_1000900 | NA | 62 | -0.101 | -0.2766 | No |
| 18 | PF3D7_1219500 | NA | 65 | -0.120 | -0.2779 | No |
| 19 | PF3D7_0114300 | NA | 70 | -0.180 | -0.2956 | Yes |
| 20 | PF3D7_1401050 | NA | 71 | -0.188 | -0.2487 | Yes |
| 21 | PF3D7_0421600 | NA | 75 | -0.215 | -0.2418 | Yes |
| 22 | PF3D7_0221300 | NA | 77 | -0.220 | -0.2024 | Yes |
| 23 | PF3D7_0421500 | NA | 78 | -0.243 | -0.1418 | Yes |
| 24 | PF3D7_0221900 | NA | 82 | -0.318 | -0.1093 | Yes |
| 25 | PF3D7_1478400 | NA | 88 | -0.369 | -0.0954 | Yes |
| 26 | PF3D7_0114600 | NA | 89 | -0.382 | -0.0000 | Yes |

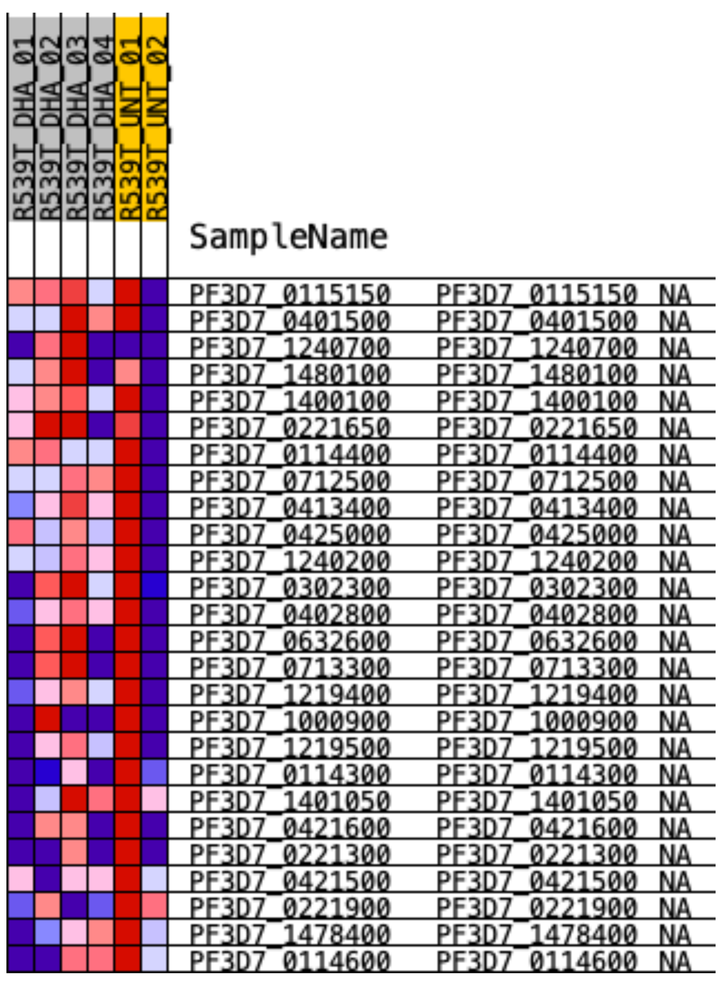


**Fig 2: ME7**
**Blue-Pink O' Gram in the Space of the Analyzed GeneSet**

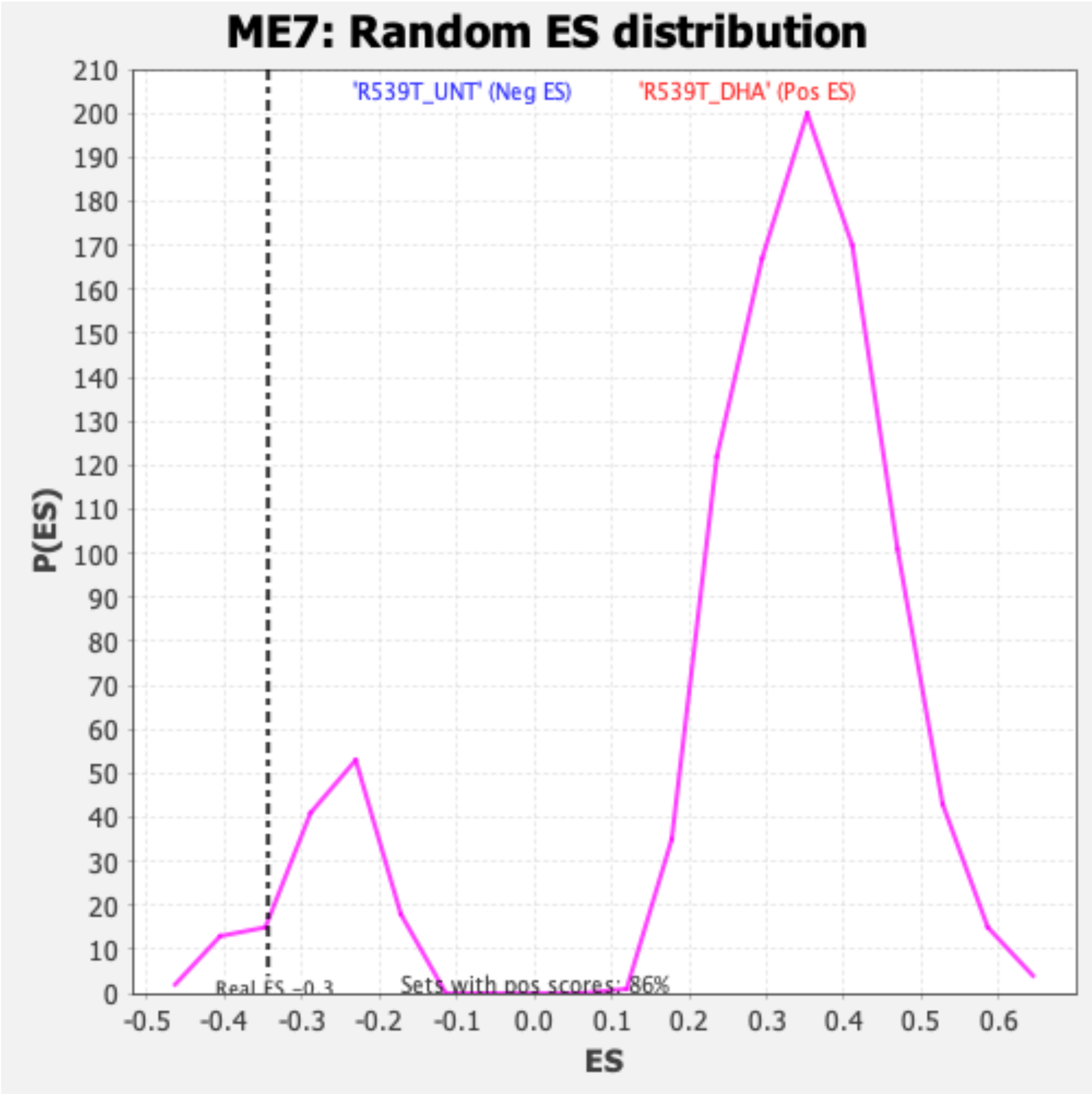


**Fig 3: ME7: Random ES distribution**
**Gene set null distribution of ES for ME7**
