# Supplementary material for: Transcriptome-module phenotype association study implicates extracellular vesicles biogenesis in Plasmodium falciparum artemisinin resistance
Source: Front Cell Infect Microbiol. 2022 Aug 19;12:886728. doi: 10.3389/fcimb.2022.886728 (PMC9437462; doi:10.3389/fcimb.2022.886728)
Supplement: Supplementary file 1 [file DataSheet_1.zip › Supplementary_files/Supplementary_Data_17.pdf]

	UP regulated	DOWN regulated
DD2	Nil significantly enriched biological processes	biological process involved in symbiotic interaction<br>biological process involved in interspecies interaction between organisms<br>movement in host environment<br>entry into host<br>biological process<br>biological process involved in interaction with host<br>immunoglobulin production<br>production of molecular mediator of immune response<br>regulation of immune response<br>regulation of immune system process<br>immune system process<br>obsolete pathogenesis<br>cell adhesion<br>biological adhesion<br>cell-cell adhesion
C580R	obsolete pathogenesis<br>cell-cell adhesion<br>cell adhesion<br>cytoadherence to microvasculature, mediated by symbiont protein<br>adhesion of symbiont to host<br>antigenic variation<br>modulation by symbiont of host erythrocyte aggregation<br>modulation by symbiont of host cellular process<br>evasion of host immune response<br>modulation by symbiont of host process<br>biological adhesion<br>response to host immune response<br>response to host defenses<br>response to defenses of other organism<br>response to other organism<br>response to external biotic stimulus<br>response to host<br>biological process involved in interaction with host<br>biological process involved in symbiotic interaction<br>biological process involved in interspecies interaction between organisms<br>biological process	movement in host environment<br>entry into host<br>biological process involved in symbiotic interaction<br>biological process involved in interspecies interaction between organisms<br>biological process<br>biological process involved in interaction with host<br>actin filament-based process<br>actin cytoskeleton organization<br>actin filament organization<br>actin filament-based movement<br>exit from host cell<br>exit from host<br>supramolecular fiber organization<br>cell motility<br>regulation of immune response<br>production of molecular mediator of immune response<br>immunoglobulin production<br>regulation of immune system process<br>immune system process<br>actin polymerization or depolymerization<br>actin filament-based transport<br>vesicle transport along actin filament
R539T	Nil significantly enriched biological processes	movement in host environment<br>entry into host<br>biological process involved in symbiotic interaction<br>biological process involved in interspecies interaction between organisms<br>biological process involved in interaction with host<br>biological process<br>exit from host cell<br>exit from host<br>regulation of immune response<br>immunoglobulin production<br>production of molecular mediator of immune response<br>regulation of immune system process<br>immune system process<br>actin filament organization<br>actin filament-based process<br>actin cytoskeleton organization<br>supramolecular fiber organization<br>protein phosphorylation

Supplementary Data 17A | Summary of gene ontology enrichment findings in the biological processes term for DHA_treated condition compared to the untreated condition baseline

	UP regulated	DOWN regulated
DD2	Nil significantly enriched biological processes	protein binding<br>host cell surface binding<br>host cell surface receptor binding<br>long-chain fatty acid-CoA ligase activity<br>decanoate-CoA ligase activity<br>fatty acid ligase activity<br>medium-chain fatty acid-CoA ligase activity<br>CoA-ligase activity<br>acid-thiol ligase activity<br>cysteine-type endopeptidase activity<br>cysteine-type peptidase activity
C580R	cell adhesion molecule binding<br>host cell surface receptor binding<br>host cell surface binding	host cell surface binding<br>actin binding<br>protein binding<br>actin filament binding<br>heparin binding<br>glycosaminoglycan binding<br>actin monomer binding<br>protein-containing complex binding<br>protein heterodimerization activity<br>cytoskeletal protein binding<br>actin-dependent ATPase activity
R539T	Nil significantly enriched biological processes	host cell surface binding<br>heparin binding<br>glycosaminoglycan binding<br>protein binding<br>host cell surface receptor binding<br>protein kinase activity<br>actin filament binding

Supplementary Data 17B | Summary of gene ontology enrichment findings in the molecular function term for DHA_treated condition compared to the untreated condition baseline

	UP regulated	DOWN regulated
DD2	crystalloid<br>osmiophilic body	host cellular component<br>host cell<br>host cell part<br>host cell cytoplasm<br>host intracellular part<br>host intracellular region<br>host cell cytoplasm part<br>extracellular vesicle<br>extracellular region<br>extracellular membrane-bounded organelle<br>extracellular organelle<br>Maurer's cleft<br>other organism part<br>apical complex<br>vesicle<br>apical part of cell<br>rhoptry<br>host cell periphery<br>symbiont-containing vacuole membrane<br>lytic vacuole<br>lysosome<br>symbiont-containing vacuole<br>vacuole<br>host cell cytosol<br>host cell surface<br>merozoite dense granule<br>extracellular space<br>infected host cell surface knob<br>rhoptry neck<br>cell surface
C580R	infected host cell surface knob<br>host cell plasma membrane<br>host cell membrane<br>host cell part<br>host cell cytoplasm part<br>host cell cytoplasm<br>host cell<br>host cellular component<br>host intracellular part<br>host intracellular region<br>integral component of membrane<br>intrinsic component of membrane	apical part of cell<br>apical complex<br>rhoptry<br>cell surface<br>extracellular vesicle<br>rhoptry neck<br>extracellular membrane-bounded organelle<br>extracellular region<br>extracellular organelle<br>vesicle<br>pellicle<br>inner membrane pellicle complex<br>lytic vacuole<br>lysosome<br>anchored component of membrane<br>vacuole<br>symbiont-containing vacuolar space<br>symbiont-containing vacuole<br>microneme<br>anchored component of plasma membrane<br>actin cytoskeleton<br>nucleosome<br>secondary lysosome<br>food vacuole<br>phagocytic vesicle<br>phagolysosome<br>extracellular space<br>symbiont-containing vacuole membrane<br>endocytic vesicle<br>host intracellular part<br>host intracellular region<br>intrinsic component of plasma membrane<br>host cell cytoplasm part<br>host cell part<br>host cell cytoplasm<br>cytoplasmic vesicle<br>myosin complex<br>intracellular vesicle

		protein-containing complex<br>plasma membrane<br>rhoptry membrane<br>DNA packaging complex
R539T	crystalloid	apical complex<br>apical part of cell<br>rhoptry<br>rhoptry neck<br>extracellular vesicle<br>cell surface<br>extracellular region<br>host cell<br>host cellular component<br>extracellular membrane-bounded organelle<br>extracellular organelle<br>vesicle<br>host cell part<br>symbiont-containing vacuolar space<br>anchored component of membrane<br>microneme<br>pellicle<br>host intracellular part<br>host intracellular region<br>host cell cytoplasm<br>host cell cytoplasm part<br>anchored component of plasma membrane<br>inner membrane pellicle complex<br>extracellular space<br>symbiont-containing vacuole<br>lytic vacuole<br>lysosome<br>vacuole<br>intrinsic component of plasma membrane

Supplementary Data 17C | Summary of gene ontology enrichment findings in the cellular component term for DHA_treated condition compared to the untreated condition baseline
