# Supplementary material for: Transcriptome-module phenotype association study implicates extracellular vesicles biogenesis in Plasmodium falciparum artemisinin resistance
Source: Front Cell Infect Microbiol. 2022 Aug 19;12:886728. doi: 10.3389/fcimb.2022.886728 (PMC9437462; doi:10.3389/fcimb.2022.886728)
Supplement: Supplementary file 1 [file DataSheet_1.zip › Supplementary_files/Supplementary_Data_16.pdf]

Table: GSEA Results Summary

Dataset	Expression_dataset_dataset_collapsed_to_symbols.PhenotypeData.cls<br>#DD2_DHA_versus_DD2_DMSO.PhenotypeData.cls<br>#DD2_DHA_versus_DD2_DMSO_repos
Phenotype	PhenotypeData.cls#DD2_DHA_versus_DD2_DMSO_repos
Upregulated in class	DD2_DMSO
GeneSet	ME7
Enrichment Score (ES)	-0.21875
Normalized Enrichment Score (NES)	-0.9836254
Nominal p-value	0.42857143
FDR q-value	0.51960784
FWER p-Value	0.05

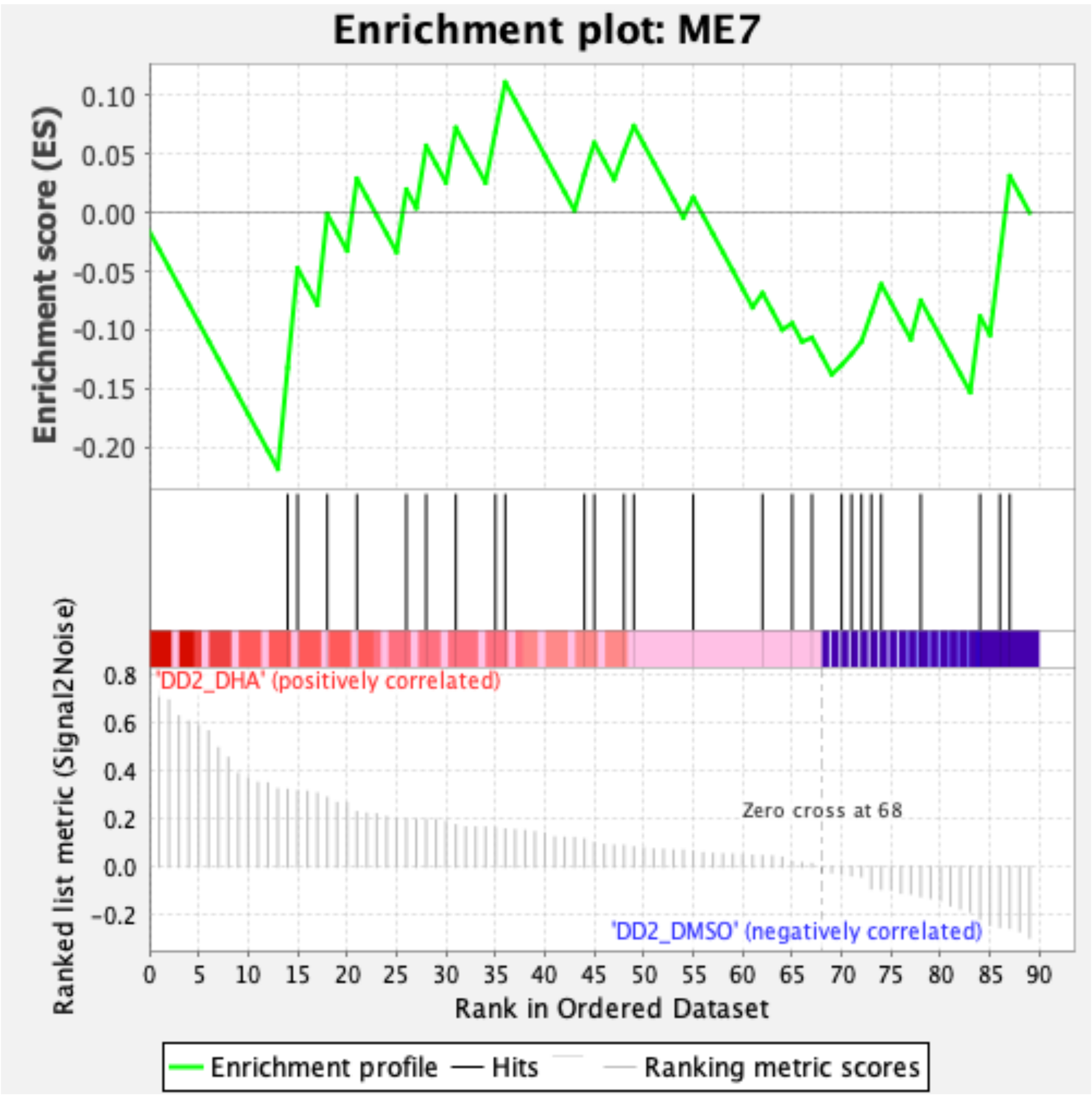

Fig 1: Enrichment plot: ME7
Profile of the Running ES Score & Positions of GeneSet Members on the Rank Ordered List

Table: GSEA details [plain text format]

	SYMBOL	TITLE	RANK IN GENE LIST	RANK METRIC SCORE	RUNNING ES	CORE ENRICHMENT
1	PF3D7_1480100	NA	14	0.320	-0.1323	Yes
2	PF3D7_1000900	NA	15	0.314	-0.0474	Yes
3	PF3D7_0114300	NA	18	0.287	-0.0012	Yes
4	PF3D7_1219400	NA	21	0.227	0.0290	Yes
5	PF3D7_0114400	NA	26	0.197	0.0197	Yes
6	PF3D7_0421500	NA	28	0.196	0.0571	Yes
7	PF3D7_1240200	NA	31	0.173	0.0725	Yes
8	PF3D7_1478400	NA	35	0.161	0.0692	Yes
9	PF3D7_1401050	NA	36	0.156	0.1113	Yes
10	PF3D7_0632600	NA	44	0.114	0.0328	Yes
11	PF3D7_0401500	NA	45	0.100	0.0597	Yes
12	PF3D7_0712500	NA	48	0.088	0.0521	Yes
13	PF3D7_0221650	NA	49	0.081	0.0740	Yes
14	PF3D7_0302300	NA	55	0.064	0.0131	Yes
15	PF3D7_0713300	NA	62	0.045	-0.0686	Yes
16	PF3D7_0413400	NA	65	0.020	-0.0945	Yes
17	PF3D7_1219500	NA	67	0.013	-0.1068	Yes
18	PF3D7_0425000	NA	70	-0.029	-0.1301	Yes
19	PF3D7_0421600	NA	71	-0.035	-0.1206	Yes
20	PF3D7_1240700	NA	72	-0.039	-0.1101	Yes
21	PF3D7_0114600	NA	73	-0.090	-0.0858	Yes
22	PF3D7_0221300	NA	74	-0.091	-0.0612	Yes
23	PF3D7_1400100	NA	78	-0.122	-0.0751	Yes
24	PF3D7_0115150	NA	84	-0.239	-0.0887	Yes
25	PF3D7_0402800	NA	86	-0.249	-0.0370	Yes
26	PF3D7_0221900	NA	87	-0.253	0.0312	Yes

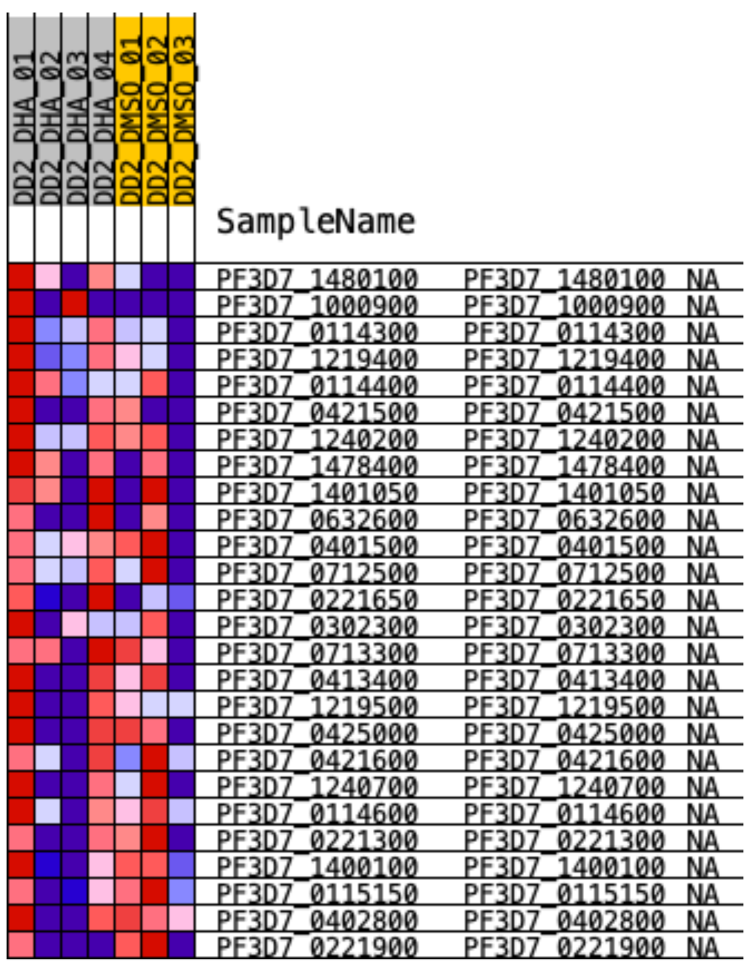

Fig 2: ME7
Blue-Pink O' Gram in the Space of the Analyzed GeneSet

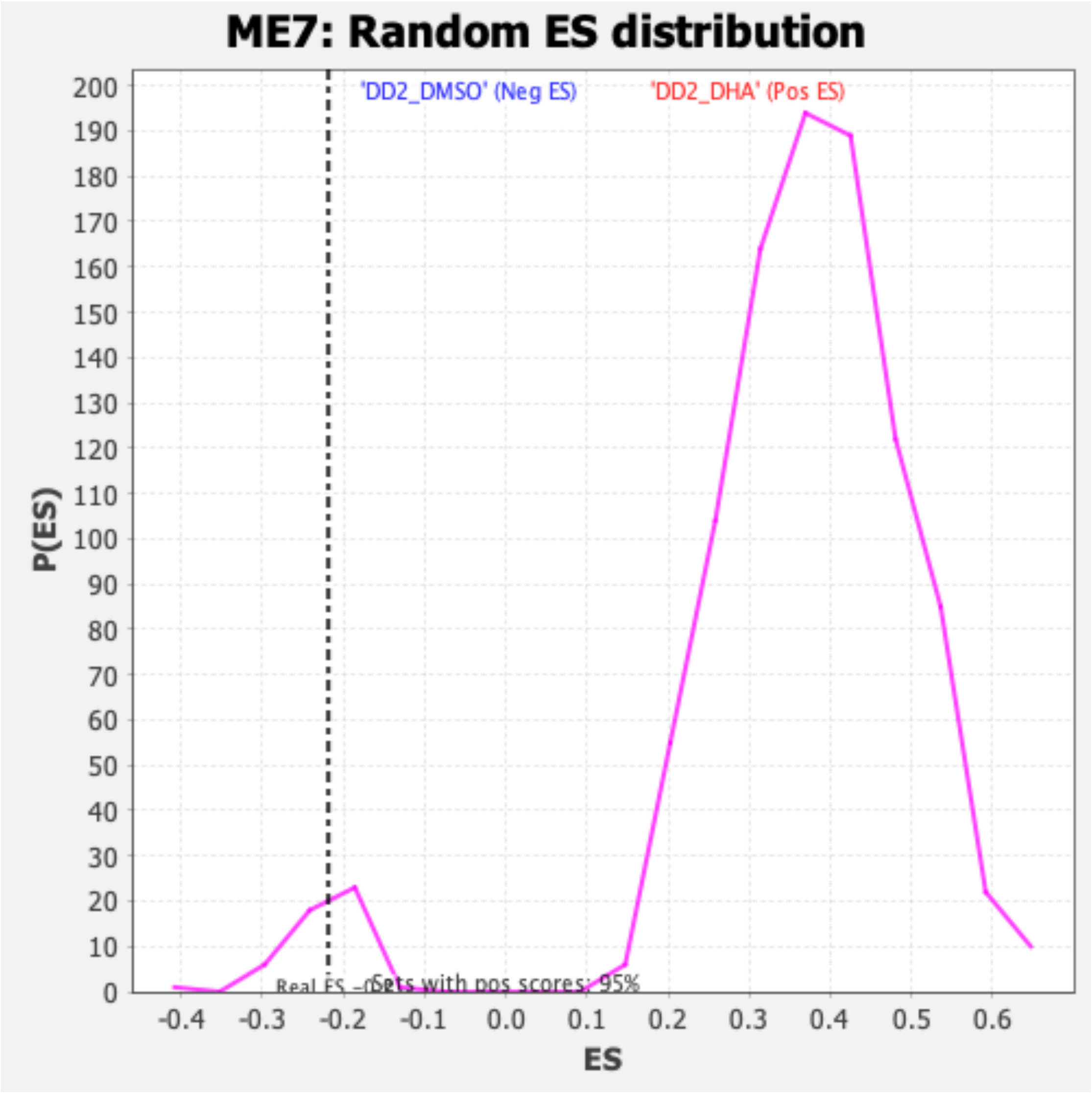

Fig 3: ME7: Random ES distribution
Gene set null distribution of ES for ME7
