# Supplementary material for: Transcriptome-module phenotype association study implicates extracellular vesicles biogenesis in Plasmodium falciparum artemisinin resistance
Source: Front Cell Infect Microbiol. 2022 Aug 19;12:886728. doi: 10.3389/fcimb.2022.886728 (PMC9437462; doi:10.3389/fcimb.2022.886728)
Supplement: Supplementary file 1 [file DataSheet_1.zip › Supplementary_files/Supplementary_Data_8.pdf]

Table: GSEA Results Summary

Dataset	Expression_dataset_dataset_collapsed_to_symbols.PhenotypeData.cls<br>#DD2_DHA_versus_DD2_DMSO.PhenotypeData.cls<br>#DD2_DHA_versus_DD2_DMSO_repos
Phenotype	PhenotypeData.cls#DD2_DHA_versus_DD2_DMSO_repos
Upregulated in class	DD2_DMSO
GeneSet	ME0
Enrichment Score (ES)	-0.26537684
Normalized Enrichment Score (NES)	-1.1928045
Nominal p-value	0.16071428
FDR q-value	0.33333334
FWER p-Value	0.017

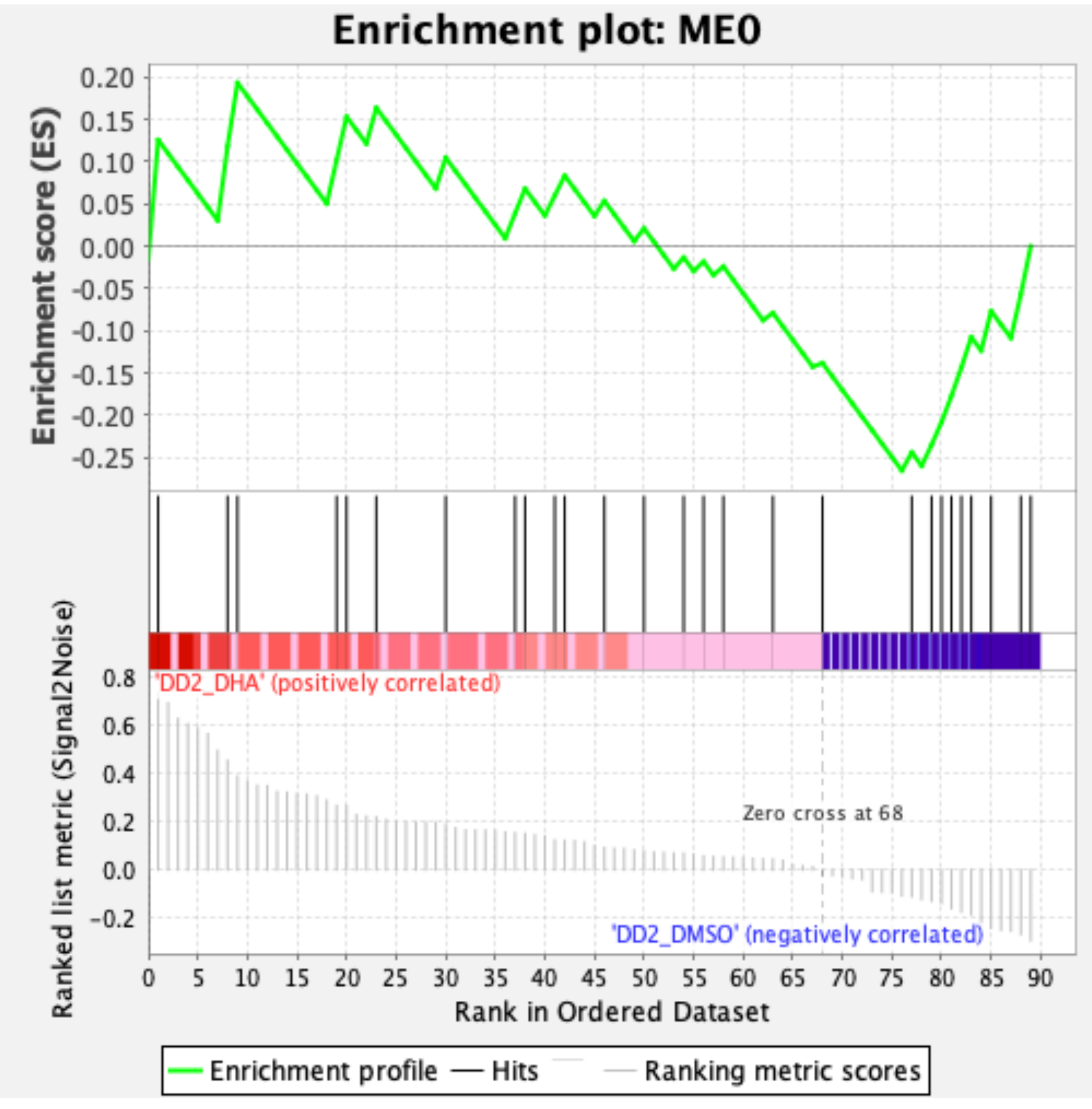

Fig 1: Enrichment plot: ME0
Profile of the Running ES Score & Positions of GeneSet Members on the Rank Ordered List

Table: GSEA details [plain text format]

	SYMBOL	TITLE	RANK IN GENE LIST	RANK METRIC SCORE	RUNNING ES	CORE ENRICHMENT
1	PF3D7_1000800	NA	1	0.730	0.1257	No
2	PF3D7_1000700	NA	8	0.453	0.1182	No
3	PF3D7_0533000	NA	9	0.387	0.1932	No
4	PF3D7_1478700	NA	19	0.265	0.1019	No
5	PF3D7_1477000	NA	20	0.264	0.1531	No
6	PF3D7_1372500	NA	23	0.218	0.1636	No
7	PF3D7_0500700	NA	30	0.187	0.1046	No
8	PF3D7_0601700	NA	37	0.152	0.0390	No
9	PF3D7_0402700	NA	38	0.149	0.0679	No
10	PF3D7_0221500	NA	41	0.122	0.0600	No
11	PF3D7_1129850	NA	42	0.121	0.0834	No
12	PF3D7_1253900	NA	46	0.092	0.0535	No
13	PF3D7_1219200	NA	50	0.078	0.0210	No
14	PF3D7_0500600	NA	54	0.068	-0.0135	No
15	PF3D7_0424300	NA	56	0.057	-0.0183	No
16	PF3D7_0532800	NA	58	0.052	-0.0240	No
17	PF3D7_1478300	NA	63	0.044	-0.0790	No
18	PF3D7_0102100	NA	68	-0.021	-0.1384	No
19	PF3D7_0221100	NA	77	-0.110	-0.2440	Yes
20	PF3D7_1334900	NA	79	-0.129	-0.2349	Yes
21	PF3D7_0425300	NA	80	-0.138	-0.2081	Yes
22	PF3D7_1478200	NA	81	-0.160	-0.1771	Yes
23	PF3D7_1220200	NA	82	-0.173	-0.1435	Yes
24	PF3D7_1463100	NA	83	-0.185	-0.1076	Yes
25	PF3D7_0425250	NA	85	-0.241	-0.0768	Yes
26	PF3D7_1478500	NA	88	-0.267	-0.0568	Yes
27	PF3D7_0400200	NA	89	-0.293	0.0000	Yes

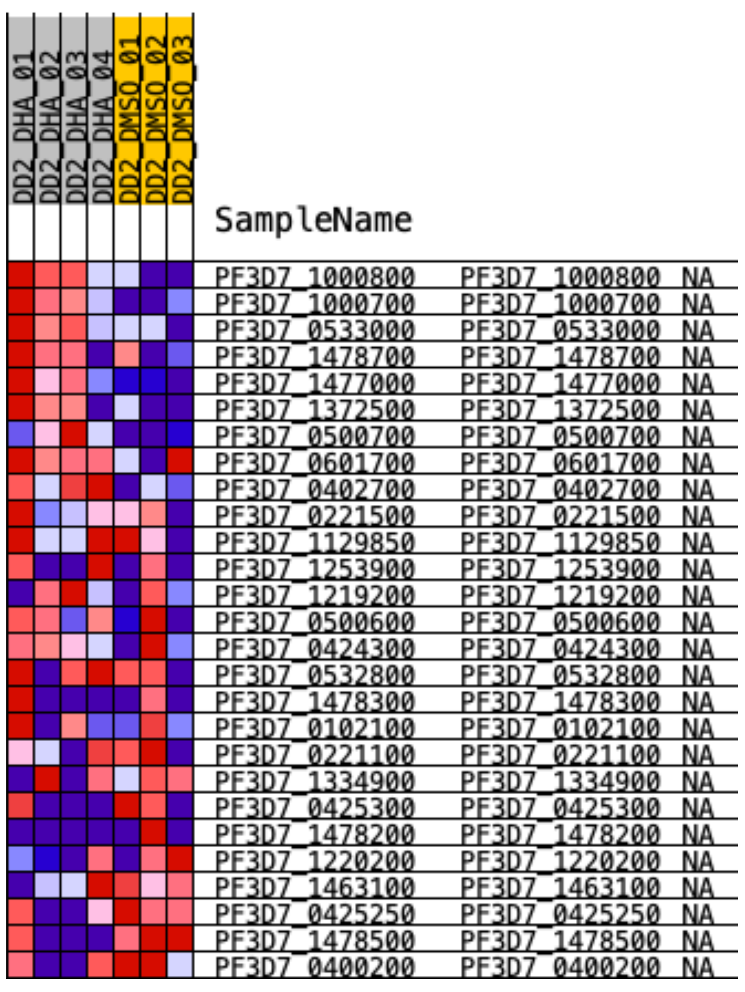

Fig 2: ME0
Blue-Pink O' Gram in the Space of the Analyzed GeneSet

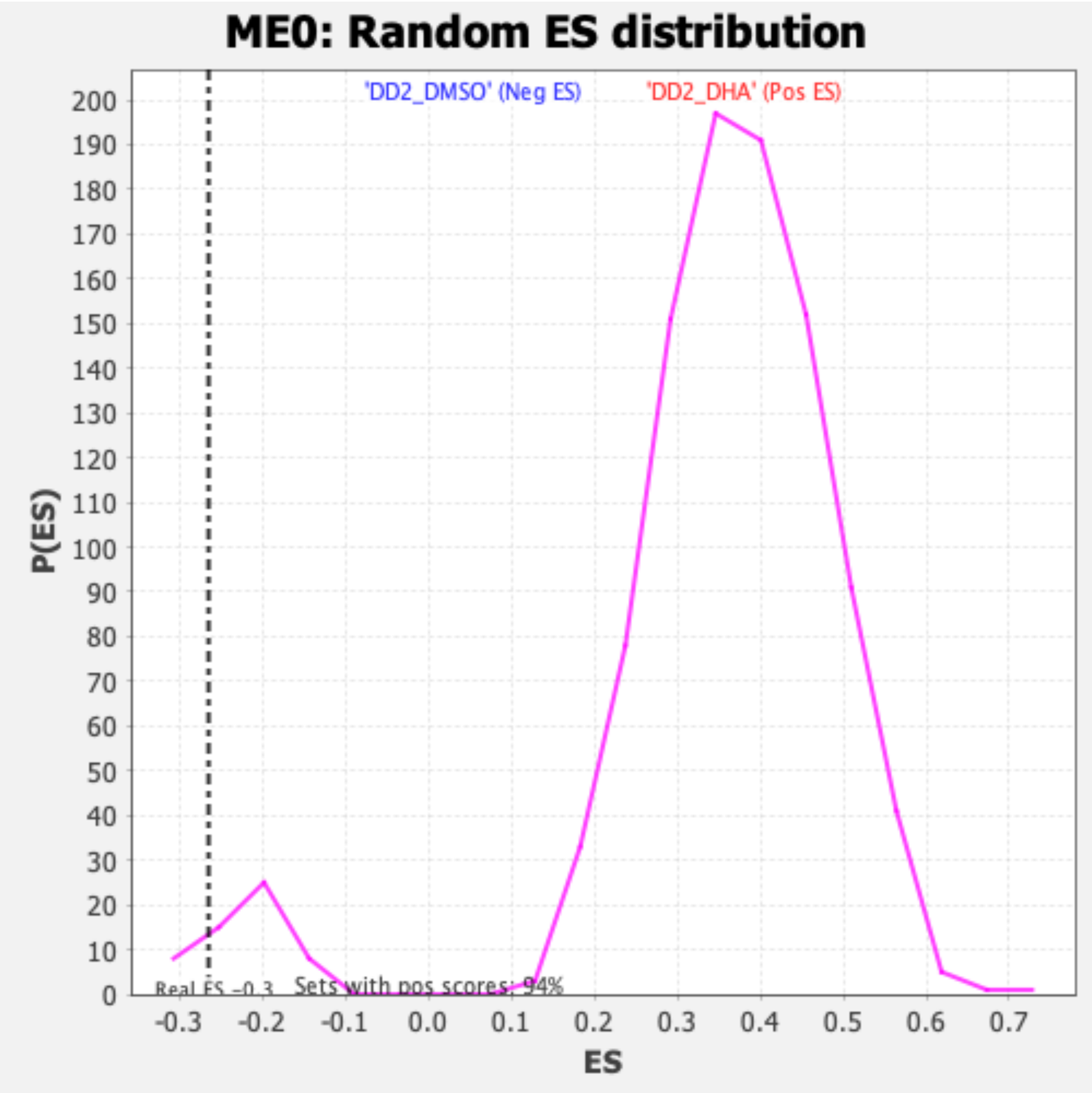

Fig 3: ME0: Random ES distribution
Gene set null distribution of ES for ME0
