# Supplementary material for: Transcriptome-module phenotype association study implicates extracellular vesicles biogenesis in Plasmodium falciparum artemisinin resistance
Source: Front Cell Infect Microbiol. 2022 Aug 19;12:886728. doi: 10.3389/fcimb.2022.886728 (PMC9437462; doi:10.3389/fcimb.2022.886728)
Supplement: Supplementary file 1 [file DataSheet_1.zip › Supplementary_files/Supplementary_Data_14.pdf]

Table: GSEA Results Summary

Dataset	Expression_dataset_dataset_collapsed_to_symbols.PhenotypeData.cls #C580R_DHA_versus_DD2_DHA.PhenotypeData.cls #C580R_DHA_versus_DD2_DHA_repos
Phenotype	PhenotypeData.cls#C580R_DHA_versus_DD2_DHA_repos
Upregulated in class	C580R_DHA
GeneSet	ME7
Enrichment Score (ES)	0.41607258
Normalized Enrichment Score (NES)	1.088161
Nominal p-value	0.3448795
FDR q-value	0.686584
FWER p-Value	0.406

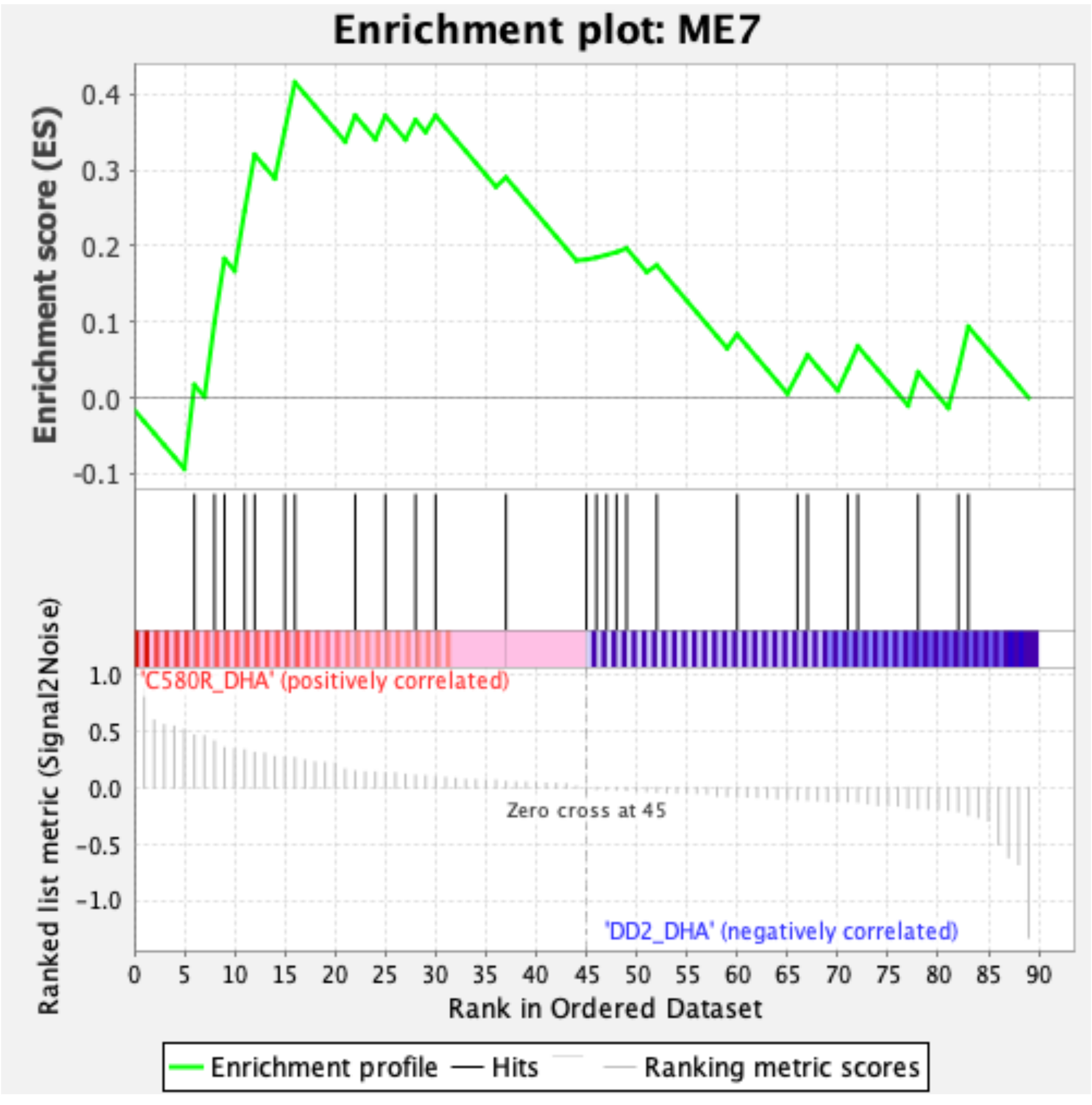

Fig 1: Enrichment plot: ME7
Profile of the Running ES Score & Positions of GeneSet Members on the Rank Ordered List

Table: GSEA details [plain text format]

	SYMBOL	TITLE	RANK IN GENE LIST	RANK METRIC SCORE	RUNNING ES	CORE ENRICHMENT
1	PF3D7_0425000	NA	6	0.464	0.0169	Yes
2	PF3D7_0401500	NA	8	0.411	0.0994	Yes
3	PF3D7_0421500	NA	9	0.352	0.1832	Yes
4	PF3D7_0402800	NA	11	0.332	0.2469	Yes
5	PF3D7_0221900	NA	12	0.309	0.3206	Yes
6	PF3D7_0115150	NA	15	0.268	0.3533	Yes
7	PF3D7_1240200	NA	16	0.263	0.4161	Yes
8	PF3D7_0413400	NA	22	0.144	0.3723	No
9	PF3D7_1219400	NA	25	0.130	0.3720	No
10	PF3D7_0114400	NA	28	0.107	0.3662	No
11	PF3D7_0221300	NA	30	0.091	0.3722	No
12	PF3D7_0221650	NA	37	0.051	0.2906	No
13	PF3D7_1219500	NA	45	-0.005	0.1823	No
14	PF3D7_1240700	NA	46	-0.011	0.1850	No
15	PF3D7_0114300	NA	47	-0.015	0.1885	No
16	PF3D7_0421600	NA	48	-0.015	0.1922	No
17	PF3D7_1478400	NA	49	-0.021	0.1972	No
18	PF3D7_0713300	NA	52	-0.037	0.1747	No
19	PF3D7_1401050	NA	60	-0.077	0.0837	No
20	PF3D7_0632600	NA	66	-0.105	0.0307	No
21	PF3D7_1000900	NA	67	-0.107	0.0564	No
22	PF3D7_0114600	NA	71	-0.121	0.0383	No
23	PF3D7_1480100	NA	72	-0.124	0.0679	No
24	PF3D7_0712500	NA	78	-0.183	0.0335	No
25	PF3D7_1400100	NA	82	-0.208	0.0362	No
26	PF3D7_0302300	NA	83	-0.241	0.0938	No

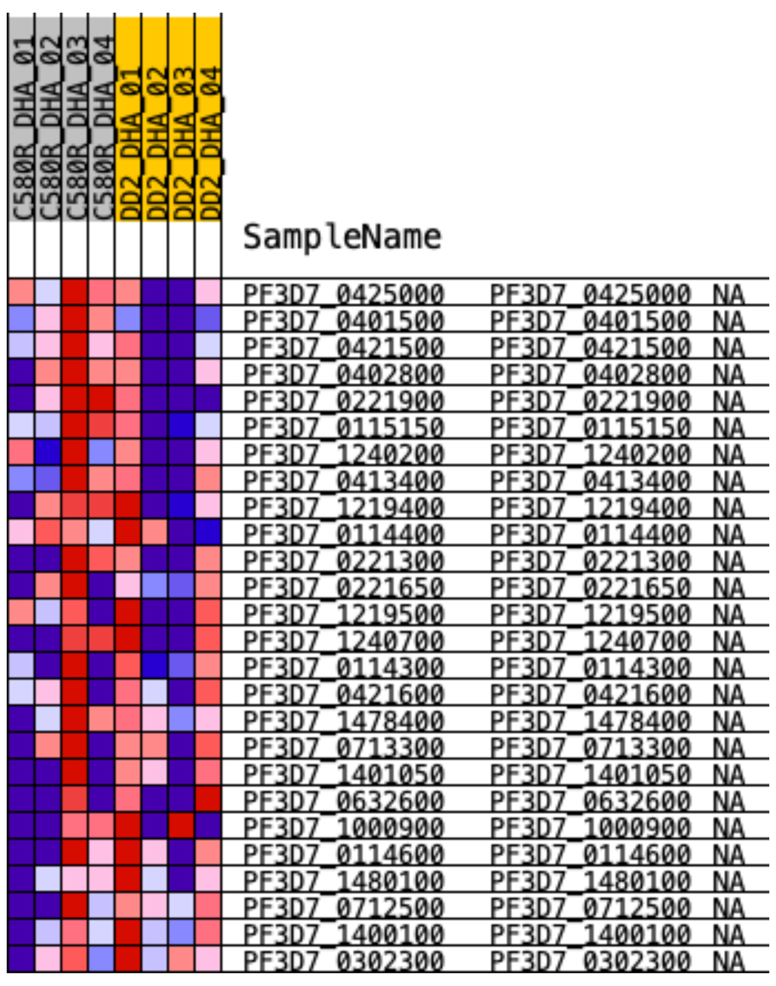

Fig 2: ME7
Blue-Pink O' Gram in the Space of the Analyzed GeneSet

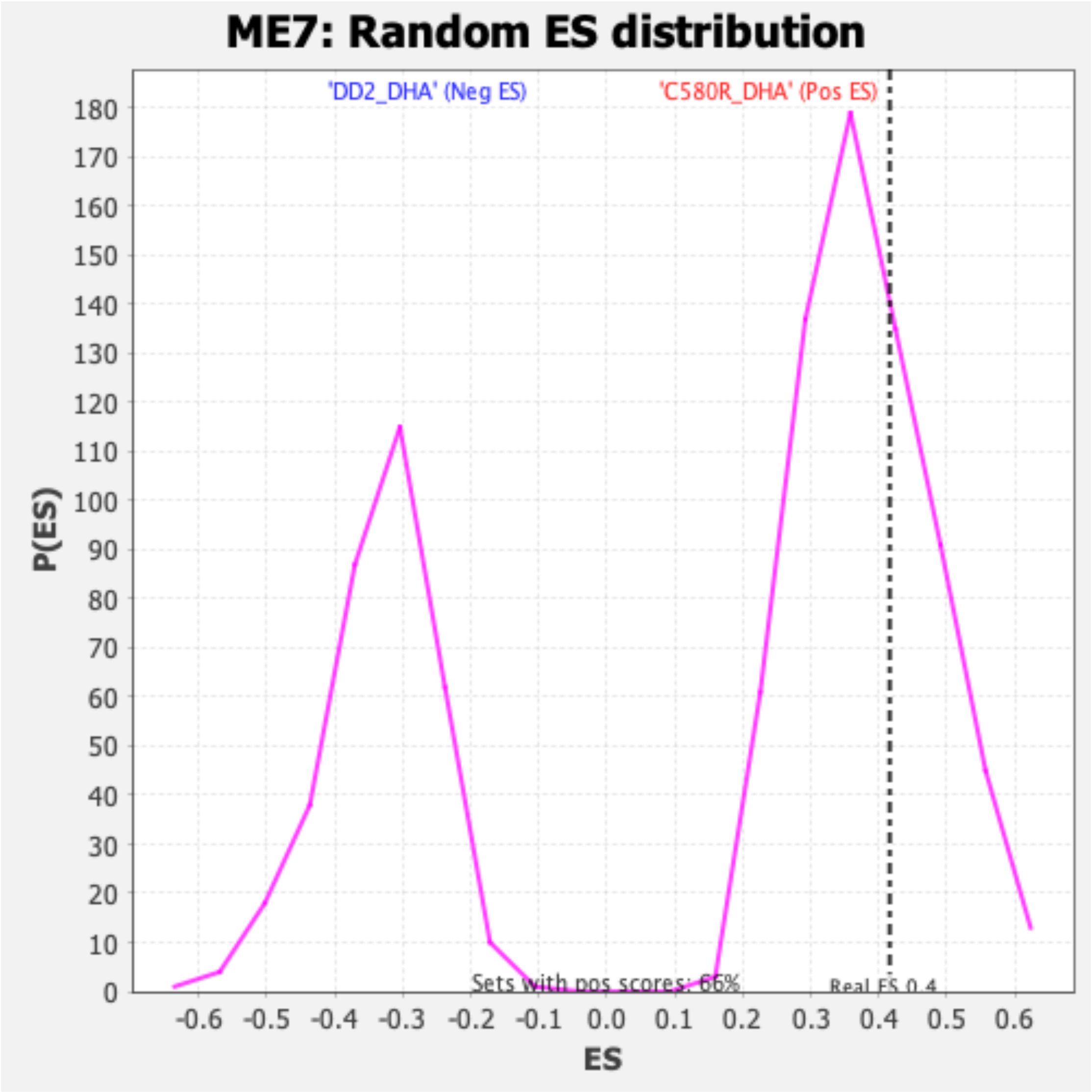

Fig 3: ME7: Random ES distribution
Gene set null distribution of ES for ME7
