# Supplementary material for: Transcriptome-module phenotype association study implicates extracellular vesicles biogenesis in Plasmodium falciparum artemisinin resistance
Source: Front Cell Infect Microbiol. 2022 Aug 19;12:886728. doi: 10.3389/fcimb.2022.886728 (PMC9437462; doi:10.3389/fcimb.2022.886728)
Supplement: Supplementary file 1 [file DataSheet_1.zip › Supplementary_files/Supplementary_Data_15.pdf]

Table: GSEA Results Summary

Dataset	Expression_dataset_dataset_collapsed_to_symbols.PhenotypeData.cls #DD2_DHA_versus_DD2_UNT.PhenotypeData.cls #DD2_DHA_versus_DD2_UNT_repos
Phenotype	PhenotypeData.cls#DD2_DHA_versus_DD2_UNT_repos
Upregulated in class	DD2_UNT
GeneSet	ME7
Enrichment Score (ES)	-0.6388882
Normalized Enrichment Score (NES)	-2.2366247
Nominal p-value	0.0
FDR q-value	0.0
FWER p-Value	0.0

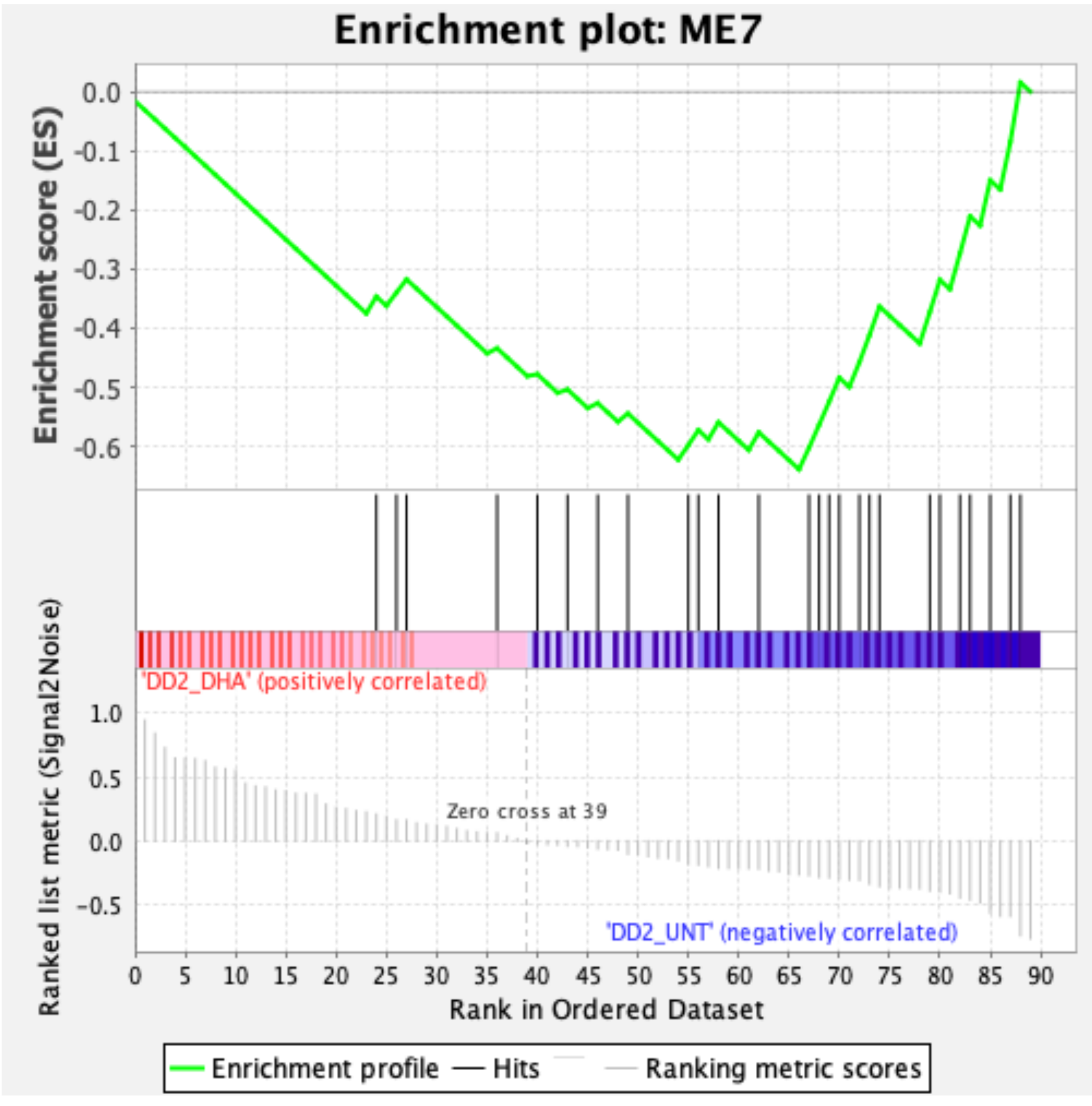

Fig 1: Enrichment plot: ME7
Profile of the Running ES Score & Positions of GeneSet Members on the Rank Ordered List

Table: GSEA details [plain text format]

	SYMBOL	TITLE	RANK IN GENE LIST	RANK METRIC SCORE	RUNNING ES	CORE ENRICHMENT
1	PF3D7_0632600	NA	24	0.208	-0.3466	No
2	PF3D7_1401050	NA	26	0.163	-0.3398	No
3	PF3D7_1000900	NA	27	0.163	-0.3176	No
4	PF3D7_0302300	NA	36	0.061	-0.4342	No
5	PF3D7_0114400	NA	40	-0.022	-0.4780	No
6	PF3D7_0221650	NA	43	-0.039	-0.5039	No
7	PF3D7_0401500	NA	46	-0.060	-0.5270	No
8	PF3D7_0221300	NA	49	-0.101	-0.5445	No
9	PF3D7_0421600	NA	55	-0.181	-0.5979	No
10	PF3D7_0713300	NA	56	-0.185	-0.5726	No
11	PF3D7_0114300	NA	58	-0.211	-0.5593	No
12	PF3D7_1219400	NA	62	-0.218	-0.5764	No
13	PF3D7_1478400	NA	67	-0.268	-0.6022	Yes
14	PF3D7_0114600	NA	68	-0.283	-0.5635	Yes
15	PF3D7_1400100	NA	69	-0.286	-0.5244	Yes
16	PF3D7_0115150	NA	70	-0.296	-0.4839	Yes
17	PF3D7_0712500	NA	72	-0.305	-0.4578	Yes
18	PF3D7_0221900	NA	73	-0.336	-0.4119	Yes
19	PF3D7_1219500	NA	74	-0.354	-0.3635	Yes
20	PF3D7_1480100	NA	79	-0.388	-0.3729	Yes
21	PF3D7_0421500	NA	80	-0.397	-0.3185	Yes
22	PF3D7_1240700	NA	82	-0.443	-0.2736	Yes
23	PF3D7_0425000	NA	83	-0.459	-0.2108	Yes
24	PF3D7_0413400	NA	85	-0.560	-0.1499	Yes
25	PF3D7_1240200	NA	87	-0.587	-0.0852	Yes
26	PF3D7_0402800	NA	88	-0.738	0.0156	Yes

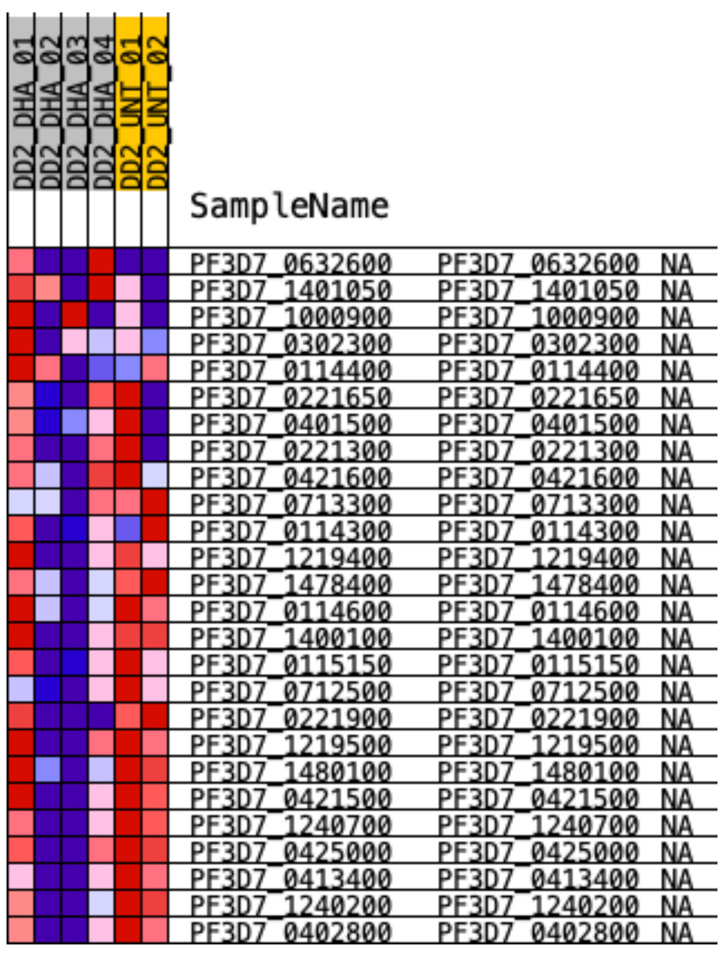

Fig 2: ME7
Blue-Pink O' Gram in the Space of the Analyzed GeneSet

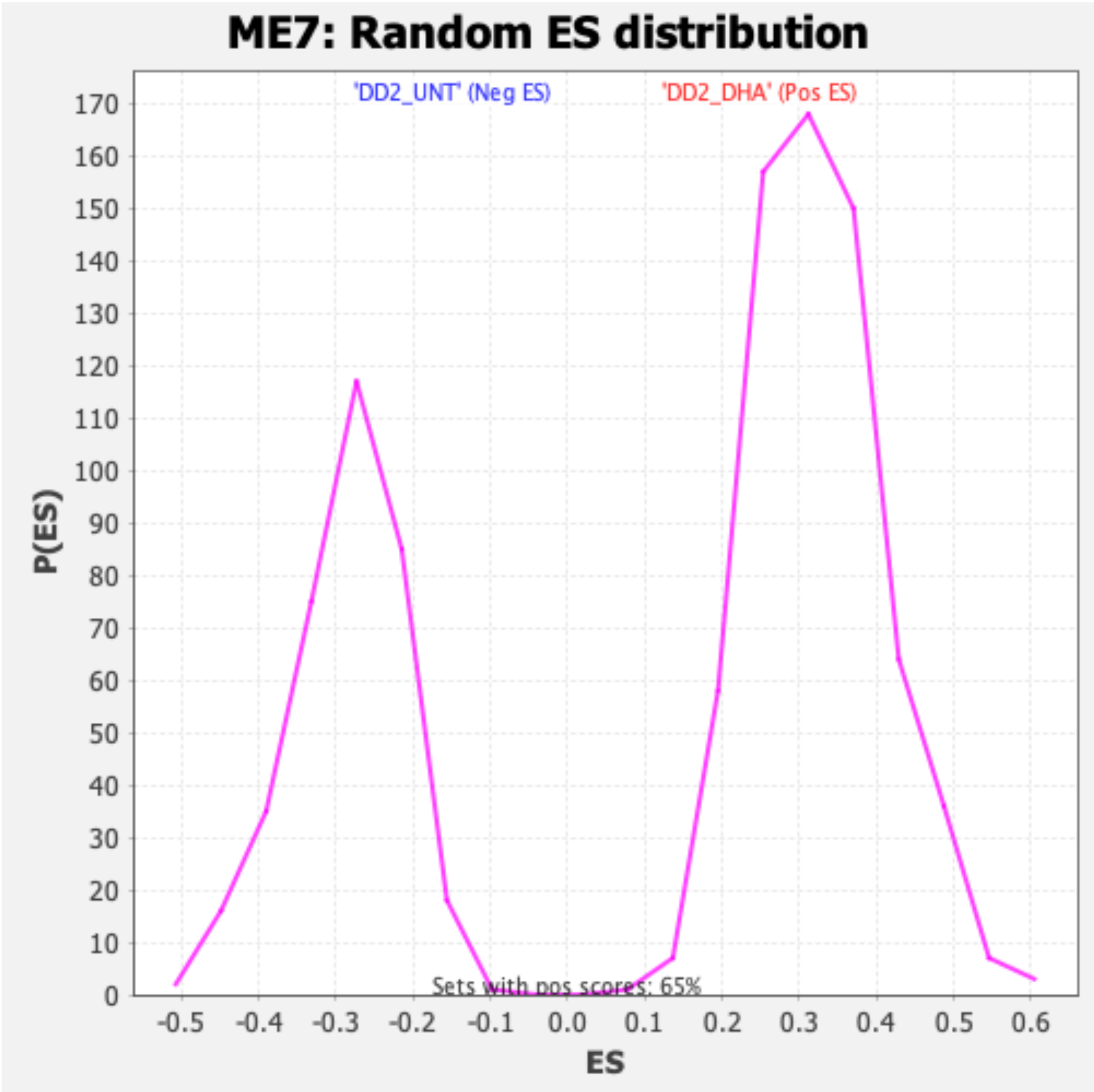

Fig 3: ME7: Random ES distribution
Gene set null distribution of ES for ME7
