# Supplementary material for: Transcriptome-module phenotype association study implicates extracellular vesicles biogenesis in Plasmodium falciparum artemisinin resistance
Source: Front Cell Infect Microbiol. 2022 Aug 19;12:886728. doi: 10.3389/fcimb.2022.886728 (PMC9437462; doi:10.3389/fcimb.2022.886728)
Supplement: Supplementary file 1 [file DataSheet_1.zip › Supplementary_files/Supplementary_Data_11.pdf]

Table: GSEA Results Summary

Dataset	Expression_dataset_dataset_collapsed_to_symbols.PhenotypeData.cls #R539T_DHA_versus_DD2_DHA.PhenotypeData.cls #R539T_DHA_versus_DD2_DHA_repos
Phenotype	PhenotypeData.cls#R539T_DHA_versus_DD2_DHA_repos
Upregulated in class	R539T_DHA
GeneSet	ME7
Enrichment Score (ES)	0.25544685
Normalized Enrichment Score (NES)	0.6829786
Nominal p-value	0.9445585
FDR q-value	0.9447514
FWER p-Value	0.7

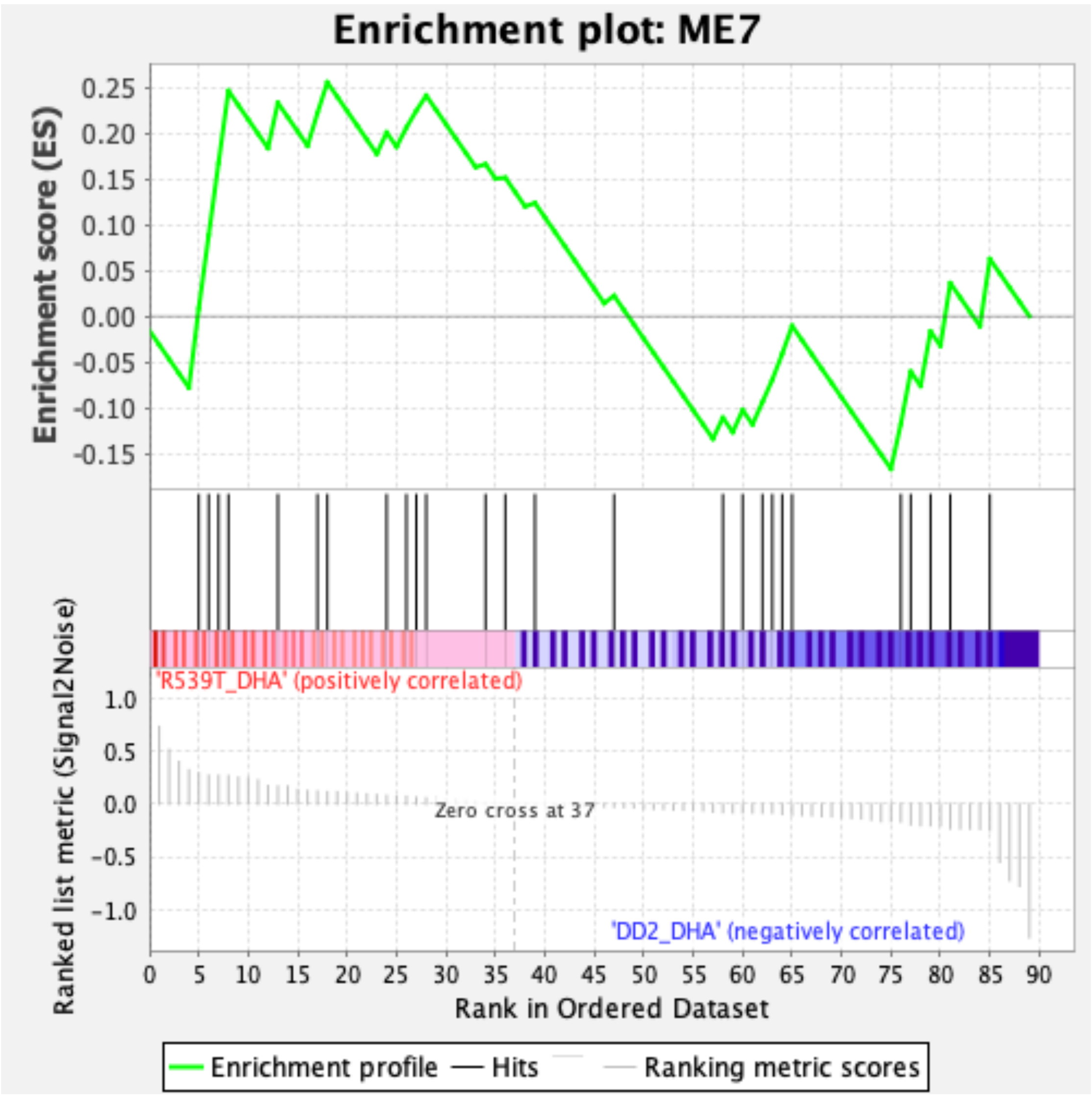

Fig 1: Enrichment plot: ME7
Profile of the Running ES Score & Positions of GeneSet Members on the Rank Ordered List

Table: GSEA details [plain text format]

	SYMBOL	TITLE	RANK IN GENE LIST	RANK METRIC SCORE	RUNNING ES	CORE ENRICHMENT
1	PF3D7_0221900	NA	5	0.295	0.0086	Yes
2	PF3D7_0115150	NA	6	0.270	0.0882	Yes
3	PF3D7_0402800	NA	7	0.269	0.1675	Yes
4	PF3D7_0401500	NA	8	0.267	0.2461	Yes
5	PF3D7_0425000	NA	13	0.168	0.2331	Yes
6	PF3D7_0413400	NA	17	0.121	0.2218	Yes
7	PF3D7_1240200	NA	18	0.114	0.2554	Yes
8	PF3D7_0421500	NA	24	0.079	0.2007	No
9	PF3D7_1400100	NA	26	0.072	0.2062	No
10	PF3D7_1401050	NA	27	0.062	0.2245	No
11	PF3D7_1480100	NA	28	0.056	0.2410	No
12	PF3D7_1219400	NA	34	0.011	0.1660	No
13	PF3D7_1219500	NA	36	0.003	0.1512	No
14	PF3D7_0114400	NA	39	-0.012	0.1236	No
15	PF3D7_0632600	NA	47	-0.027	0.0223	No
16	PF3D7_0114600	NA	58	-0.078	-0.1110	No
17	PF3D7_0221300	NA	60	-0.082	-0.1026	No
18	PF3D7_0114300	NA	62	-0.083	-0.0939	No
19	PF3D7_0713300	NA	63	-0.083	-0.0694	No
20	PF3D7_1240700	NA	64	-0.096	-0.0412	No
21	PF3D7_0712500	NA	65	-0.104	-0.0105	No
22	PF3D7_1478400	NA	76	-0.167	-0.1176	No
23	PF3D7_0221650	NA	77	-0.194	-0.0606	No
24	PF3D7_1000900	NA	79	-0.202	-0.0167	No
25	PF3D7_0302300	NA	81	-0.232	0.0360	No
26	PF3D7_0421600	NA	85	-0.249	0.0625	No

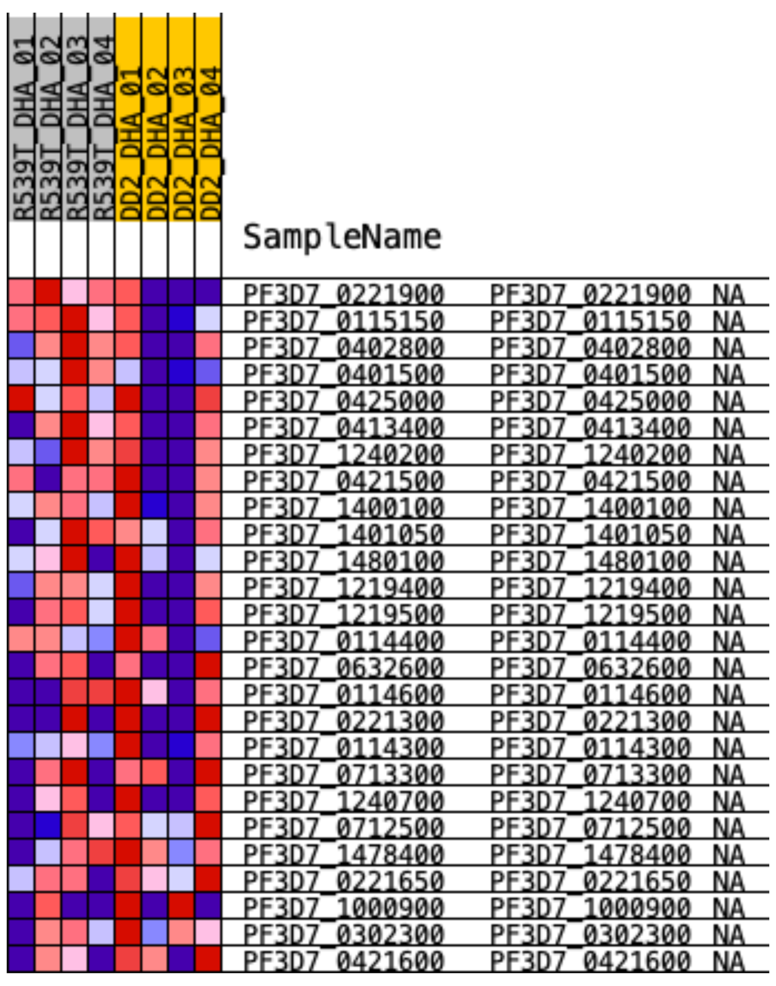

Fig 2: ME7
Blue-Pink O' Gram in the Space of the Analyzed GeneSet

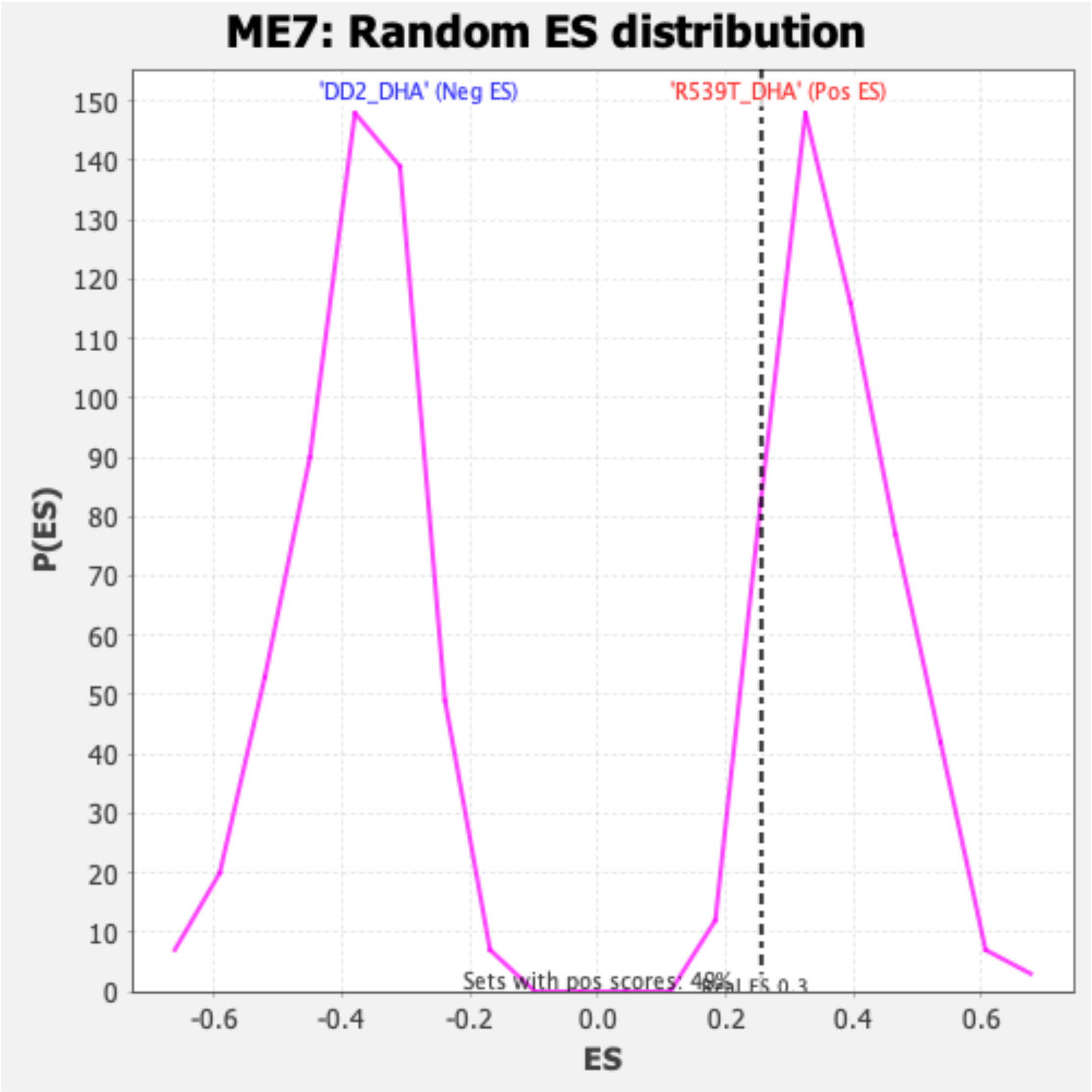

Fig 3: ME7: Random ES distribution
Gene set null distribution of ES for ME7
