# Supplementary material for: Transcriptome-module phenotype association study implicates extracellular vesicles biogenesis in Plasmodium falciparum artemisinin resistance
Source: Front Cell Infect Microbiol. 2022 Aug 19;12:886728. doi: 10.3389/fcimb.2022.886728 (PMC9437462; doi:10.3389/fcimb.2022.886728)
Supplement: Supplementary file 1 [file DataSheet_1.zip › Supplementary_files/Supplementary_Data_10.pdf]

Table: GSEA Results Summary

Dataset	Expression_dataset_dataset_collapsed_to_symbols.PhenotypeData.cls<br>#R539T_DHA_versus_R539T_DMSO.PhenotypeData.cls<br>#R539T_DHA_versus_R539T_DMSO_repos
Phenotype	PhenotypeData.cls#R539T_DHA_versus_R539T_DMSO_repos
Upregulated in class	R539T_DMSO
GeneSet	ME7
Enrichment Score (ES)	-0.31506842
Normalized Enrichment Score (NES)	-1.141659
Nominal p-value	0.19767442
FDR q-value	0.43728814
FWER p-Value	0.066

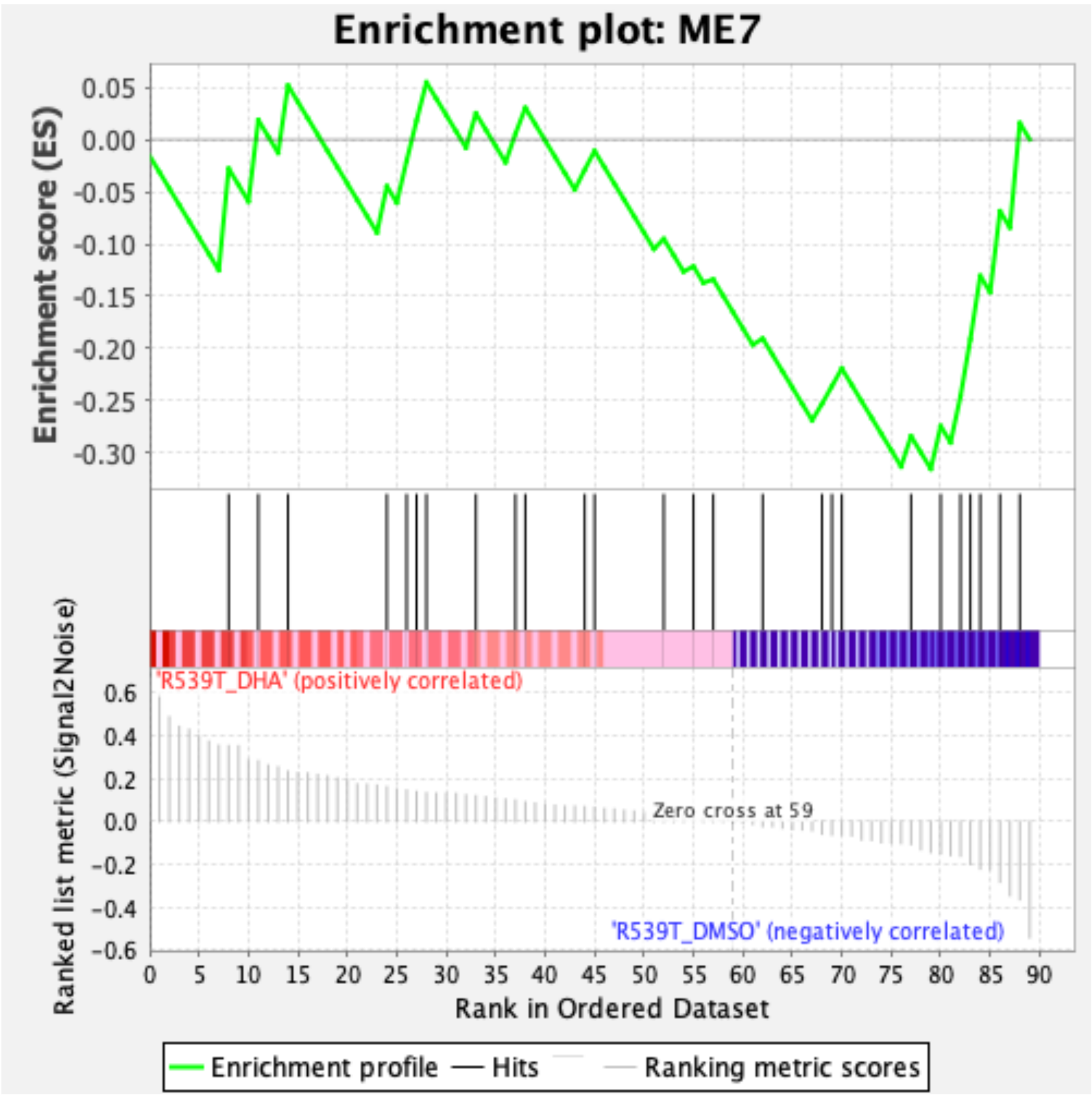

Fig 1: Enrichment plot: ME7
Profile of the Running ES Score & Positions of GeneSet Members on the Rank Ordered List

Table: GSEA details [plain text format]

	SYMBOL	TITLE	RANK IN GENE LIST	RANK METRIC SCORE	RUNNING ES	CORE ENRICHMENT
1	PF3D7_1240200	NA	8	0.353	-0.0279	No
2	PF3D7_1480100	NA	11	0.282	0.0185	No
3	PF3D7_1400100	NA	14	0.234	0.0516	No
4	PF3D7_0115150	NA	24	0.161	-0.0447	No
5	PF3D7_0425000	NA	26	0.146	-0.0203	No
6	PF3D7_0302300	NA	27	0.139	0.0179	No
7	PF3D7_1219500	NA	28	0.133	0.0546	No
8	PF3D7_0413400	NA	33	0.119	0.0249	No
9	PF3D7_0114300	NA	37	0.099	0.0054	No
10	PF3D7_0402800	NA	38	0.091	0.0303	No
11	PF3D7_1240700	NA	44	0.068	-0.0291	No
12	PF3D7_1401050	NA	45	0.066	-0.0110	No
13	PF3D7_0401500	NA	52	0.035	-0.0951	No
14	PF3D7_0632600	NA	55	0.018	-0.1215	No
15	PF3D7_0114400	NA	57	0.012	-0.1339	No
16	PF3D7_0221300	NA	62	-0.022	-0.1904	No
17	PF3D7_1478400	NA	68	-0.057	-0.2528	No
18	PF3D7_0221900	NA	69	-0.061	-0.2360	No
19	PF3D7_1219400	NA	70	-0.062	-0.2191	No
20	PF3D7_0712500	NA	77	-0.105	-0.2838	Yes
21	PF3D7_0221650	NA	80	-0.149	-0.2742	Yes
22	PF3D7_0114600	NA	82	-0.160	-0.2458	Yes
23	PF3D7_1000900	NA	83	-0.199	-0.1910	Yes
24	PF3D7_0421500	NA	84	-0.220	-0.1305	Yes
25	PF3D7_0713300	NA	86	-0.282	-0.0687	Yes
26	PF3D7_0421600	NA	88	-0.363	0.0156	Yes

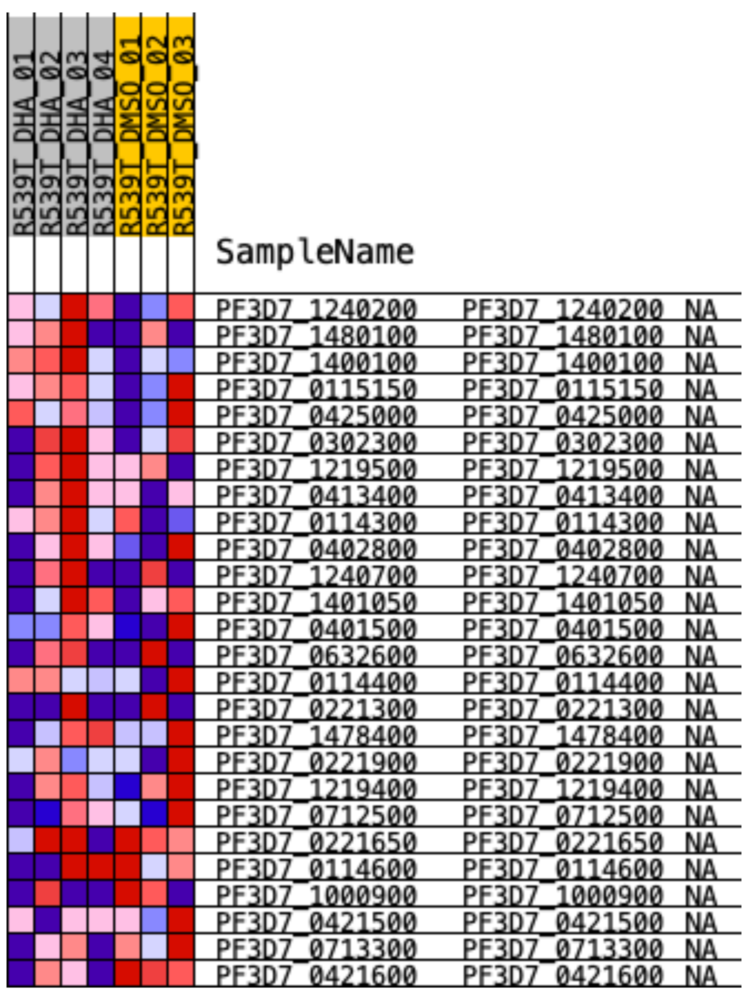

Fig 2: ME7
Blue-Pink O' Gram in the Space of the Analyzed GeneSet

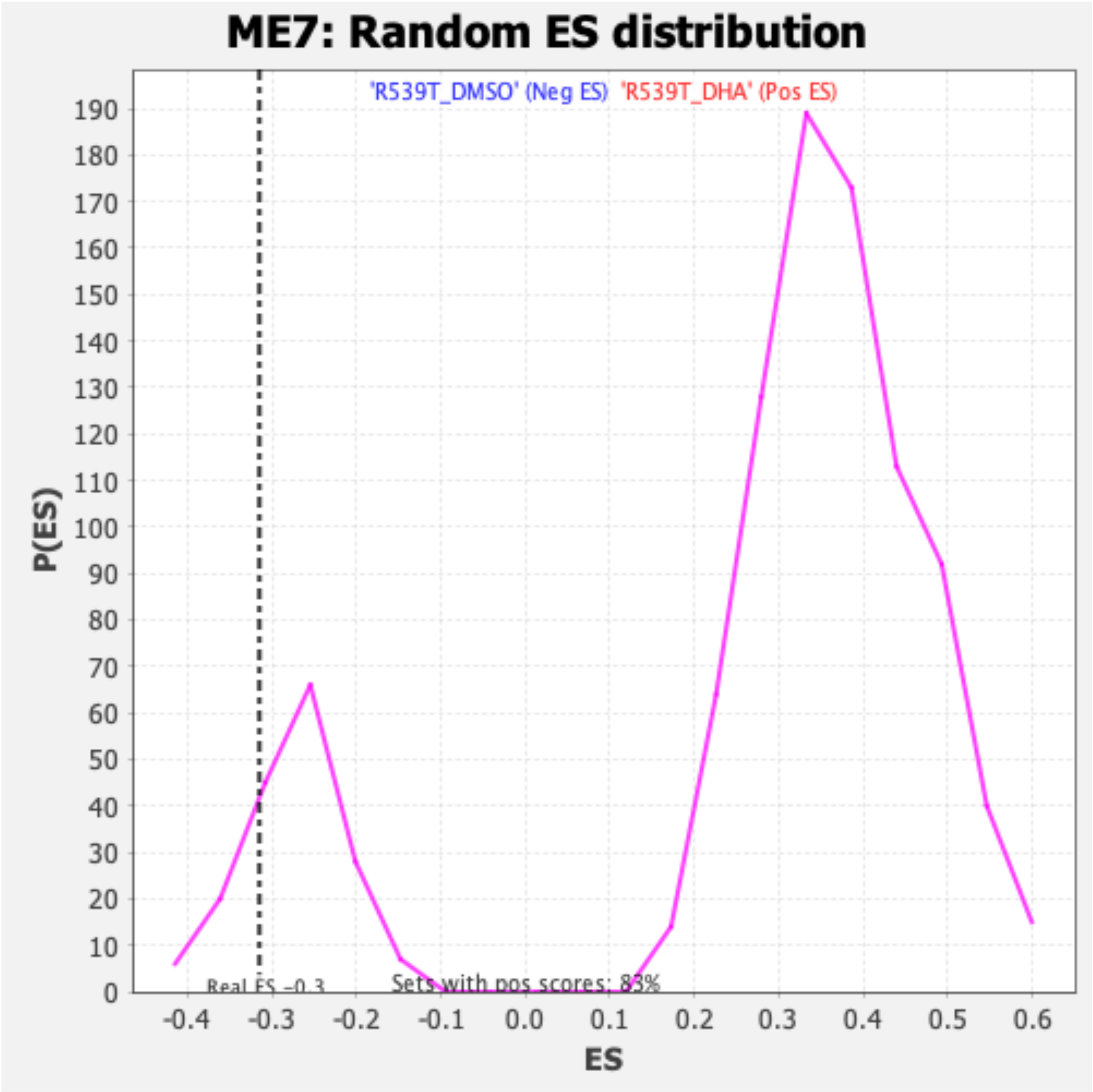

Fig 3: ME7: Random ES distribution
Gene set null distribution of ES for ME7
