# Supplementary material for: Transcriptome-module phenotype association study implicates extracellular vesicles biogenesis in Plasmodium falciparum artemisinin resistance
Source: Front Cell Infect Microbiol. 2022 Aug 19;12:886728. doi: 10.3389/fcimb.2022.886728 (PMC9437462; doi:10.3389/fcimb.2022.886728)
Supplement: Supplementary file 1 [file DataSheet_1.zip › Supplementary_files/Supplementary_Data_12.pdf]

Table: GSEA Results Summary

Dataset	Expression_dataset_dataset_collapsed_to_symbols.PhenotypeData.cls #C580R_DHA_versus_C580R_UNT.PhenotypeData.cls #C580R_DHA_versus_C580R_UNT_repos
Phenotype	PhenotypeData.cls#C580R_DHA_versus_C580R_UNT_repos
Upregulated in class	C580R_UNT
GeneSet	ME7
Enrichment Score (ES)	-0.8752158
Normalized Enrichment Score (NES)	-2.4335837
Nominal p-value	0.0
FDR q-value	0.0
FWER p-Value	0.0

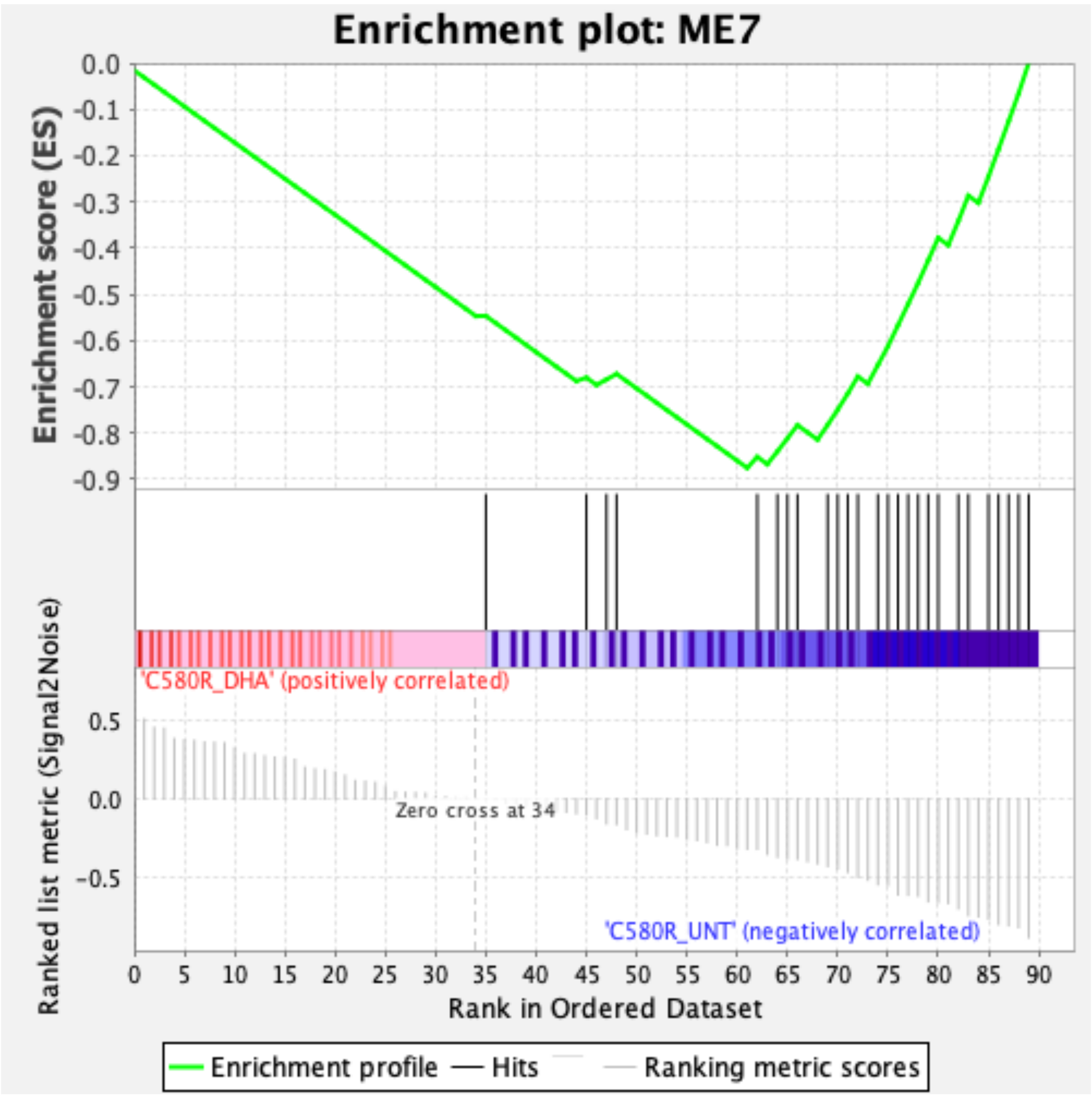

Fig 1: Enrichment plot: ME7
Profile of the Running ES Score & Positions of GeneSet Members on the Rank Ordered List

Table: GSEA details [plain text format]

	SYMBOL	TITLE	RANK IN GENE LIST	RANK METRIC SCORE	RUNNING ES	CORE ENRICHMENT
1	PF3D7_0114400	NA	35	-0.001	-0.5468	No
2	PF3D7_0221300	NA	45	-0.097	-0.6801	No
3	PF3D7_0401500	NA	47	-0.156	-0.6841	No
4	PF3D7_1219500	NA	48	-0.160	-0.6721	No
5	PF3D7_1000900	NA	62	-0.318	-0.8515	Yes
6	PF3D7_0421500	NA	64	-0.368	-0.8396	Yes
7	PF3D7_0114300	NA	65	-0.379	-0.8113	Yes
8	PF3D7_1240200	NA	66	-0.381	-0.7829	Yes
9	PF3D7_0425000	NA	69	-0.427	-0.7822	Yes
10	PF3D7_0221900	NA	70	-0.442	-0.7493	Yes
11	PF3D7_0115150	NA	71	-0.464	-0.7146	Yes
12	PF3D7_0302300	NA	72	-0.495	-0.6777	Yes
13	PF3D7_0221650	NA	74	-0.542	-0.6528	Yes
14	PF3D7_0632600	NA	75	-0.545	-0.6121	Yes
15	PF3D7_0713300	NA	76	-0.607	-0.5668	Yes
16	PF3D7_1480100	NA	77	-0.609	-0.5213	Yes
17	PF3D7_1219400	NA	78	-0.615	-0.4754	Yes
18	PF3D7_1240700	NA	79	-0.650	-0.4269	Yes
19	PF3D7_1400100	NA	80	-0.655	-0.3780	Yes
20	PF3D7_0114600	NA	82	-0.695	-0.3417	Yes
21	PF3D7_0413400	NA	83	-0.738	-0.2867	Yes
22	PF3D7_1478400	NA	85	-0.761	-0.2455	Yes
23	PF3D7_0402800	NA	86	-0.794	-0.1862	Yes
24	PF3D7_0421600	NA	87	-0.802	-0.1263	Yes
25	PF3D7_0712500	NA	88	-0.814	-0.0656	Yes
26	PF3D7_1401050	NA	89	-0.878	-0.0000	Yes

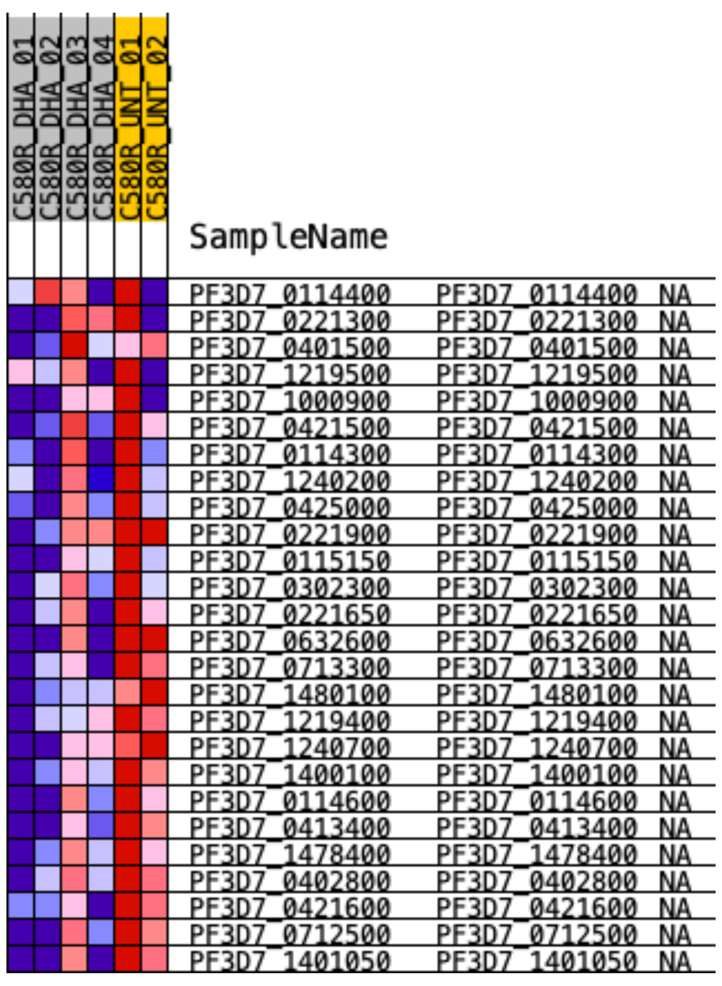

Fig 2: ME7
Blue-Pink O' Gram in the Space of the Analyzed GeneSet

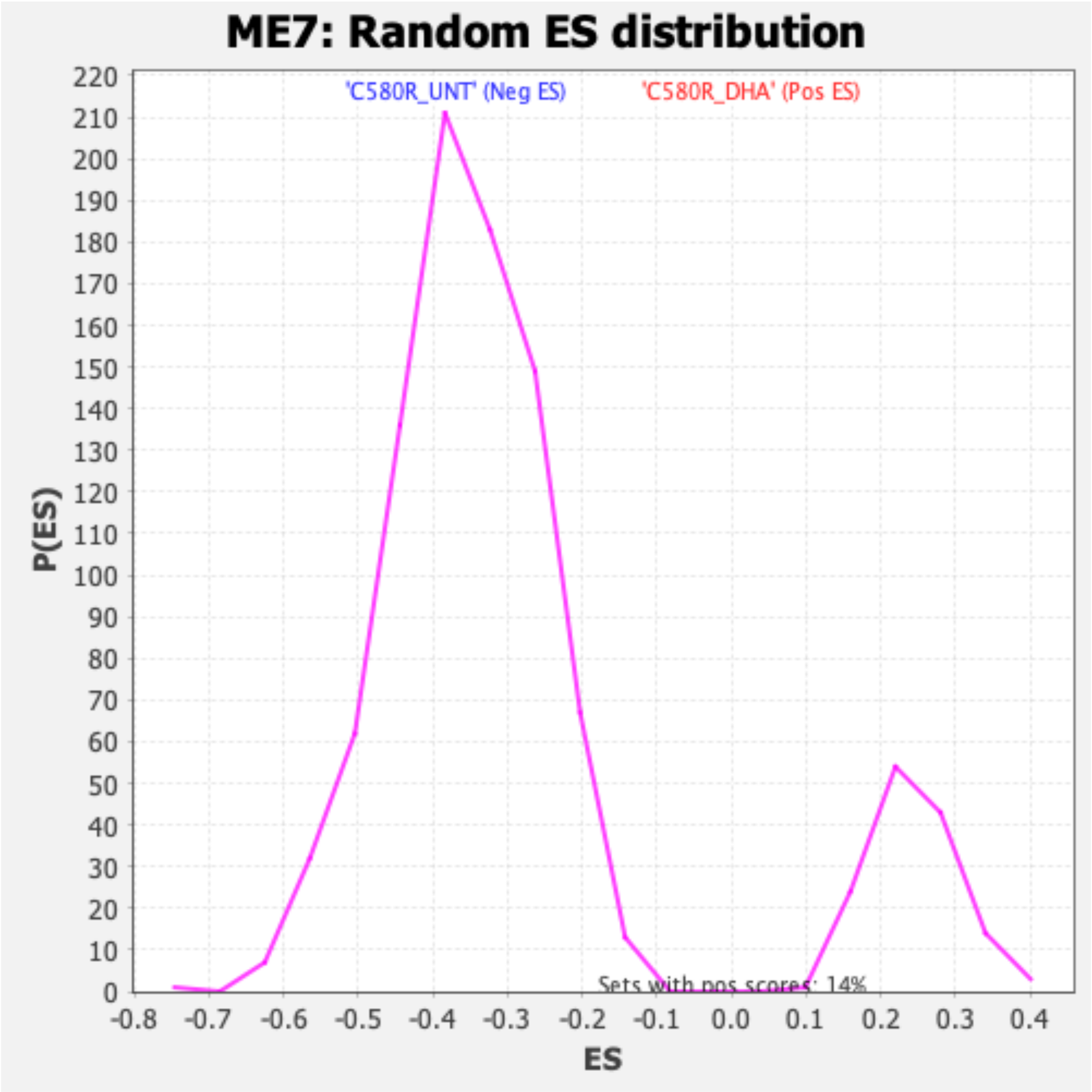

Fig 3: ME7: Random ES distribution
Gene set null distribution of ES for ME7
