# Supplementary material for: Transcriptome-module phenotype association study implicates extracellular vesicles biogenesis in Plasmodium falciparum artemisinin resistance
Source: Front Cell Infect Microbiol. 2022 Aug 19;12:886728. doi: 10.3389/fcimb.2022.886728 (PMC9437462; doi:10.3389/fcimb.2022.886728)
Supplement: Supplementary file 1 [file DataSheet_1.zip › Supplementary_files/Supplementary_Data_13.pdf]

Table: GSEA Results Summary

Dataset	Expression_dataset_dataset_collapsed_to_symbols.PhenotypeData.cls #C580R_DHA_versus_C580R_DMSO.PhenotypeData.cls #C580R_DHA_versus_C580R_DMSO_repos
Phenotype	PhenotypeData.cls#C580R_DHA_versus_C580R_DMSO_repos
Upregulated in class	C580R_DHA
GeneSet	ME7
Enrichment Score (ES)	0.5312583
Normalized Enrichment Score (NES)	1.2448145
Nominal p-value	0.120080724
FDR q-value	0.253
FWER p-Value	0.237

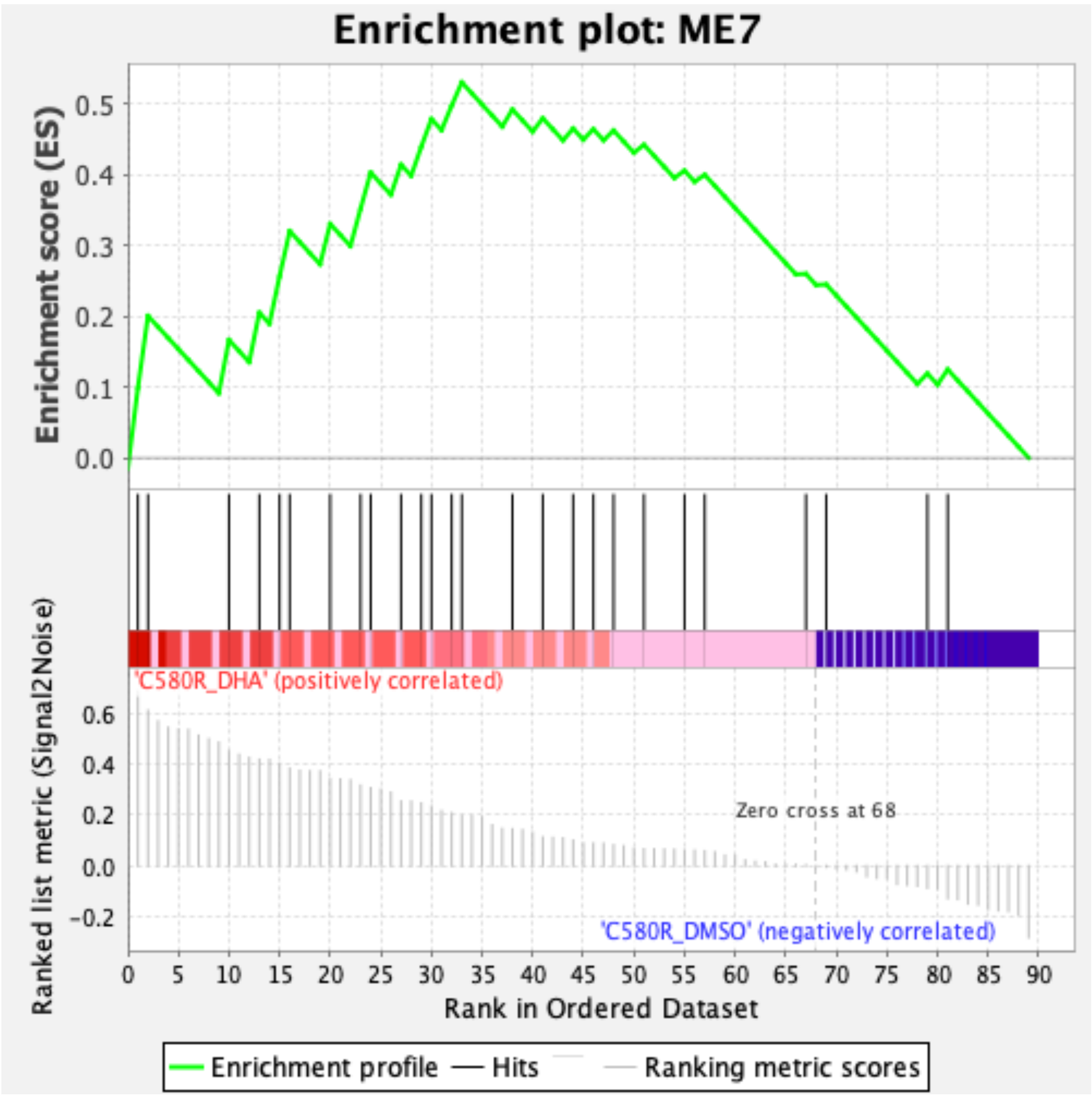

Fig 1: Enrichment plot: ME7
Profile of the Running ES Score & Positions of GeneSet Members on the Rank Ordered List

Table: GSEA details ***[plain text format]***

	SYMBOL	TITLE	RANK IN GENE LIST	RANK METRIC SCORE	RUNNING ES	CORE ENRICHMENT
1	PF3D7_0401500	NA	1	0.685	0.0988	Yes
2	PF3D7_0115150	NA	2	0.610	0.2008	Yes
3	PF3D7_1478400	NA	10	0.450	0.1667	Yes
4	PF3D7_0421500	NA	13	0.418	0.2053	Yes
5	PF3D7_0221650	NA	15	0.402	0.2570	Yes
6	PF3D7_0114400	NA	16	0.382	0.3208	Yes
7	PF3D7_0402800	NA	20	0.341	0.3309	Yes
8	PF3D7_0221900	NA	23	0.316	0.3525	Yes
9	PF3D7_1240200	NA	24	0.306	0.4036	Yes
10	PF3D7_1219400	NA	27	0.254	0.4148	Yes
11	PF3D7_0221300	NA	29	0.246	0.4403	Yes
12	PF3D7_1219500	NA	30	0.234	0.4794	Yes
13	PF3D7_1000900	NA	32	0.207	0.4984	Yes
14	PF3D7_0413400	NA	33	0.197	0.5313	Yes
15	PF3D7_0302300	NA	38	0.144	0.4929	No
16	PF3D7_0114300	NA	41	0.112	0.4804	No
17	PF3D7_1240700	NA	44	0.100	0.4659	No
18	PF3D7_0114600	NA	46	0.088	0.4650	No
19	PF3D7_1480100	NA	48	0.082	0.4631	No
20	PF3D7_0712500	NA	51	0.066	0.4429	No
21	PF3D7_0425000	NA	55	0.062	0.4064	No
22	PF3D7_1401050	NA	57	0.058	0.4004	No
23	PF3D7_1400100	NA	67	0.003	0.2602	No
24	PF3D7_0632600	NA	69	-0.004	0.2453	No
25	PF3D7_0421600	NA	79	-0.087	0.1192	No
26	PF3D7_0713300	NA	81	-0.128	0.1250	No

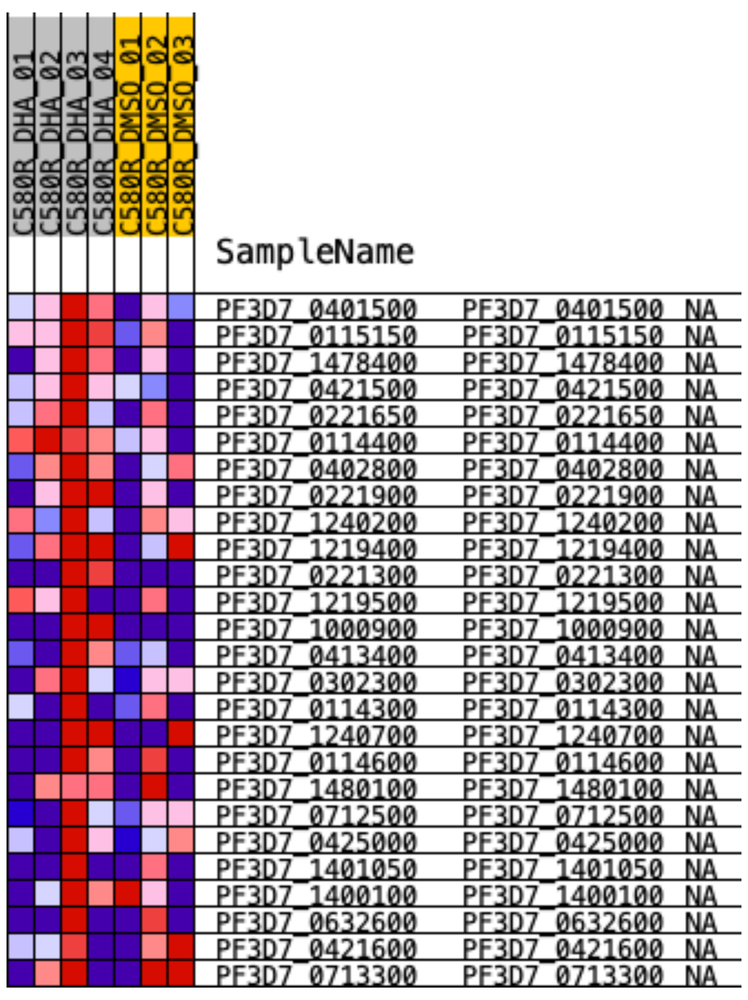

Fig 2: ME7
Blue-Pink O' Gram in the Space of the Analyzed GeneSet

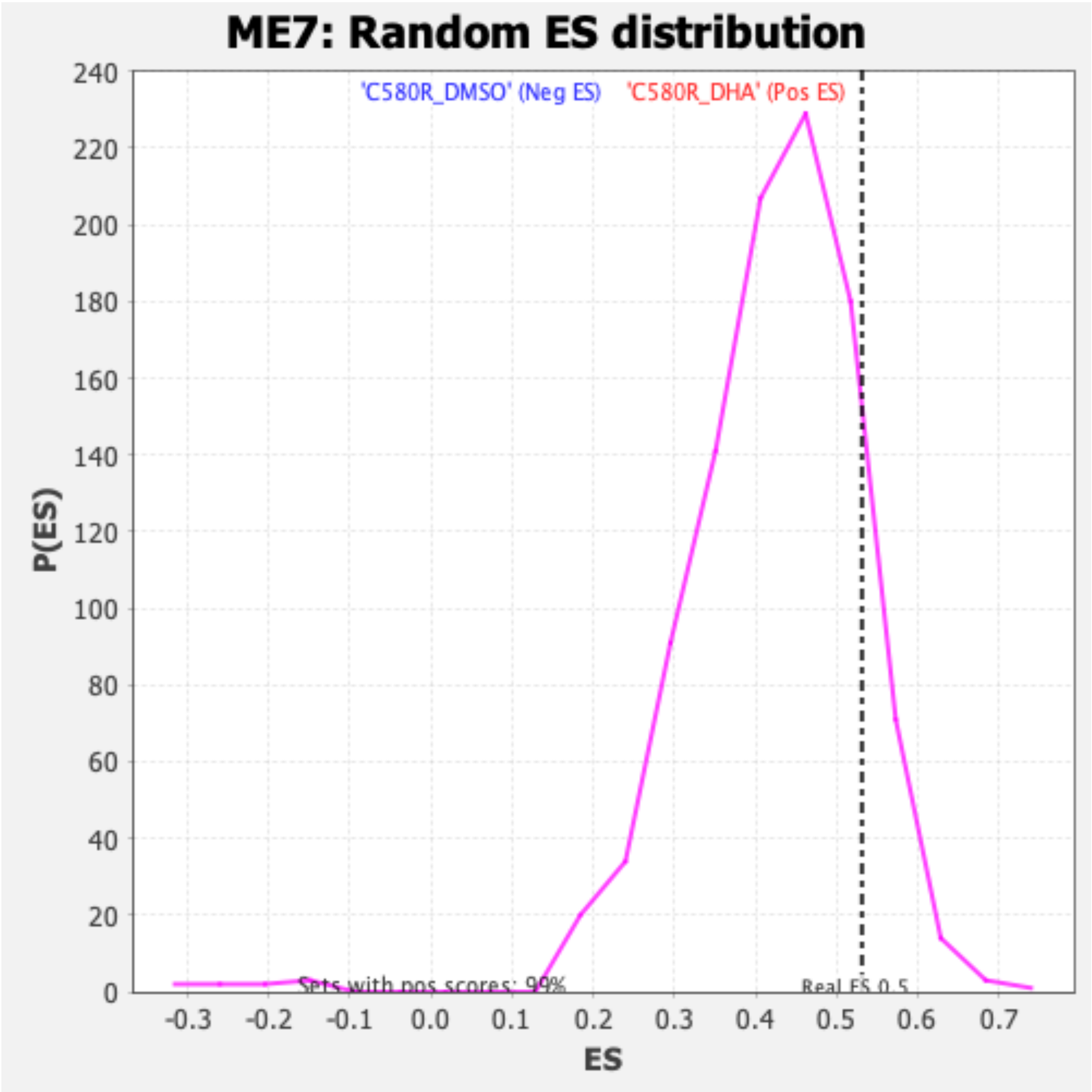

Fig 3: ME7: Random ES distribution
Gene set null distribution of ES for ME7
