# Supplementary material for: Transcriptome-module phenotype association study implicates extracellular vesicles biogenesis in Plasmodium falciparum artemisinin resistance
Source: Front Cell Infect Microbiol. 2022 Aug 19;12:886728. doi: 10.3389/fcimb.2022.886728 (PMC9437462; doi:10.3389/fcimb.2022.886728)
Supplement: Supplementary file 1 [file DataSheet_1.zip › Supplementary_files/Supplementary Figure_1.pdf]

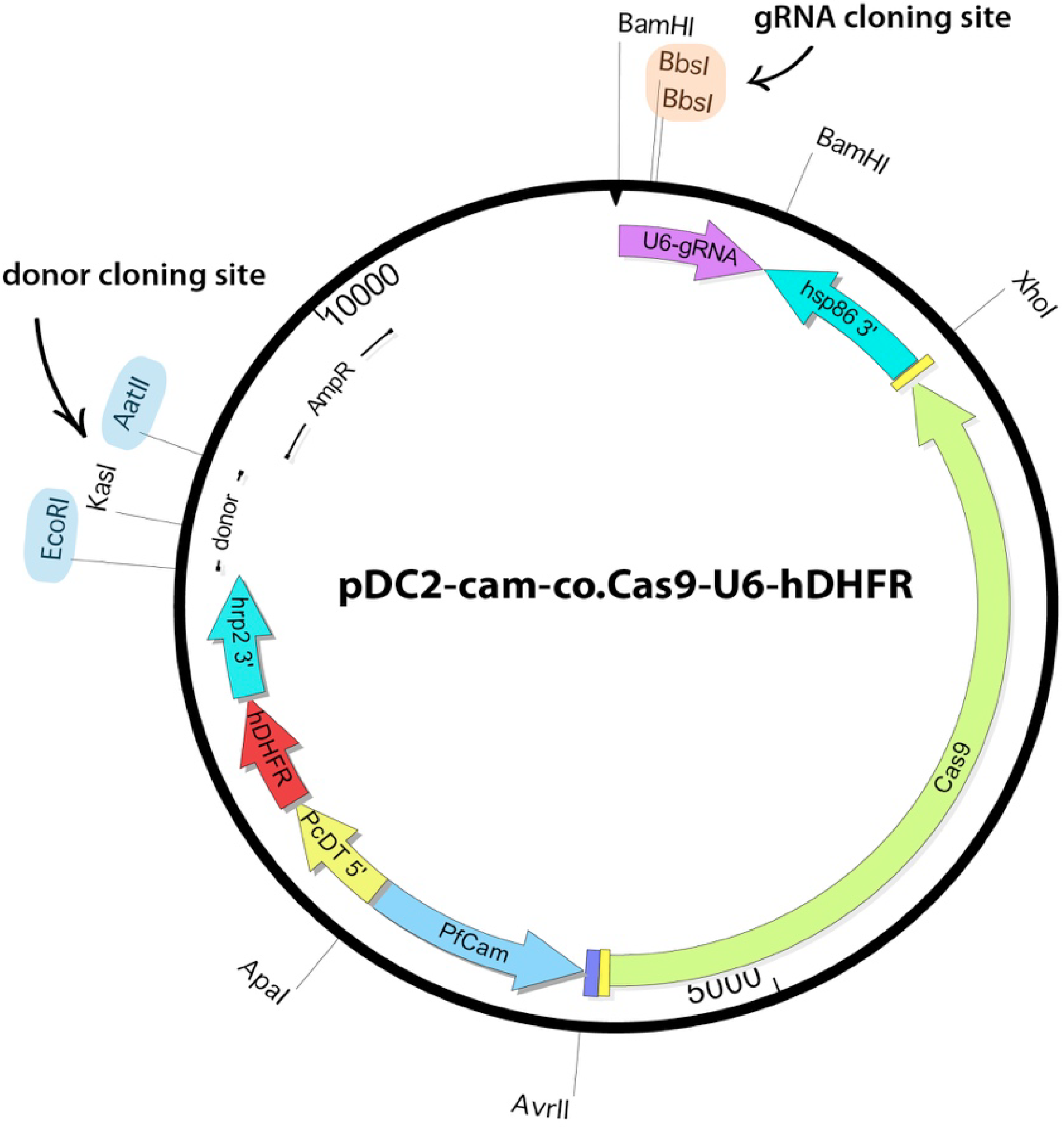

Supplementary Figure 1 | Map of the plasmid vector pDC2-cam-co.Cas9-U6-hDHFR. It was approximately 10, 000 base pairs in size and contained a Cas9 codon-optimised sequence under *Pfcam* promoter with NLS to localise it to the *P. falciparum* nucleus. The U6 cassette has *BbsI* restriction site for cloning in the gRNA. The AatII and the EcoRI restriction sites serve as the site for the donor DNA insertion. The ampicillin resistance cassette was for selection after the *E. coli* transformation and the *hDHFR* gene was for selection after the transfection. *Pfcam* - ; NLS - nuclear localisation sequences; hDHFR - human dihydrofolate reductase
