# Supplementary material for: Transcriptome-module phenotype association study implicates extracellular vesicles biogenesis in Plasmodium falciparum artemisinin resistance
Source: Front Cell Infect Microbiol. 2022 Aug 19;12:886728. doi: 10.3389/fcimb.2022.886728 (PMC9437462; doi:10.3389/fcimb.2022.886728)
Supplement: Supplementary file 1 [file DataSheet_1.zip › Supplementary_files/Supplementary Figure_2.pdf]

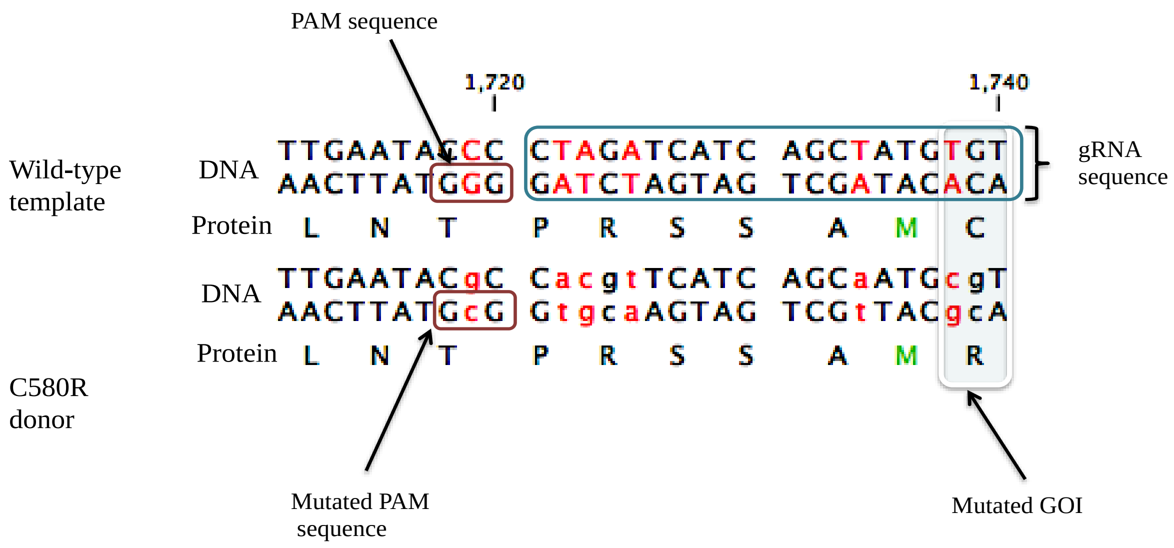

Supplementary Figure 2 | Design of donor DNA for *PfK13* genome editing. Alignment of the designed C580R donor DNA and the wild-type template (3D7), as performed with CLC Workbench, and showing the wild-type NGG PAM (GGG) sequence and the mutated PAM sequence (GCG), the gRNA sequence and the mutated GOI (gene of interest). Additional mutations were made in the donor DNA within the gRNA sequence to reduce the chance of re-cleavages.
