# Supplementary material for: Transcriptome-module phenotype association study implicates extracellular vesicles biogenesis in Plasmodium falciparum artemisinin resistance
Source: Front Cell Infect Microbiol. 2022 Aug 19;12:886728. doi: 10.3389/fcimb.2022.886728 (PMC9437462; doi:10.3389/fcimb.2022.886728)
Supplement: Supplementary file 1 [file DataSheet_1.zip › Supplementary_files/Supplementary Figure_3.pdf]

C580R

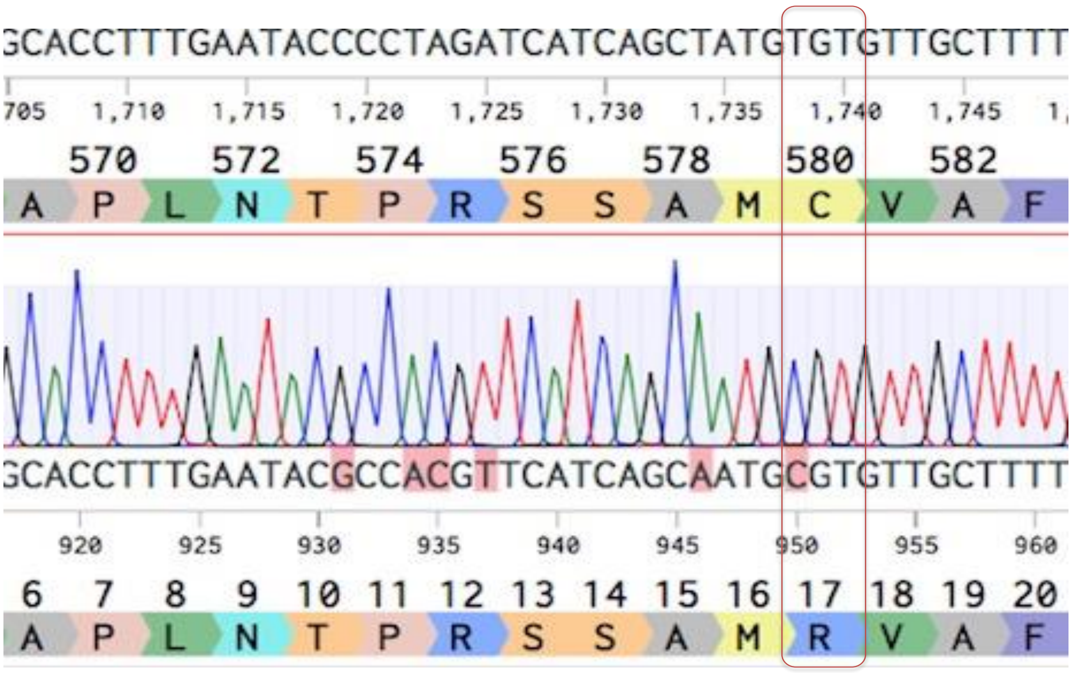

R539T

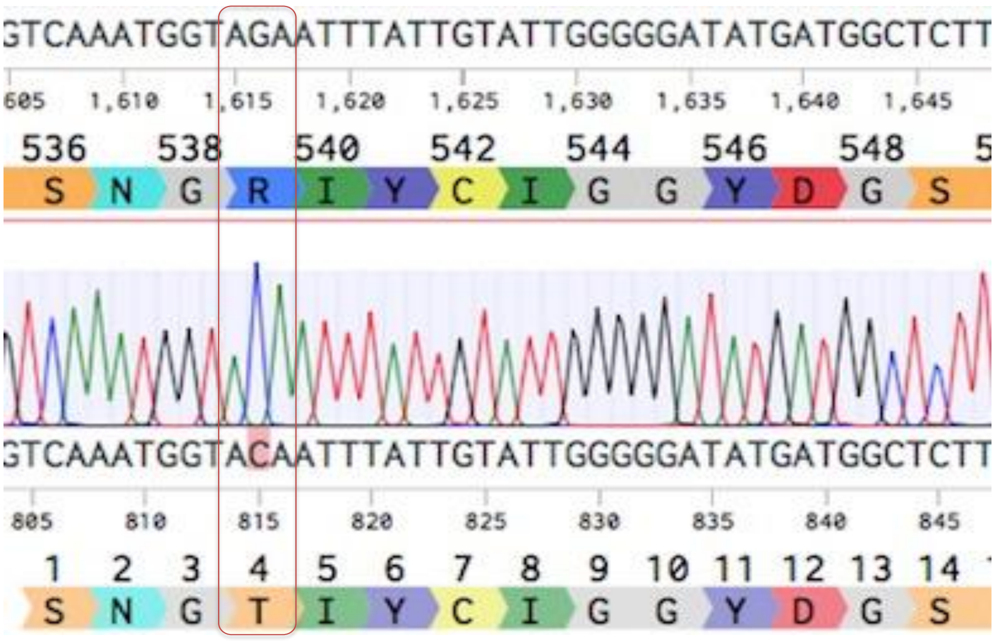

Supplementary Figure 3 | Chromatographs showing analyses of genotyped transfectants. Genotyped sequences were aligned to the 3D7 *PfK13* template using (https://www.benchling.com). Mutations seen are : AGA (R539R) to ACA (R539T); and (C580C) to CGT (C580R).
