# Supplementary material for: Transcriptome-module phenotype association study implicates extracellular vesicles biogenesis in Plasmodium falciparum artemisinin resistance
Source: Front Cell Infect Microbiol. 2022 Aug 19;12:886728. doi: 10.3389/fcimb.2022.886728 (PMC9437462; doi:10.3389/fcimb.2022.886728)
Supplement: Supplementary file 1 [file DataSheet_1.zip › Supplementary_files/Supplementary Figure_7.pdf]

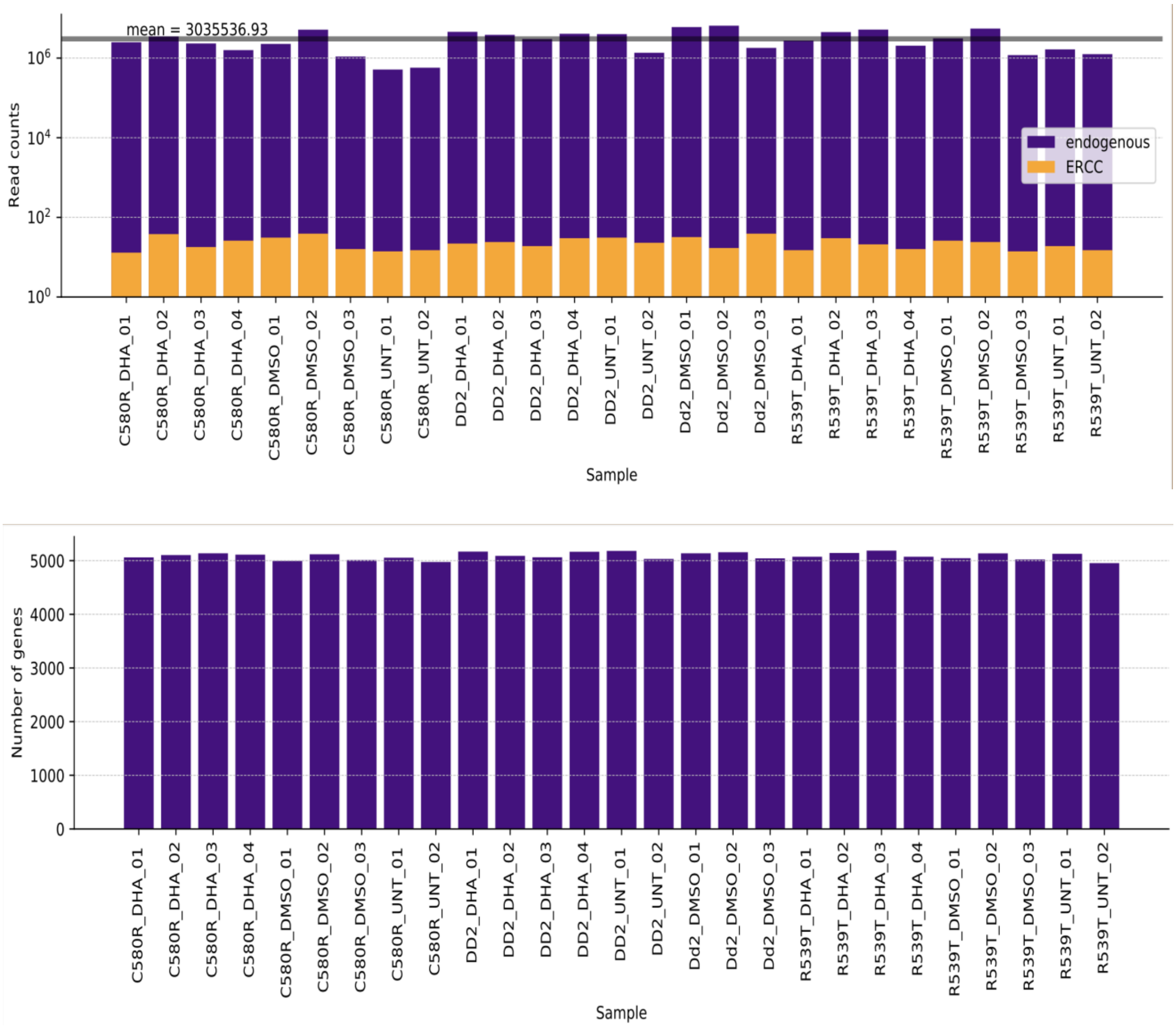

Supplementary Figure 7 | Quality of reads mapped to reference genome for all samples. Mapping quality metrics for each sample was obtained and summarised using customised R scripts. A) The mean overall alignment reads alignment to the reference *P. falciparum* transcriptome was 3035536.93 External RNA Controls Consortium (ERCC) spike-in control levels were similar across all samples. B) For each sample the number of genes, mapped to by reads, was at least 5000.
