# Supplementary material for: Transcriptome-module phenotype association study implicates extracellular vesicles biogenesis in Plasmodium falciparum artemisinin resistance
Source: Front Cell Infect Microbiol. 2022 Aug 19;12:886728. doi: 10.3389/fcimb.2022.886728 (PMC9437462; doi:10.3389/fcimb.2022.886728)
Supplement: Supplementary file 1 [file DataSheet_1.zip › Supplementary_files/Supplementary Figure_6.pdf]

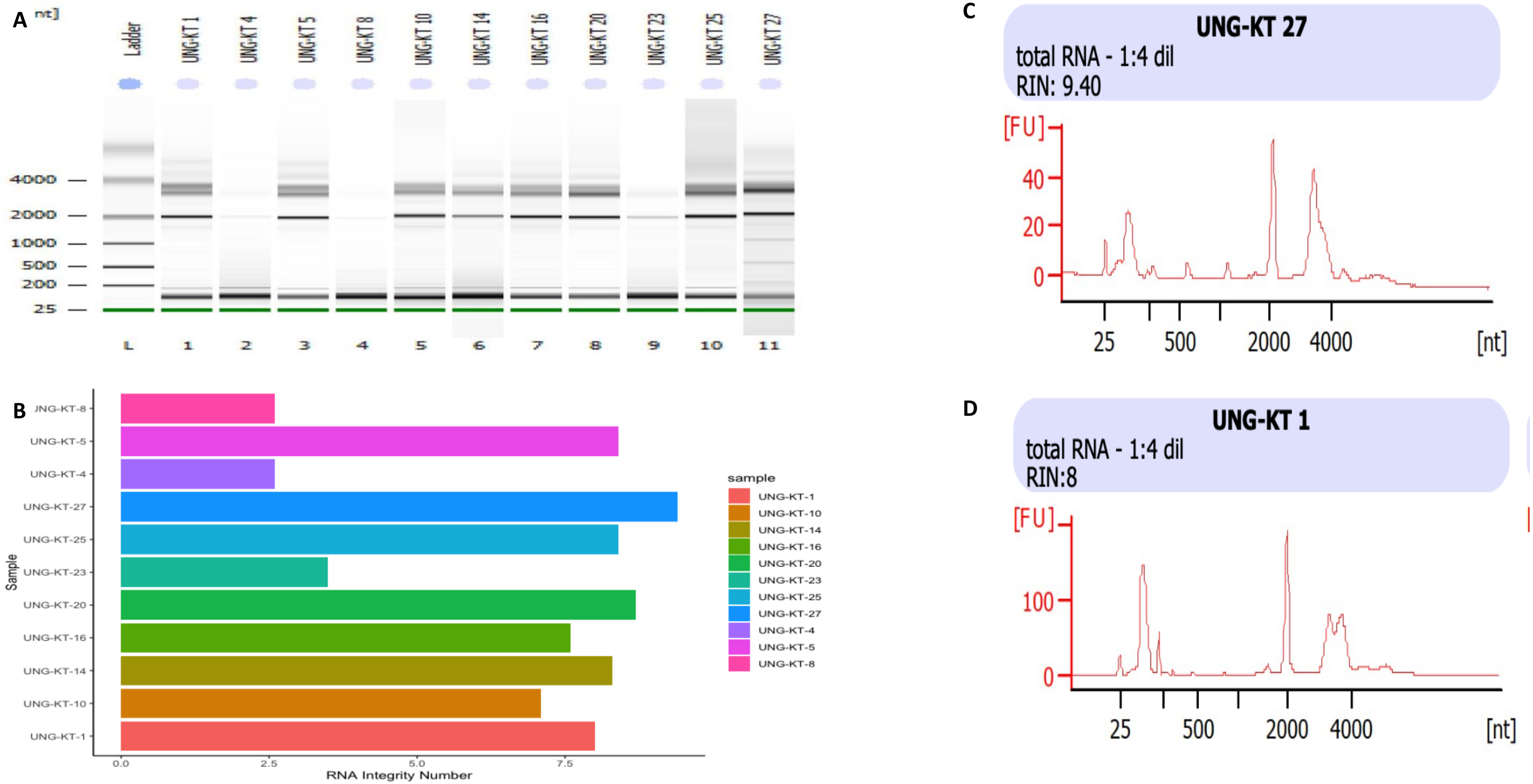

Supplementary Figure 6 | Quality of extracted RNA integrity from the 27 samples used in this study. RNA extraction was done using TRIzol method. A) Agarose electrophoretic gel to visualize 18s and 28s ribosomal RNA bands indicative of good RNA quality. B) RIN for 11 of the 27 samples analyzed. C) and D) Electropherograms for samples 27 and 1 showing good 18s and 28s peaks indicative of good RNA quality. RNA – ribonucleic acid; RIN – RNA integrity number
