# Supplementary material for: Transcriptome-module phenotype association study implicates extracellular vesicles biogenesis in Plasmodium falciparum artemisinin resistance
Source: Front Cell Infect Microbiol. 2022 Aug 19;12:886728. doi: 10.3389/fcimb.2022.886728 (PMC9437462; doi:10.3389/fcimb.2022.886728)
Supplement: Supplementary file 1 [file DataSheet_1.zip › Supplementary_files/Supplementary Figure_4.pdf]

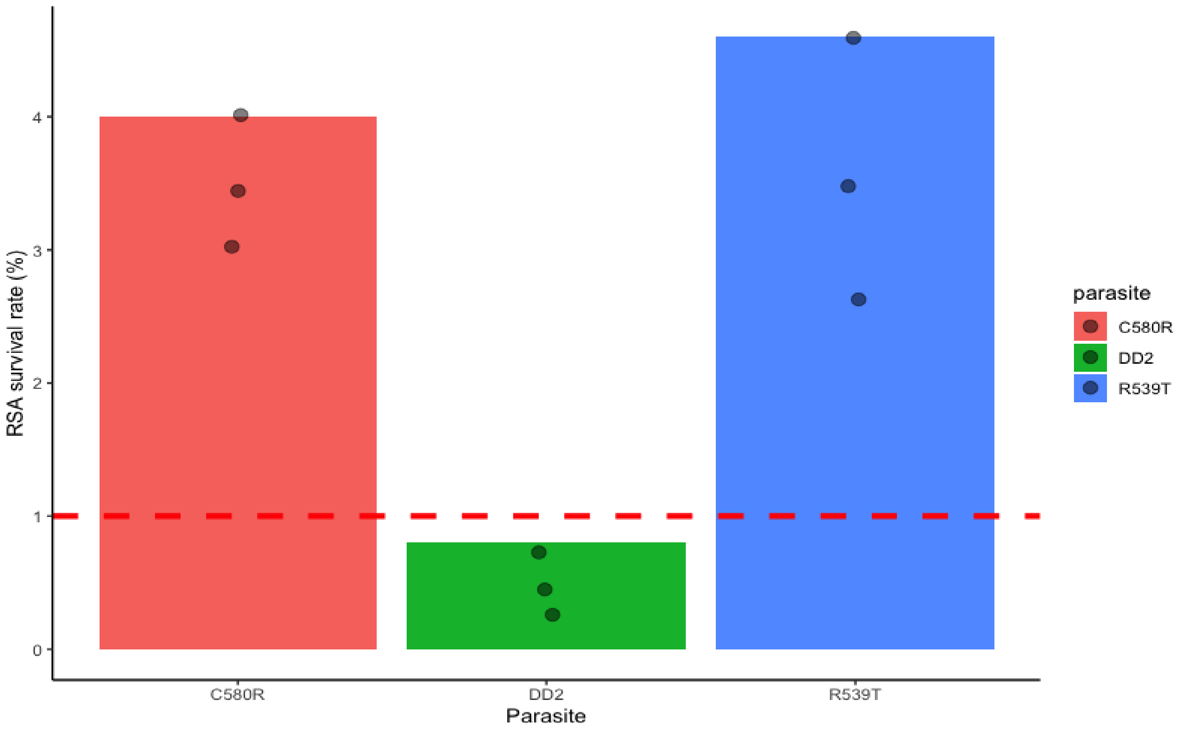

Supplementary Figure 4 | *In vitro* RSA$_{0-3}$ survival rates (%) for the ART susceptible WT *Plasmodium falciparum* strain (DD2) and two *P. falciparum PfK13* ARTr variants (C580R and R539T) used in this study. C580R and R539T are ARTr parasites generated by CRISPR/Cas9 genetic engineering of a wild type DD2 ART susceptible parasite line at the *PfK13* gene. Each bar represents the readout of triplicate experiments specified by the dots. Growth rates for all parasite lines were at least 1.5% in the non-exposed controls. Mutant *PfK13* parasites have significantly higher survival rates compared to wild type DD2 parasite line (DD2 versus C580R : p-value, Kruskal-Wallis test = 0.04; DD2 versus R539T, p-value Kruskal-Wallis test = 0.04). The red dashed line indicates the 1% survival rate above which a parasite line is characterized as ARTr. RSA$_{0-3}$ - 0-3 hour ring stage survival assay; ART – artemisinin; *PfK13 – Plasmodium falciparum* kelch 13 gene
