# Supplementary material for: Transcriptome-module phenotype association study implicates extracellular vesicles biogenesis in Plasmodium falciparum artemisinin resistance
Source: Front Cell Infect Microbiol. 2022 Aug 19;12:886728. doi: 10.3389/fcimb.2022.886728 (PMC9437462; doi:10.3389/fcimb.2022.886728)
Supplement: Supplementary file 1 [file DataSheet_1.zip › Supplementary_files/Supplementary Figure_10.pdf]

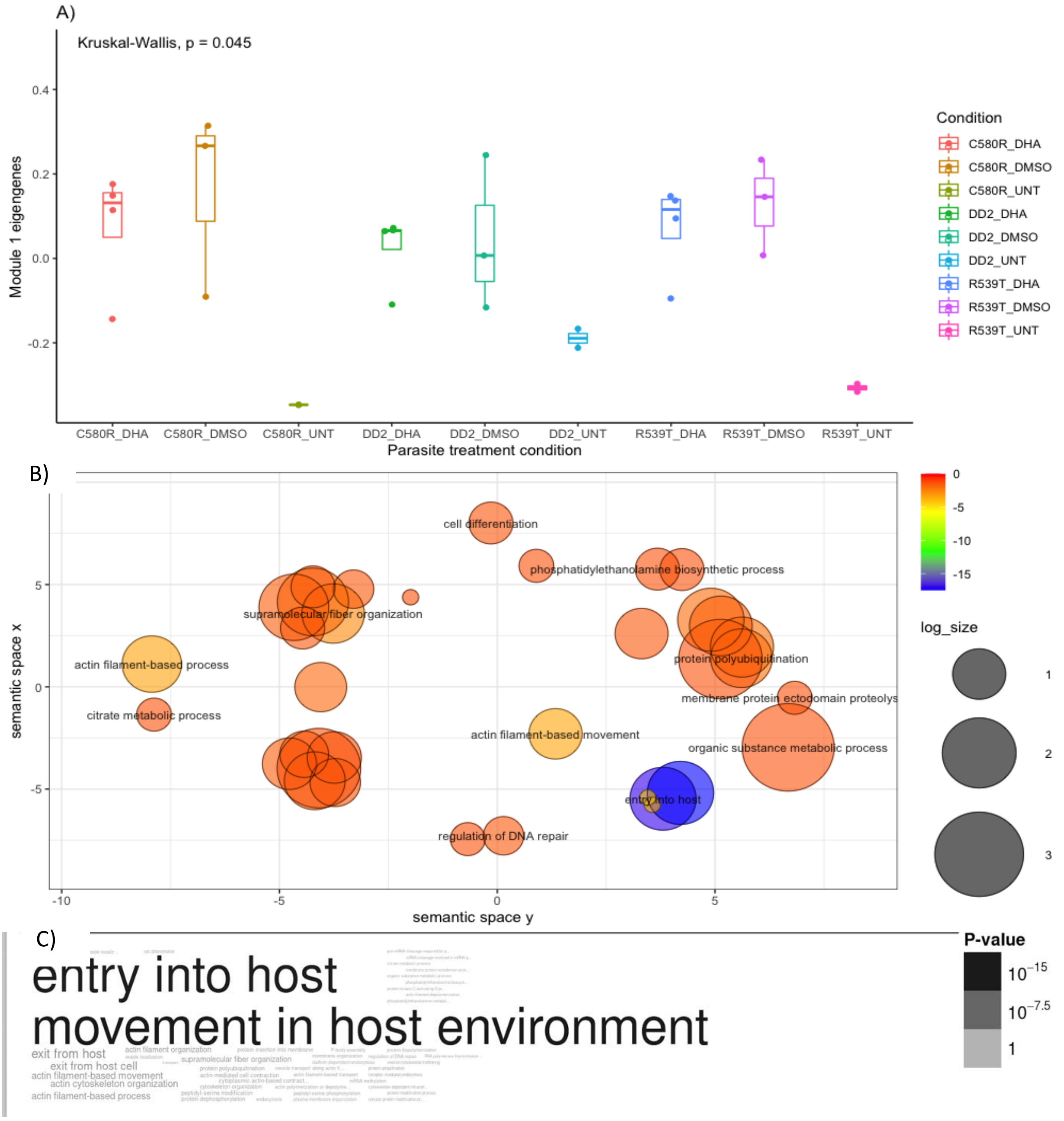

Supplementary Figure 10 | Properties of EVs module of interest module 1 (ME1). ME1 was uploaded to plasmodb (https://plasmodb.org/plasmo/app) and analyzed with the platform's gene ontology (GO) pipeline. A) Comparison of ME3 between the three treatment conditions pooled for all parasite lines (DD2, C580R and R539T). The Kruskal-Wallis test was used to determine the statistical significance of the difference in expression of ME1 among the conditions (0.05 < p-value < 0.1). B) GO (biological processes) output for ME1 summarized using REVIGO (http://revigo.irb.hr) scatterplot visualization option. Each circle denotes the representative cluster of the GO biological process associated with the ARTr phenotype and is derived by multidimensional scaling and redundancy reduction of the GO sematic similarities. The size of the circles denote the frequency of the GO term in the *P. falciparum* GO database (updated in November 2021) with larger circles denoting more general terms. The colors (red to blue range) indicate the statistical significance of the association's strength. "Movement in host environment and entry into host" were two of the biological processes enriched. C) Word cloud summary of the GO biological processes associated with the ARTr phenotype. EVs – extracellular vesicles, ARTr – artemisinin resistance
