# Supplementary material for: Transcriptome-module phenotype association study implicates extracellular vesicles biogenesis in Plasmodium falciparum artemisinin resistance
Source: Front Cell Infect Microbiol. 2022 Aug 19;12:886728. doi: 10.3389/fcimb.2022.886728 (PMC9437462; doi:10.3389/fcimb.2022.886728)
Supplement: Supplementary file 1 [file DataSheet_1.zip › Supplementary_files/Supplementary Figure_5.pdf]

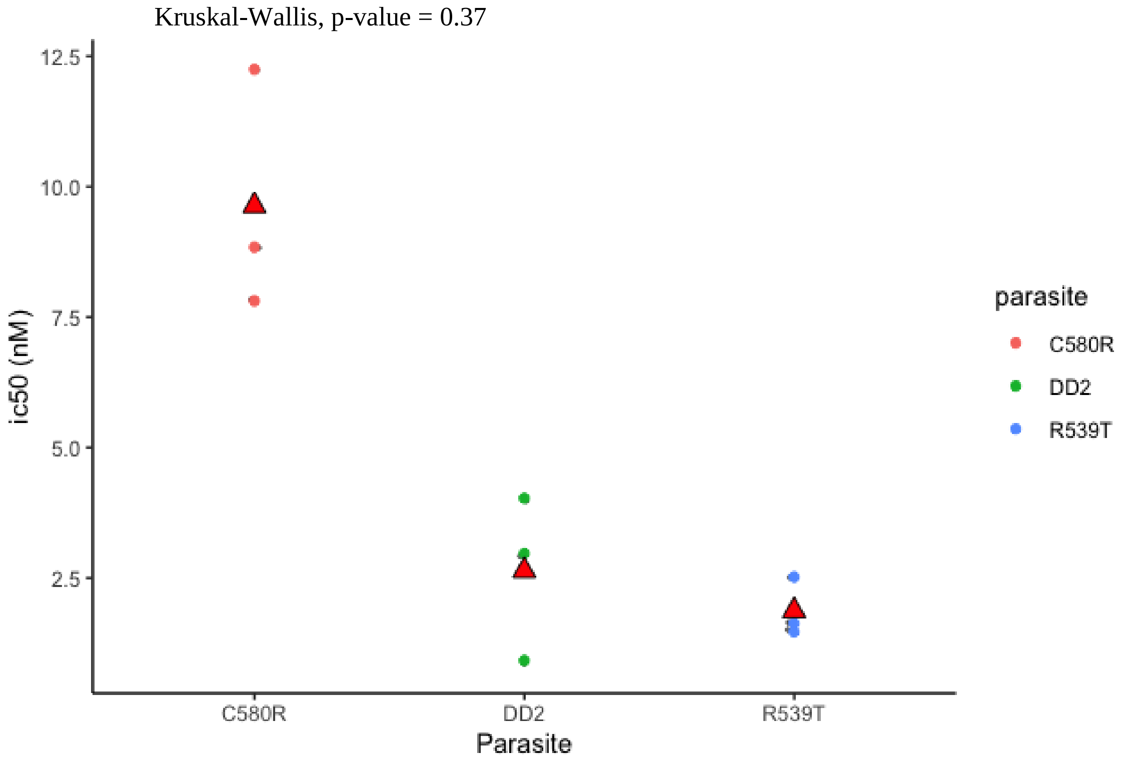

Supplementary Figure 5 | IC50 values for DHA for the ART susceptible WT *Plasmodium falciparum* strain (DD2) and two P. falciparum *PfK13* ARTr variants (C580R and R539T) used in this study. The round data point display the IC50 value for each of the triplicates for each parasite line. The red triangles represent the median IC50 values for each of the parasite lines (C580R: 8.84nM, SD = 2.32nM; R539T:1.63nM, SD = 0.57nM; DD2: 2.97nM, SD = 1.58nM). P-values were determined by Kruskal-Wallis test. Assay was done in triplicates for each parasite line. WT – wild type; IC50 - 50% inhibitory concentration; ART – artemisinin; *PfK13* – *Plasmodium falciparum* kelch 13 gene; SD – standard deviation
