# Supplementary material for: Transcriptome-module phenotype association study implicates extracellular vesicles biogenesis in Plasmodium falciparum artemisinin resistance
Source: Front Cell Infect Microbiol. 2022 Aug 19;12:886728. doi: 10.3389/fcimb.2022.886728 (PMC9437462; doi:10.3389/fcimb.2022.886728)
Supplement: Supplementary file 1 [file Supplementary Figure_8.pdf › Supplementary_files/Supplementary Figure_8.pdf]

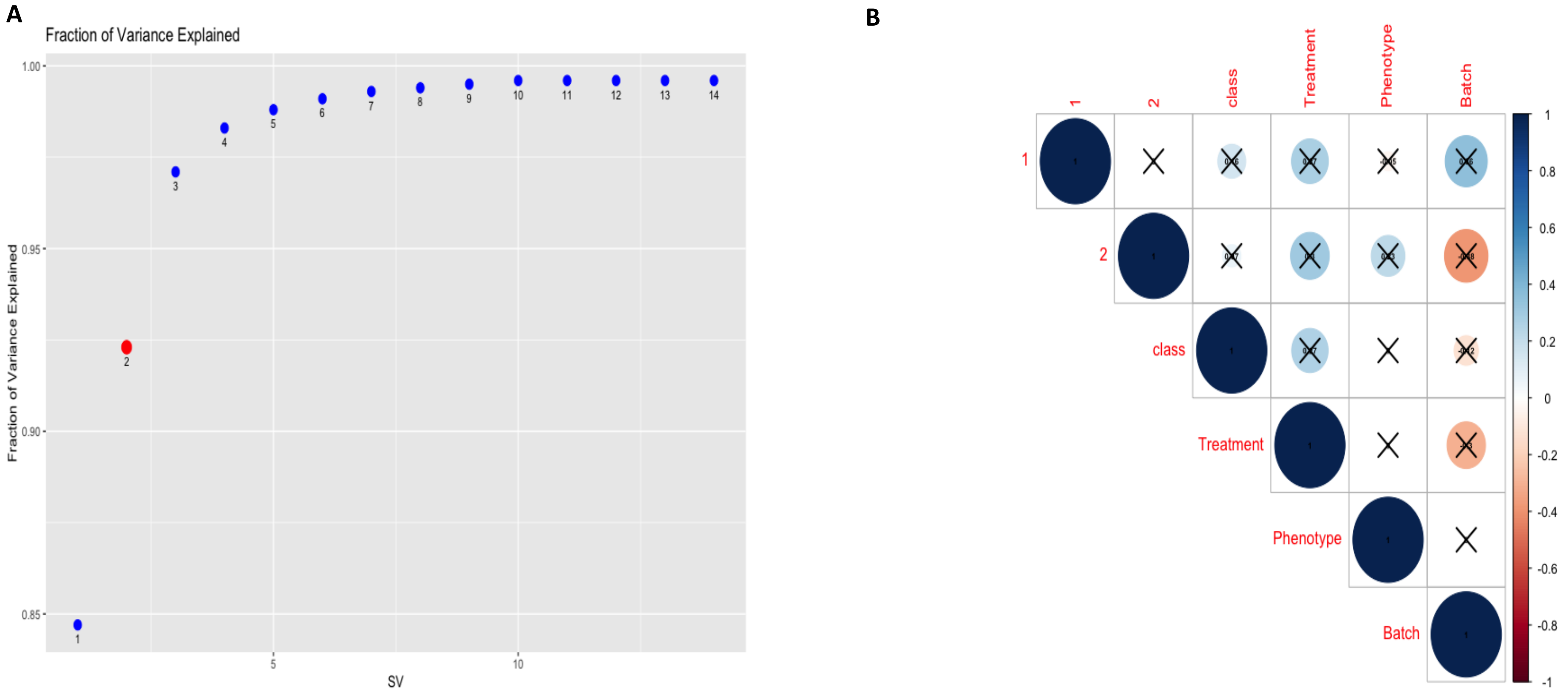

Supplementary Figure 8 | Identification of surrogate variable (Sv) using the DaMiR.SV() function in the filtered and normalized expression dataset. A) Plot showing relationship between each Sv and the fraction of the variance it explains in the data. Each blue dot represents the cumulative proportion of variance explained by a Sv. The red dot marks the Sv that corresponds to ≤ 95% of variance explained and was used to adjust the data set. B) Correlation plot between surrogate variables and known the known covariates: treatment class, parasite strain, parasite sensitivity to ART and experimental batch. This plot shows the correlation between surrogate variables and known covariates, using both color gradient and circle size. The color ranges from correlation of -1 to +1 (dark red to dark blue). The circle size is maximum for a correlation equal to 1 or -1 and decreases up to zero. Black crosses indicate non-significant correlations. This plot shows no significant correlation between all seven surrogate variables and the known covariates.
